# Bio-Based Polyhydroxyanthraquinones as High-Voltage Organic Electrode Materials for Batteries

**DOI:** 10.1021/acsapm.3c01616

**Published:** 2023-10-10

**Authors:** Tijs Lap, Nicolas Goujon, Daniele Mantione, Fernando Ruipérez, David Mecerreyes

**Affiliations:** † Joxe Mari Korta Center, POLYMAT University of the Basque Country UPV/EHU, 20018 Donostia-San Sebastiań, Spain; ‡ Centre for Cooperative Research on Alternative Energies (CIC energiGUNE), Basque Research and Technology Alliance (BRTA), Alava Technology Park, Albert Einstein 48, 01510 Vitoria-Gasteiz, Spain; § Ikerbasque, Basque Foundation for Science, 48013 Bilbao, Spain; ∥ Physical Chemistry Department, Faculty of Pharmacy, University of the Basque Country UPV/EHU, 01006 Vitoria-Gasteiz, Spain

**Keywords:** bio-based, high voltage, organic batteries, lithium metal battery, organic electrode material

## Abstract

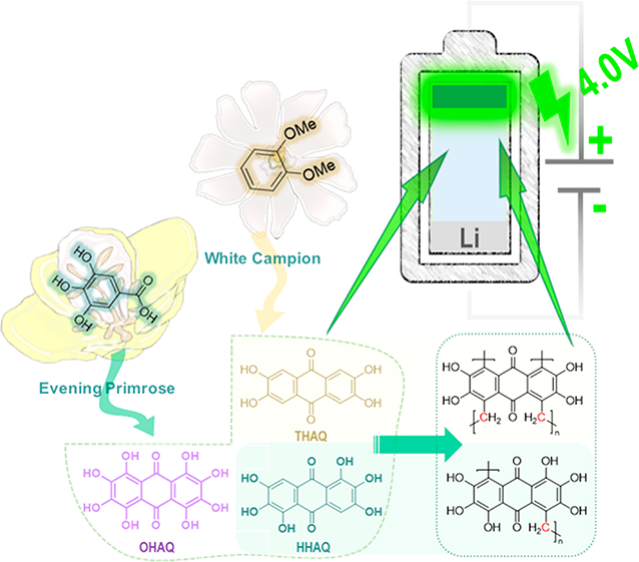

Organic materials have gained much attention as sustainable electrode materials for batteries. Especially bio-based organic electrode materials (OEMs) are very interesting due to their geographical independency and low environmental impact. However, bio-based OEMs for high-voltage batteries remain scarce. Therefore, in this work, a family of bio-based polyhydroxyanthraquinones (PHAQs)—namely 1,2,3,4,5,6,7,8-octahydroxyanthraquinone (OHAQ), 1,2,3,5,6,7-hexahydroxyanthraquinone (HHAQ), and 2,3,6,7-tetrahydroxyanthraquinone (THAQ)—and their redox polymers were synthesized. These PHAQs were synthesized from plant-based precursors and exhibit both a high-potential polyphenolic redox couple (3.5–4.0 V vs Li/Li^+^) and an anthraquinone redox moiety (2.2–2.8 V vs Li/Li^+^), while also showing initial charging capacities of up to 381 mAh g^–1^. To counteract the rapid fading caused by dissolution into the electrolyte, a facile polymerization method was established to synthesize PHAQ polymers. For this, the polymerization of HHAQ served as a model reaction where formaldehyde, glyoxal, and glutaraldehyde were tested as linkers. The resulting polymers were investigated as cathode materials in lithium metal batteries. PHAQ polymer composites synthesized using formaldehyde as linker and 10 wt % multiwalled carbon nanotubes (MWCNTs), namely poly(THAQ–formaldehyde)–10 wt % MWCNTs and poly(HHAQ–formaldehyde)–10 wt % MWCNTs, exhibited the best cycling performance in the lithium metal cells, displaying a high-voltage discharge starting at 4.0 V (vs Li/Li^+^) and retaining 81.6 and 77.3 mAh g^–1^, respectively, after 100 cycles.

## Introduction

Over the past decades, lithium-ion batteries (LIBs) have become inseparably interwoven into our society. Moreover, the demand for LIBs is expected to increase tremendously due to their use in rapidly expanding sectors, such as portable electronics, large-scale energy storage, and electric vehicles.^1−7^ Despite LIBs being the state-of-the-art battery chemistry, their utilization of scarce metals (e.g., cobalt, nickel, and manganese) as electrode materials raises various concerns. These concerns range from the scarcity of these elements and their topo geographical limited harvesting to the footprint of their energy-intensive mining and processing.^2^

As a result, organic electrode materials (OEMs) have gained much attention as sustainable alternatives.^2^ This is mainly due to the abundance of their elements, their energy-efficient processing, and the ease of their modification by organic chemistry.^3−5^ Especially bio-based OEMs are very promising because of their geographical independent harvesting and the even lowered environmental impact of their life cycles compared to those of many regular OEMs, which are often still derived from the fossil fuel feedstock.^6^ In terms of performance, organic materials stand out foremost by demonstrating impressive capacities and great rate capabilities. For example, among the many reported OEMs, the intensively studied anthraquinone- and catechol-based polymers can deliver capacities of up to 200 and 360 mAh g^–1^, respectively, while LIBs usually deliver capacities of around 140 mAh g^–1^.^7−10^

However, despite high theoretical capacities, only few materials have been reported to display high redox potentials of >3.5 V (vs Li/Li^+^), necessary for high-power batteries. These OEMs include thianthrene and TEMPO-based polymers, which however are not bio-based.^11−14^ Anthraquinone (AQ) polymers typically show a moderate redox potentials around 2.5 V (vs Li/Li^+^).^15,16^ Indeed, this redox potential can be altered by the introduction of substituents to the anthraquinone scaffold. Whenever these substituents are electron-donating groups (EDGs) or electron-withdrawing groups (EWG), the anthraquinone redox potential will be respectively lowered or elevated.^17,18^ Interestingly, if these substituents themselves are redox-active, covalently linking them to the anthraquinone structure can allow the introduction of an additional redox moiety. For example, several studies demonstrated the possibility of introducing additional high-voltage functionalities to the anthraquinone core, realizing an extra redox couple with high reduction potentials in addition to that of the anthraquinone carbonyls. One example is the insertion of redox-active amide linkers in conjugated anthraquinone polymers, which displayed a discharge plateau as high as 3.7 V (vs Li/Li^+^), not affecting the redox of the anthraquinone.^9,19^ These findings open opportunities for synthesizing high-voltage OEMs by introducing high-potential redox moieties to the anthraquinone molecule. Moreover, theoretically the redox potential of these moieties will be elevated by the presence of the electron-withdrawing anthraquinone carbonyls because the latter lowers the electron density, pushing both the oxidation and reduction potentials to higher values.^7,8^ Hence, the introduction of hydroxyl groups on the anthraquinone rings, creating polyphenolic moieties, might generate additional high-voltage behavior. For example, the aforementioned catechol group already owns relatively high redox potentials of 3.2–3.4 V (vs Li/Li^+^), which can be elevated by the presence of the electron-withdrawing carbonyls of the anthraquinone. Moreover, the repulsion of two similarly oriented dipoles in a cyclic 1,2-dicarbonyl compound is known to destabilize the oxidized form and eventually lead to higher reduction potentials as well.^20^

The aim of this work was to synthesize a family of bio-based polyhydroxyanthraquinones (PHAQs)—namely 1,2,3,4,5,6,7,8-octahydroxyanthraquinone (OHAQ), 1,2,3,5,6,7-hexahydroxyanthraquinone (HHAQ), and 2,3,6,7-tetrahydroxyanthraquinone (THAQ)—and their redox polymers as high-voltage organic electrode materials for batteries. To address the need for environmentally friendly OEMs, synthesis pathways starting from bio-based precursors were selected to realize high-voltage and bio-based OEMs. In terms of redox behavior, it was rationalized that the introduction of phenol groups on an anthraquinone scaffold (2, 3, or 4 OH groups per anthraquinone periphery ring) could give rise to a set of OEMs with high discharge potentials (>3.5 V vs Li/Li^+^). Higher numbers of phenol groups would lower the reduction potential by nature of the electron-donating groups, counteracting the electron-withdrawing influence of the anthraquinone core. Moreover, combining the anthraquinone and polyphenolic redox functionalities in one molecule could give rise to bio-based OEMs with both a high-voltage component and very high theoretical capacities, dependent on the degree of utilization of both substructures. Therefore, to examine their electrochemical characteristics, the family of PHAQs was subjected to cyclic voltammetry (CV) and Li half-cell cycling, and the influence of the hydroxylation degree on the polyphenolic redox potential was concisely investigated, both empirically and *in silico*. Finally, to enhance the cycling stability, the synthesis of polymers using phenol–formaldehyde chemistry was investigated. For this, the polymerization of HHAQ using phenol–formaldehyde chemistry was used as model reaction. The influence on the final material performance of different utilized aldehyde linkers and the presence of multiwalled carbon nanotubes (MWCNTs) was investigated as cathodes in lithium metal batteries.

## Experimental Section

### Materials

Reagents and reactants were purchased from Fisher Scientific or Sigma-Aldrich and used without further purification. For further details see Section SI-I-1. 2,3,6,7-Tetrahydroxyanthraquinone, 1,2,3,5,6,7-hexahydroxyanthraquinone, and 1,2,3,4,5,6,7,8-octahydroxyanthraquinone were synthesized according to the literature, and their procedures and ^1^H NMR, ^13^C NMR, and ATR-FTIR spectra are shown in Section SI-I-3.^21−23^

### General Procedure Synthesis of Poly(THAQ–formaldehyde) and Poly(HHAQ–formaldehyde)

In a 10 mL round-bottom flask equipped with a magnetic stirrer, 0.2 g of PHAQ was dissolved in *X* mL of concentrated H_2_SO_4_ and *Y* mL of glacial acetic acid (for *X* and *Y*, see Table 1). The resulting mixture was heated to 90 °C while stirring, and 8.0 equiv of formaldehyde (37 wt % aqueous) was added subsequently in a dropwise fashion. The solidified reaction mixture was added to 200 mL of demineralized water followed by addition of NaOH until pH = 6–7. The polymer was obtained via filtration under reduced pressure, subsequently washed with hot THF (1–3× 100 mL) until no colored monomer solution was obtained anymore, filtered, and dried overnight under vacuum at 60 °C.

**Table 1:** Utilized Ratios of Concentrated H_2_SO_4_ and Glacial Acetic Acid for the Synthesis of Poly(HHAQ–aldehyde) and Its 10 wt % MWCNTs Composites, Where *X* and *Y* Are in mL

	ratio 1	ratio 2	ratio 3
*X* (conc H_2_SO_4_)	1.2	2.0	2.8
*Y* (glacial acetic acid)	1.6	0.8	

Detailed synthesis procedures of poly(HHAQ–formaldehyde), poly(HHAQ–formaldehyde)/10 wt % composite, poly(HHAQ–glyoxal), poly(HHAQ–glutaraldehyde), poly(THAQ–formaldehyde), and poly(THAQ–formaldehyde)/10 wt % composite and the corresponding ATR-FTIR spectra are shown in Section SI-I-3.

### Electrochemical Testing

Cyclic voltammetry of the THAQ, HHAQ, OHAQ, or the polymers or their MWCNTs composites was performed in a lithium metal coin cell or three-electrode cell using a glassy carbon working electrode and lithium metal strips as both counter and reference electrode. Lithium metal batteries, based on cathodes of THAQ, HHAQ, and OHAQ, or the polymers or their MWCNTs composites, were assembled inside an argon-filled glovebox. For detailed descriptions of electrode formulations, lithium metal battery setups, and employed electrolytes see Section SI-I-2.

### Quantum Chemical Calculations

Geometry optimizations have been performed in gas phase within density functional theory (DFT) using the B3PW91 functional, together with the 6-31+G(d,p) basis set.^24−27^ Harmonic vibrational frequencies were obtained to confirm that all structures were minima in the potential energy surface and then used to evaluate the thermal (*T* = 298 K) corrections to the enthalpy and Gibbs free energy in the harmonic oscillator approximation. Single-point calculations using the aug-cc-pVTZ basis set were performed on the optimized structures to refine the electronic energy.^28^ Solvent effects in methanol have been estimated using the polarizable continuum model (PCM) approach.^29−32^ All calculations were performed by using the Gaussian 16 suite of programs.^33^ See Section SI-I-2 for a detailed description of the computational calculations.

## Results and Discussion

### Synthesis and Characterization of Polyhydroxyanthraquinone (PHAQ) Molecules

**Scheme 1:**
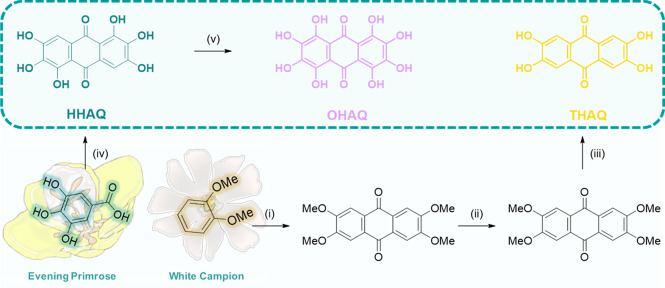
Synthesis Scheme of the Polyhydroxyanthraquinone Family Containing 2,3,6,7-Tetrahydroxyanthraquinone (THAQ), 1,2,3,5,6,7-Hexahydroxyanthraquinone (HHAQ), and 1,2,3,4,5,6,7,8-Octahydroxyanthraquinone (OHAQ) Reagents and conditions: (i) H_2_SO_4_ (72% aq), acetaldehyde (2.4 equiv); (ii) acetic acid (72% aq), Na_2_Cr_2_O_7_·2H_2_O (5.5 equiv); (iii) HBr (48% aq); (iv) conc H_2_SO_4_, and (v) conc H_2_SO_4_, H_3_BO_3_ (6.55 equiv), HgO (0.033 equiv).

Three different polyhydroxyanthraquinone small molecules were synthesized from the respective bio-based precursors, as described in the literature.^21−23^ HHAQ and OHAQ were synthesized from gallic acid, which can be extracted enzymatically from several plants (e.g., Evening Primrose or bearberry leaves) up to 309 mg/g using tannase.^34^ THAQ was synthesized from Veratrole, of which the excretion by the White Campion plant can be increased by nutrition containing key constituents of the biosynthesis.^35^ The syntheses are summarized in Scheme 1. All compounds were characterized by ^1^H NMR, ^13^C NMR, and ATR-FTIR (see Section SI-I-3) and corresponded to the literature.

### Electrochemical Characterization of PHAQs

**Figure 1:**
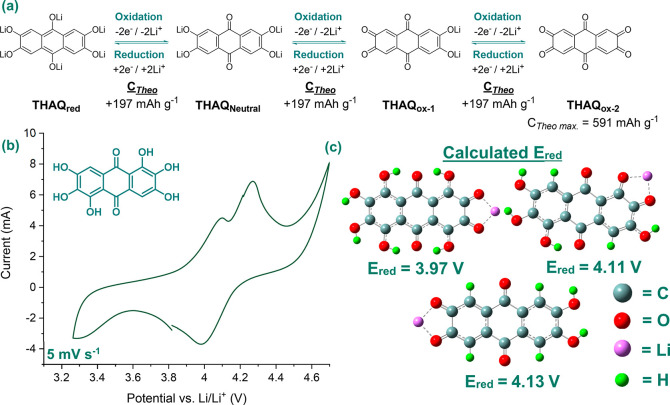
(a) Anticipated electrochemical mechanism of THAQ including the oxidized (THAQ_ox-1_ and THAQ_ox-2_) and reduced (THAQ_red_) states and their related theoretical capacities (*C*_Theo_). (b) Cyclic voltammogram (scan rate = 5 mV/s) of the polyphenolic subgroup of HHAQ in a three-electrode setup using Li metal as counter and reference electrode and a [HHAQ:C_65_:SEBS] = [40:50:10] electrode formulation on glassy carbon as working electrode with 1.0 M LiPF_6_ in EC:DEC (1:1, v/v) as electrolyte. (c) Molecular structures and *E*_red_ of THAQ, HHAQ, and OHAQ obtained from DFT calculations.

The anticipated electrochemical mechanism of THAQ and the theoretical capacities (*C*_Theo_) related to the different molecular oxidation states are depicted in Figure 1a (for HHAQ and OHAQ, see Section SI-II-4). THAQ was synthesized in the neutral state (THAQ_neutral_) in which the anthraquinone moiety is in its oxidized state and the catechol subgroup is in its reduced state. Upon completion of the synthesis, the polyphenolic moieties are in their protonated state. However, because Li^+^ electrolytes are employed during CV and lithium half-cell cycling, Li^+^ ions are depicted as counterion for THAQ_neutral_ in Figure 1a. Note that upon the first oxidation–reduction cycle a proton–Li^+^ exchange will occur due to the significantly higher concentration of Li^+^ with respect to the H^+^ concentration in the electrolyte. When used as a cathode material, THAQ_neutral_ will be oxidized to THAQ_ox-2_ during charging and reduced to THAQ_red_ upon discharge. Furthermore, the oxidation from THAQ_neutral_ to THAQ_ox-2_ could happen via an initial oxidation step to THAQ_ox-1_ afore completely oxidizing the THAQ molecule to THAQ_ox-2_. The voltammogram shown in Section SI-II-4 supports the hypothesis of a two-step oxidation process as two peaks appear during oxidation of the catechol subgroups.

Dependent on the degree of utilization of the different redox moieties, theoretical capacities of 197 (2e^–^ process), 394 (4e^–^ process), or 591 mAh g^–1^ (6e^–^ process) can be obtained. Similarly, for HHAQ the *C*_Theo_ ranges from 176 to 352 and 529 mAh g^–1^ for a 2-, 4-, or 6-electron process, respectively, while for OHAQ these values are 159, 319, 478, 638, and 797 mAh g^–1^. From these values, it becomes clear that these materials are potentially interesting not only because of their possible high-voltage OEM for high-power batteries but also due to their theoretically high capacities.

**Figure 2:**
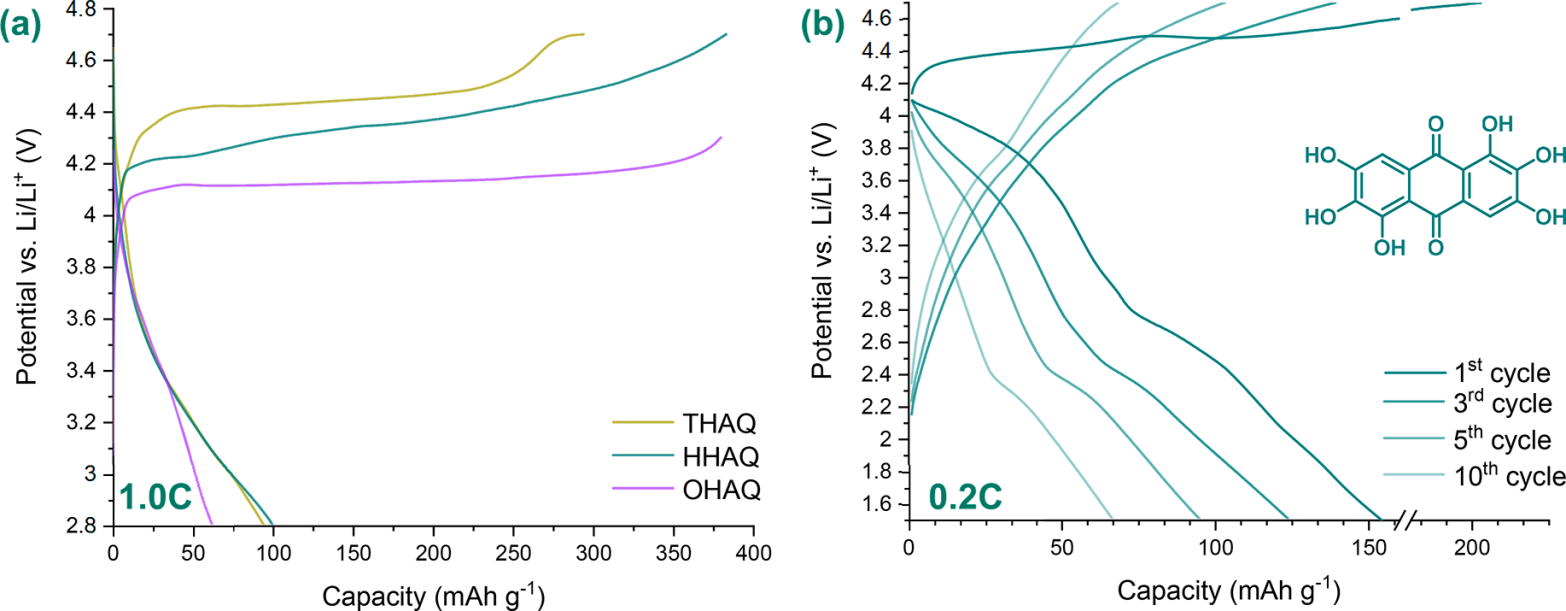
(a) First galvanostatic charge–discharge cycle (C-rate = 1C) of THAQ, HHAQ, and OHAQ in Li half-cells, using [Act. Mat.:C_65_:PVDF] = [40:40:20] electrode formulations and 1.0 M LiPF_6_ in EC:DEC (1:1, v/v) as electrolyte. (b) First 10 galvanostatic charge–discharge cycles (C-rate = 0.2C) of HHAQ in Li half-cells, using [Act. Mat.:C_65_:SEBS] = [40:50:10] as electrode formulation and 0.3 M LiTFSI/[PY13][TFSI] ionic liquid as electrolyte.

Although THAQ and HHAQ have been tested and employed as respective anolyte for alkaline redox flow batteries and anode for LIBs, a full electrochemical characterization as cathode OEM for lithium metal batteries has not been reported.^36,37^ Therefore, all synthesized PHAQs were subjected to cyclic voltammetry, *in silico* analysis, and lithium half-cell cycling, as is shown in Figure 1, Figure 2, and Section SI-II-4.

The voltammogram of the polyphenolic subgroup of HHAQ is shown in Figure 1b (see Section SI-II-4 for the THAQ and OHAQ). Both HHAQ and THAQ exhibit a high-potential two-step oxidation of the polyphenolic moiety at 4.1 and 4.3 V (vs Li/Li^+^) for HHAQ and 4.15 and 4.3 V (vs Li/Li^+^) for THAQ, while having a high-potential one-step reduction at 4.0 and 3.85 V (vs Li/Li^+^), respectively. The oxidation and reduction process of OHAQ is slightly more complex because two major and one minor oxidation peak at 4.1, 4.2, and 4.3 V (vs Li/Li^+^) can be observed, combined with a multipeak reduction at 4.0, 3.8, and 3.5 V (vs Li/Li^+^). This complexity most likely relates to the presence of additional hydroxyl groups. Nevertheless, the high-voltage redox activity of all three PHAQs makes them interesting for deployment as OEMs for high-power batteries. Although 1.0 M LiPF_6_ in EC:DEC (1:1, v/v) is known to be less suitable for anthraquinone-based polymers, this electrolyte was selected for this study after a concise screening because it was found most suitable for the polyphenolic redox reaction.^38^ Nevertheless, the voltammograms of the AQ region demonstrate significant separations between the oxidation and reduction peaks for all of the PHAQs (see Section SI-II-4).

The high-voltage behavior of these PHAQs was further investigated *in silico* by using density functional theory (DFT) calculations. The calculated polyphenolic reductions potentials (*E*_red_) for THAQ, HHAQ, and OHAQ were 4.13, 4.11, and 3.97 V (vs Li/Li^+^), respectively. These findings support the postulate that the presence of the electron withdrawing anthraquinone carbonyls elevates the redox potential of the polyphenolic subgroups. Moreover, a decreasing trend in the reduction potential was observed with increasing degrees of hydroxylation of the anthraquinone periphery rings. This is in line with the hypothesis that the electron-donating OH functionalities counteract the effect of the electron withdrawing ketones of the anthraquinone scaffold, leading to lower reduction potentials. Although the difference in the calculated reduction potentials is minimal, they are in contrast with those determined by cyclic voltammetry because THAQ displays the lowest *E*_red_. Multiple factors including electrode formulation, possible over potentials, and different electrolyte–PHAQ interactions possibly explain the minor differences between the experimental discharge potentials and those obtained by the DFT calculations.

Despite the fact that the presence of the high reduction potentials makes these PHAQs interesting OEMs for high-energy LIBs, strong dissolution became evident during cyclic voltammetry experiments (see Section SI-II-4) and the lithium half-cell cycling in the polyphenolic redox range (>3.8 V vs Li/Li^+^). Therefore, during the cyclic voltammetry experiments, different potentials ranges were used to visualize the AQ and polyphenolic redox processes distinctively due to the solubility issue faced with the small molecules after the oxidation of the phenolic moieties. Moreover, fresh electrodes on glassy carbon were prepared for each measurement. The dissolution of active material at >3.8 V (vs Li/Li^+^) can be attributed to the improved electrostatic interaction between the carbonate-based electrolyte and the oxidized polyphenolic groups. Consequently, a dissolution rate trend of OHAQ > HHAQ > THAQ could be observed visually while performing cyclic voltammetry above 3.8 V (vs Li/Li^+^), where dissolution rates increased with higher hydroxylation degrees of the anthraquinone core. In lithium metal batteries, this dissolution caused a poor discharge capacity following an initial charge with capacities of up to 381 mAh g^–1^ during the first cycle for all PHAQs when being cycled at 1.0C with 1.0 M LiPF_6_ in EC:DEC as electrolyte (see Figure 2a). Additionally, no high-voltage plateau could be observed during this cycling for any small molecule. Moreover, although the obtained capacities upon the first charge are high, these capacities are significantly lower than the calculated theoretical capacities (see Figure 1a and Section SI-II-4). This could be due to several factors such as limited electrolyte access into the electrode, suboptimal carbon-active material contact, or partial utilization of the redox centers in the PHAQs (e.g., one of the two polyphenolic moieties).

To overcome the capacity fading due to dissolution, THAQ, HHAQ, and OHAQ were subsequently cycled at 0.2C in lithium half-cells employing 0.3 M LiTFSI/[PY13][TFSI] ionic liquid as electrolyte (see Figure 2b for HHAQ and Section SI-II-4 for THAQ and OHAQ). While the discharge plateaus of both the polyphenolic and anthraquinone redox centers became visible during galvanostatic cycling under these conditions for all three PHAQs, swift capacity fading was still observed for HHAQ and OHAQ (57% and 69%, respectively, after 10 cycles). Contrarily, THAQ, albeit demonstrating lower initial capacities, displayed a 45% capacity increase during the first 10 cycles, followed by a steady fading. The observed difference in capacity fading rate is in agreement with the earlier described dissolution rate trend and follows the same explanation.

### Polymerization of polyhydroxyanthraquinone (PHAQ) Molecules

**Scheme 2:**
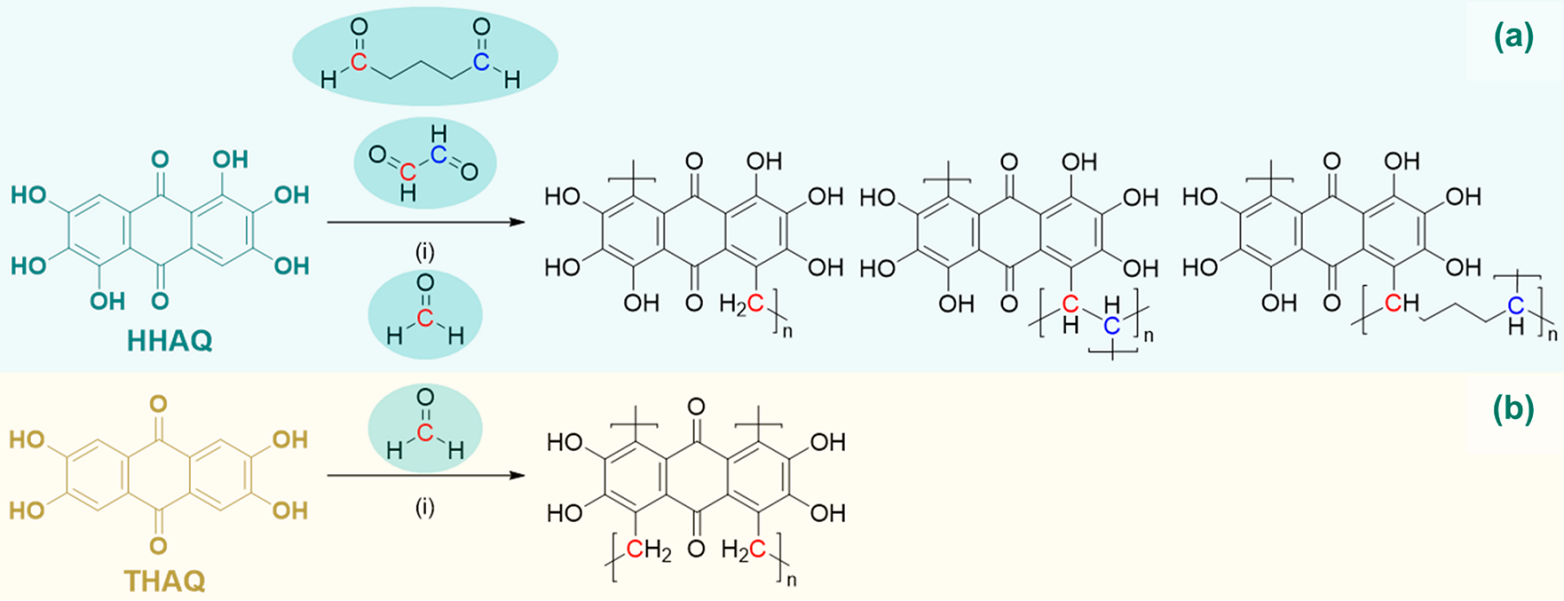
(a) Condensation–Polymerization of (a) HHAQ and (b) THAQ Utilizing Formaldehyde, Glyoxal, or Glutaraldehyde as Linker Reagents and conditions: (i) conc H_2_SO_4_, 10 wt % MWCNTs, 90 °C, 42 h.

In quest of addressing rapid capacity fading due to dissolution of the active material into the electrolyte, polymerization is an often-applied strategy for OEMs.^39^ Ultimately, a polymerization method using formaldehyde as the linker under strongly acidic conditions enabled the synthesis of poly(THAQ–formaldehyde) and poly(HHAQ–formaldehyde), as shown in Scheme 2.

The mechanism of this type of reaction has been studied in the literature, and several similar polyphenolic monomers were polymerization under similar conditions for different types of applications.^40−42^ Although some benzoquinone scaffolds have been polymerized using this approach, to the best of our knowledge, this is the first time an anthraquinone-based monomer has been polymerized using phenol formaldehyde polymer chemistry, opening yet another synthetically pathway for anthraquinone-derived polymers.^43^

Inspired by the aforementioned studies, Section SI-II-1 displays the anticipated mechanism for this condensation–polymerization of the HHAQ monomer. Indeed, this mechanism explains why this method fails to deliver polymers of OHAQ, as it does not possess any free positions on the anthraquinone core.

Initial polymerizations used similar conditions to those typically found in the literature.^42^ These conditions included a 0.34 M concentration of HHAQ in a 1:1.33 (v:v) mixture of concentrated H_2_SO_4_ and glacial acetic acid and 1.0 equiv of formaldehyde (37 wt % aq) at 90 °C for 24–42 h. ^1^H NMR and MALDI-TOF characterizations of the obtained product were attempted; however, both methods did not provide any satisfactory results. Nonetheless, ATR-FTIR analysis was successfully utilized to confirm the polymerization. The polymer synthesis was optimized by using indirect feedback on the polymer performance obtained from galvanostatic cycling of the battery. Here the cycling stability and the presence of the high-voltage polyphenolic discharge were key parameters.

Although the first polymerization attempt provided poly(HHAQ–formaldehyde), its electrochemical performance was poor; despite improved cycling stability with respect to the HHAQ monomer, the capacity fading remained severe, and no high-voltage discharge plateau was noted (see Section SI-II-4). From this, it was concluded that the polymerization required optimization.

Therefore, reaction conditions were systematically screened in the pursuit of polymers with improved electrochemical performances. To this end, the polymerization of HHAQ was used as a model reaction, after which these optimized conditions were used to synthesize poly(THAQ– formaldehyde). Table 2 in Section SI-I-3 summarizes the independently varied parameters, including different reaction temperatures, cross-linkers, MWCNTs composites, and tested solvent ratios .

Elevated temperatures (120 and 160 °C, autoclave) and the addition of 3–10 mol % of different cross-linkers had insignificant effects. In the case of the THAQ and glutaraldehyde cross-linkers, detrimental effects on the electrochemical performance could be observed.

On the contrary, the addition of 10 wt % MWCNTs and increasing the  ratio showed significant improvement in electrochemical performance with respect to the initial polymers. The removal of glacial acetic acid from the solvent mixture caused an increase in the solubility of the monomer and its polymer counterpart, allowing polymerization to proceed for longer periods of time. Different from the earlier procedure, the reaction mixture would now solidify due to the polymer crushing out from the solution (see Section SI-II-2). In attempt to further improve the cycling performance, 10 wt % MWCNTs composites were synthesized using the optimized synthesis because the literature provides evidence that addition of MWCNTs improves structural stability and charge transfer kinetics.^44^

**Figure 3:**
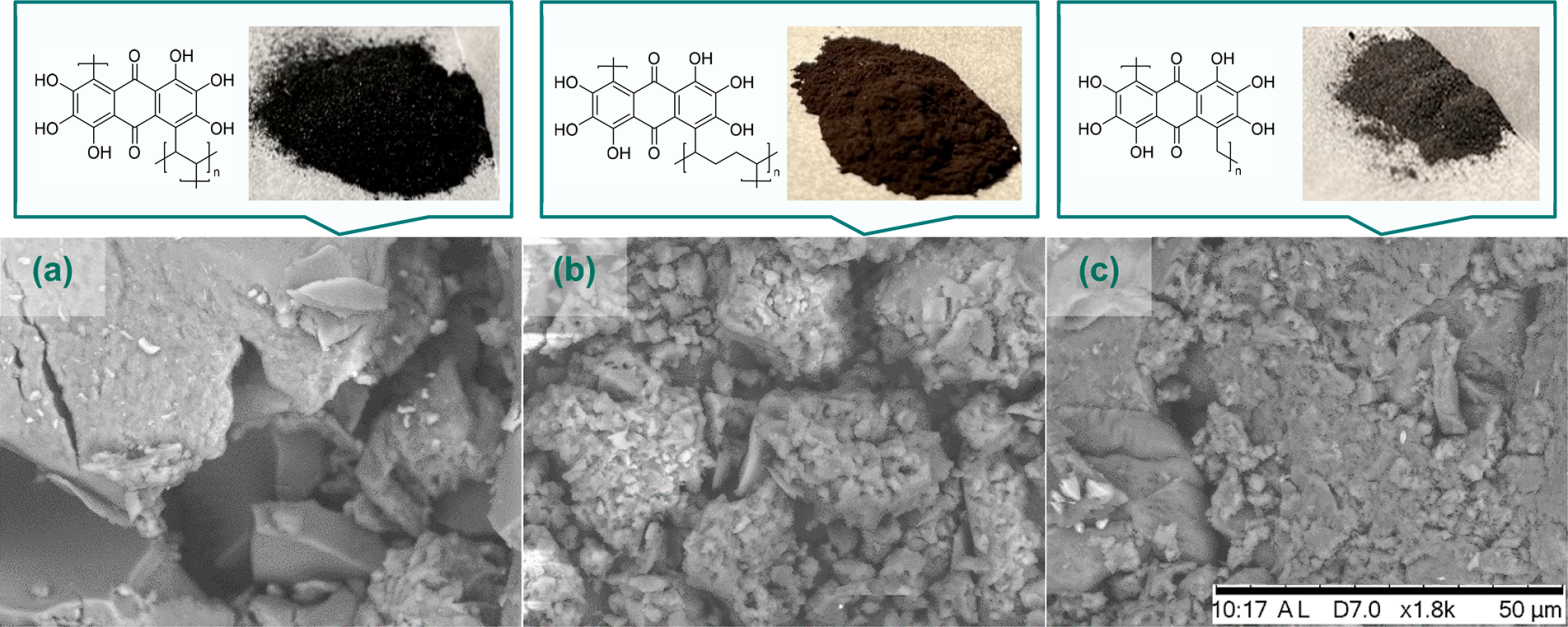
(a, b, c) SEM images of poly(HHAQ–glyoxal), poly(HHAQ–glutaraldehyde), and poly(HHAQ–formaldehyde), respectively.

As a final synthetical strategy, two alkyldialdehyde linkers, glyoxal and glutaraldehyde, were used as cross-linker (see Scheme 2a). Motivation for this was the introduction of (1) rigidity (glyoxal) and (2) improved accessibility of the redox-active sites, possibly increasing material utilization (glutaraldehyde). The SEM images of poly(HHAQ–glyoxal) and poly(HHAQ–glutaraldehyde) in Figure 3a,b confirmed the successful incorporation of the desired properties, where poly(HHAQ–glyoxal) appears to be more dense and poly(HHAQ–glutaraldehyde) more porous with respect to poly(HHAQ–formaldehyde) (Figure 3c; for more SEM images, see Section SI-II-3). However, unfortunately both materials experience more rapid fading with respect to polymeric counterparts incorporating formaldehyde as linker. A first attempt to explain this focuses on the relatively more flexible and longer alkyl chains allowing the electrodes to swell upon wetting with the 1.0 M LiPF_6_ EC:DEC (1:1, v/v) electrolyte. This affects the electrode–current collector contact and leads to rapid capacity fading. However, it is beyond the scope of this study to investigate this in detail.

**Figure 4:**
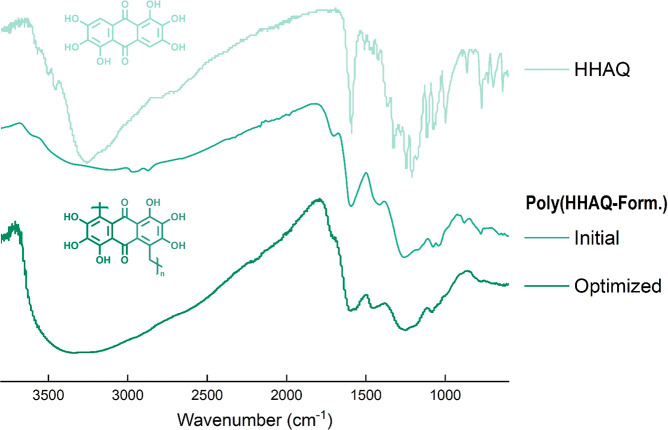
ATR-FTIR spectra of HHAQ and the initial and optimized poly(HHAQ–formaldehyde).

Figure 4 shows the ATR-FTIR spectra of HHAQ and of the initial and optimized poly(HHAQ–formaldehyde) ((poly(HHAQ–formaldehyde)-initial and (poly(HHAQ–formaldehyde)-optimized)). ATR-FTIR spectra of poly(THAQ–formaldehyde), poly(HHAQ–glyoxal), and poly(HHAQ–glutaraldehyde) are depicted in Section SI-I-3. Comparing the spectra of the monomer and polymers, several observations indicate the presence of the desired polymer structure. First, the peaks of poly(HHAQ–formaldehyde) appear much broader and with lower intensity with respect to those of the monomer. This is typical for a polymer. Second, the essential functional groups for the electrochemical performance (hydroxyl: 3255 cm^–1^ = O–H; anthraquinone: 1589 cm^–1^ = C=O) remain present upon polymerization. And finally, the incorporation of the formaldehyde becomes evident from the poly(HHAQ–formaldehyde) spectra, as the methylene bridging group and aldehyde end-group (≈2900 cm^–1^ = C–H and 1691 cm^–1^ = C=O, respectively) appear only in the polymer spectra. Similar trends can be observed when the ATR-FTIR spectra of THAQ and poly(THAQ-formaldehyde) are compared in Section SI-I-3.

### Electrochemical Characterization of PHAQ Polymers

**Figure 5:**
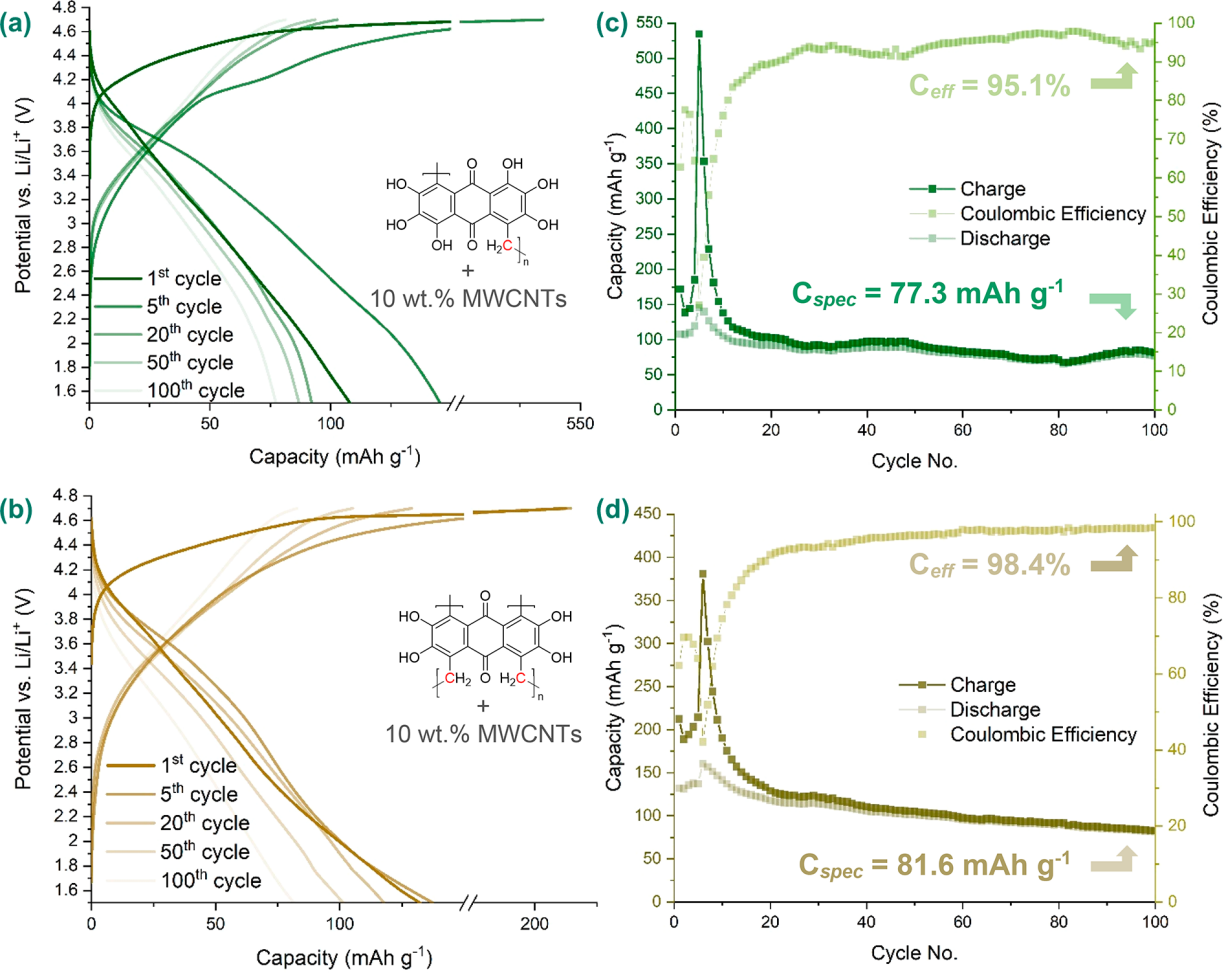
1st, 5th, 20th, 50th, and 100th galvanostatic charge–discharge cycle (C-rate = 1.0C) of (a) poly(HHAQ–formaldehyde)-10 wt %MWCNTs and (c) poly(THAQ–formaldehyde)-10 wt % MWCNTs composites in Li half-cells, using [Act. Mat.:C_65_:SEBS] = [40:50:10] electrode formulations and 1.0 M LiPF_6_ in EC:DEC (1:1, v/v) as electrolyte. (b) and (d) depict the charge and discharge capacity as well as the Coulombic efficiency (*C*_eff_) over 100 cycles for poly(HHAQ–formaldehyde)-10 wt %MWCNTs and poly(THAQ–formaldehyde)-10 wt %MWCNTs, respectively.

The cycling performance of all synthesized polymers was assessed as cathode material in lithium half-cells using 1.0 M LiPF_6_ in EC:DEC (1:1, v/v) as electrolyte. Figure 5 shows the galvanostatic cycling of poly(HHAQ–formaldehyde)-10 wt % MWCNTs and poly(THAQ–formaldehyde)-10 wt % MWCNTs, which were synthesized with the optimized polymerization procedure. This figure includes the 1st, 5th, 20th, 50th, and 100th voltage profiles (Figure 5a,c) and the charge/discharge capacity (*C*_spec_) as well as the Coulombic efficiency (*C*_eff_) (Figure 5b,d). Likewise, Section SI-II-4 contains similar cycling for poly(HHAQ–glutaraldehyde), poly(HHAQ–glyoxal), and poly(HHAQ–formaldehyde)-initial as well as the CVs of poly(HHAQ–formaldehyde) on a glassy carbon electrode.

The results shown in Figure 5 exhibit the positive effect of polymerization on the cycling stability of the material because discharge capacities of 77.3 mAh g^–1^ (53%) and 81.6 mAh g^–1^ (51%) were retained after 100 cycles at 1.0C for respectively the poly(HHAQ–formaldehyde)- and poly(THAQ–formaldehyde)-10 wt % MWCNT composites. Note that the contribution of carbon was calculated to be 24.06 mAh g^–1^ by cycling blank electrodes consistent with [C_65_:SEBS] = [80:20].

Comparing these and the highest obtained discharge capacities (145.4 and 160.45 mAh g^–1^ for the poly(HHAQ–formaldehyde)- and poly(THAQ–formaldehyde)-10 wt % MWCNT composites, respectively) with the maximum theoretical capacities shown in Figure 1 and Section SI-II-4, it becomes clear that the capacities are not addressed fully. Several different factors might explain the lower utilization of the active material. First, the electronic conduction has a significant effect on the achievable capacity. The former is dependent on the type of binder and the quality of contact between the active material, conductive carbon, and the Al current collector. Second, the electrolyte is known to have a huge influence on the attainable capacity and thorough electrolyte screening is an important facet in addressing the maximum amount of active material during battery operation.^38^ In the case of the PHAQs, 1.0 M LiPF_6_ in EC:DEC (1:1, v/v) was found to perform best with the high-voltage polyphenolic subgroup after a brief electrolyte screening. However, possibly other electrolytes might be a better fit for the anthraquinone redox couple.^38^ Finally, bulk polymer chain aggregates in the electrode (e.g., due to π–π stacking) also limit the material utilization because the electrolyte and conductive additive only interact with the redox moieties on the surface. Because the polymer is nonconductive, this can contribute to the low material utilization.

Although comparing Figure 2b with Figure 5a shows that the capacity retention improved drastically with respect to that of the monomers, Figures 5c and 5d also show a strong increase in capacity in cycles 5 and 6, respectively, followed by rapid capacity fade in the consecutive 10 cycles. The latter could be initiated by the volume expansion of the polymeric material upon contact with the electrolyte, resulting in contact loss between the Al current collector and the redox polymer. This hypothesis is plausible because upon oxidation of the polyphenolic moiety, the monomers started to dissolve into the electrolyte (see Section SI-II-4). Even though the polymer solubility is reduced considerably after polymerization, the polymer–solvent interactions remain similar to the monomer–solvent interaction, causing the material to swell instead of dissolve. This possibly deteriorates the contact between active material and the current collector. The latter becomes evident upon opening a lithium half-cell after cycling, as Section SI-II-4 shows an image of a poly(HHAQ–formaldehyde)-10 wt % MWCNT composite electrode obtained by opening the coin cell after 100 cycles.

Regardless of the capacity fading during the activation phase, Figure 5a,b demonstrates that the high-voltage discharge plateau starting at 4.0 V (vs Li/Li^+^) remains present over 100 cycles, making both the poly(HHAQ–formaldehyde)- and poly(THAQ–formaldehyde)-10 wt % MWCNT composites interesting as OEMs for high-energy LIBs. In contrast to Figure 2b, the discharge plateaus of the polyphenolic and anthraquinone redox centers are hard to distinguish in Figure 5a,b. The polymerization makes the redox potentials anisotropic, causing charge and discharge potentials of both the redox moieties to slightly alter for each repeating unit. Besides anisotropic effects, the binder can affect the shape of the discharge curve. The SEBS binder is known to be a strong nonconductive binder. Despite the necessity to use a strong binder (PVDF and carboxymethoxy–cellulose provided brittle and defoliating electrodes), the use of SEBS can limit the ionic conductivity inside the electrode. This generates resistance, which in turn affects the activation polarization, causing the discharge curve to slope more. Moreover, polymer chain aggregation in the electrode (e.g., due to π–π stacking) also contributes to increase resistance for electrolyte electrode percolation. All the aforementioned factors could contribute to the sloping nature of the discharge curve for the polyhydroxyanthraquinone polymers, where the discharge potential at half-capacity is 3.0–3.1 and 2.8–2.9 V (vs Li/Li^+^) for poly(HHAQ–formaldehyde)-10 wt % MWCNT and poly(THAQ–formaldehyde)-10 wt % MWCNT, respectively. Likewise, these factors could explain the appearance of the voltammograms of poly(HHAQ–formaldehyde) in Section SI-II-4 because the oxidation of the AQ and polyphenolic region seems to overlap and the polyphenolic reduction appears to happen over an increased voltage range. In Section SI-II-4 the d*Q*/d*V* plots of poly(HHAQ–formaldehyde)-10 wt % MWCNT and poly(THAQ–formaldehyde)-10 wt % MWCNT are shown.

Initial high capacities were also obtained for the poly(HHAQ–formaldehyde)-initial polymer and its composite. The latter even exhibited the highest retained capacity at 1.0C: 95.4 mAh g^–1^ after 100 cycles, despite having a relatively high fading compared to the 10 wt % MWCNTs poly(HHAQ–formaldehyde)-optimized composite (69% capacity loss after 100 cycles). At C-rates of 15.0C, the capacity retention was best with 95.4 and 106 mAh g^–1^ after 100 cycles for the MWCNTs composite and the regular polymer, respectively. Moreover, cycling at high C-rates was only possible for the initial polymers because the polymers obtained from the optimized polymerization displayed poor capacities at C-rates as low as 5.0C already. Finally, while poly(HHAQ–formaldehyde)-initial showed reasonable capacities, they failed to display the desired high-voltage discharge plateau. Only at 15.0C did the latter was demonstrated during the first 5–20 cycles. Note that poly(HHAQ–formaldehyde)-initial polymer cycled at 15.0C exhibits an activation phase during the first 40 cycles, where lower capacities are obtained due to an incomplete wetting process of the electrode.

## Conclusion

A family of bio-based PHAQs was synthesized to offer a high-voltage cathode material for batteries. Every PHAQ molecule was tested using cyclic voltammetry and lithium half-cell cycling and displayed a high-voltage discharge potential between 4.1 and 3.8 V (vs Li/Li^+^) corresponding to the polyphenolic subgroup. Additionally, all materials exhibited an anthraquinone discharge between 2.2 and 2.8 V (vs Li/Li^+^). For these PHAQs initial charge capacities of up to 383.75 mAh g^–1^ were obtained. Furthermore, the influence of the degree of hydroxylation on the redox potential was concisely investigated both empirically and *in silico*. Moreover, the reduction potentials obtained via CV deviated minimally from those obtained *in silico*.

To counteract the rapid fading caused by dissolution in the electrolyte, a facile polymerization method was employed to synthesize PHAQ–aldehyde polymers. Here the polymerization of HHAQ served as a model reaction, and several aldehyde linkers (glutaraldehyde, glyoxal, and formaldehyde) were tested. The resulting polymers were investigated as cathode materials in lithium metal batteries. PHAQ polymer composites synthesized using formaldehyde as linker and 10 wt % MWCNTs, namely poly(THAQ–formaldehyde)-10 wt % MWCNTs and poly(HHAQ–formaldehyde)-10 wt % MWCNTs, exhibited the best cycling performance, displaying a high-voltage discharge starting at 4.0 V (vs Li/Li^+^) and retaining 81.6 and 77.3 mAh g^–1^ and average discharge potentials of 2.8–2.9 and 3.0–3.1 V (vs Li/Li^+^), respectively, after 100 cycles. Therefore, this work displays the potential of PHAQs and their polymers as high-voltage, bio-based, organic cathode materials for high-power batteries.

## Supporting Information Available

SI-I: Experimental Section; SI-I-1: Materials; SI-I-2: Methods; SI-I-3: Synthesis; SI-I-3-1: Monomers Syntheses; SI-I-3-2: Polymers Syntheses; SI-II: Results and Discussion; SI-II-1: Anticipated Reaction Mechanism Polymerization HHAQ; SI-II-2: Images Solidified Polymerization Mixture; SI-II-3: SEM Images; SI-II-3-1: Poly[HHAQ-*alt*-formaldehyde]-10 wt % MWCNT composite; SI-II-3-2: Poly[THAQ-*alt*-formaldehyde]-10 wt % MWCNT composite; SI-II-4: Electrochemistry; SI-II-4-1: Electrochemical Mechanisms HHAQ; SIII-42: Electrochemical Mechanisms OHAQ; SI-II-4-3: Cyclic Voltammograms THAQ in 1.0 M LiPF_6_ in EC:DEC (1:1, v/v); SI-II-4-4: Cyclic Voltammograms HHAQ in 1.0 M LiPF_6_ in EC:DEC (1:1, v/v); SI-II-4-5: Cyclic Voltammograms OHAQ in 1.0 M LiPF_6_ in EC:DEC (1:1, v/v); SI-II-4-6: Galvanostatic cycling THAQ in Lithium half-cell; SI-II-4-6-A: Electrolyte: 0.3 M LiTFSI/[PY13][TFSI] ionic liquid; SI-II-4-7: Galvanostatic cycling HHAQ in Lithium half-cell; SI-II-4-7-A: Electrolyte: 0.3 M LiTFSI/[PY13][TFSI] ionic liquid; SI-II-4-8: Galvanostatic cycling OHAQ in Lithium half-cell; SI-II-4-8-A: Electrolyte: 0.3 M LiTFSI/[PY13][TFSI] ionic liquid; SI-II-4-9: Galvanostatic cycling poly[HHAQ-*alt*-glyoxal] in Lithium half-cell; SI-II-4-10: Galvanostatic cycling poly[HHAQ-*alt*-glutaraldehyde] in Lithium half-cell; SI-II-4-11: Galvanostatic cycling Low *M*_w_ poly[HHAQ-*alt*-formaldehyde] in Lithium half-cell; SI-II-4-12: Cyclic Voltammogram of poly[HHAQ-*alt*-formaldehyde] in 1.0 M LiPF_6_ in EC:DEC (1:1, v/v): SI-II-4-13: Decomposed postcycling poly[HHAQ-*alt*-formaldehyde] electrode (PDF)

## Abbreviations

OEM: organic electrode material
PHAQs: polyhydroxyanthraquinones
OHAQ: 1,2,3,4,5,6,7,8-octahydroxyanthraquinone
HHAQ: 1,2,3,5,6,7-hexahydroxyanthraquinone
THAQ: 2,3,6,7-tetrahydroxyanthraquinone
AQ: anthraquinone
MWCNTs: multiwalled carbon nanotubes
LIBs: lithium-ion batteries
CV: cyclic voltammetry
NMR: nuclear magnetic resonance
ATR-FTIR: attenuated total reflection Fourier transform infrared
MALDI-TOF: matrix assisted laser desorption/ionization time-of-flight analyzer
PCM: polarizable continuum model
*C*_theo_: theoretical capacity
*C*_spec_: specific capacity
DFT: density functional theory
*E*_red_: reduction potential
*M*_w_: molecular weight.

## References

[ref1] Scrosati B. History of Lithium Batteries. J. Solid State Electrochem. 2011, 15 (7), 1623–1630. 10.1007/s10008-011-1386-8.

[ref2] Poizot P.; Gaubicher J.; Renault S.; Dubois L.; Liang Y.; Yao Y. Opportunities and Challenges for Organic Electrodes in Electrochemical Energy Storage. Chem. Rev. 2020, 120 (14), 6490–6557. 10.1021/acs.chemrev.9b00482. 32207919

[ref3] Lu Y.; Chen J. Prospects of Organic Electrode Materials for Practical Lithium Batteries. Nat. Rev. Chem. 2020, 4 (3), 127–142. 10.1038/s41570-020-0160-9. 37128020

[ref4] Armand M.; Tarascon J.-M. Building Better Batteries. Nature 2008, 451 (7179), 652–657. 10.1038/451652a. 18256660

[ref5] Friebe C.; Lex-Balducci A.; Schubert U. S. Sustainable Energy Storage: Recent Trends and Developments toward Fully Organic Batteries. ChemSusChem 2019, 12 (18), 4093–4115. 10.1002/cssc.201901545. 31297974 PMC6790600

[ref6] Zhang S.; Ericsson N.; Hansson P.-A.; Sjödin M.; Nordberg Å. Life Cycle Assessment of an All-Organic Battery: Hotspots and Opportunities for Improvement. J. Clean. Prod. 2022, 337, 130454 10.1016/j.jclepro.2022.130454.

[ref7] Gallastegui A.; Minudri D.; Casado N.; Goujon N.; Ruipérez F.; Patil N.; Detrembleur C.; Marcilla R.; Mecerreyes D. Proton Trap Effect on Catechol–Pyridine Redox Polymer Nanoparticles as Organic Electrodes for Lithium Batteries. Sustain. Energy Fuels 2020, 4 (8), 3934–3942. 10.1039/D0SE00531B.

[ref8] Patil N.; Aqil A.; Ouhib F.; Admassie S.; Inganäs O.; Jérôme C.; Detrembleur C. Bioinspired Redox-Active Catechol-Bearing Polymers as Ultrarobust Organic Cathodes for Lithium Storage. Adv. Mater. 2017, 29 (40), 1703373 10.1002/adma.201703373. 28869678

[ref9] Yu Z.; Huang L.; Sun Z.; Cai F.; Liang M.; Luo Z. Designing Anthraquinone-Based Conjugated Microporous Polymers with Dual-Ion Storage Behavior towards High-Performance Lithium-Organic Batteries. J. Power Sources 2022, 550, 232149 10.1016/j.jpowsour.2022.232149.

[ref10] Eftekhari A. On the Theoretical Capacity/Energy of Lithium Batteries and Their Counterparts. ACS Sustain. Chem. Eng. 2019, 7 (4), 3684–3687. 10.1021/acssuschemeng.7b04330.

[ref11] Wild A.; Strumpf M.; Häupler B.; Hager M. D.; Schubert U. S. All-Organic Battery Composed of Thianthrene- and TCAQ-Based Polymers. Adv. Energy Mater. 2017, 7 (5), 1601415 10.1002/aenm.201601415.

[ref12] Nakahara K.; Iwasa S.; Satoh M.; Morioka Y.; Iriyama J.; Suguro M.; Hasegawa E. Rechargeable Batteries with Organic Radical Cathodes. Chem. Phys. Lett. 2002, 359 (5), 351–354. 10.1016/S0009-2614(02)00705-4.

[ref13] Nishide H.; Iwasa S.; Pu Y.-J.; Suga T.; Nakahara K.; Satoh M. Organic Radical Battery: Nitroxide Polymers as a Cathode-Active Material. Electrochim. Acta 2004, 50 (2), 827–831. 10.1016/j.electacta.2004.02.052.

[ref14] Speer M. E.; Kolek M.; Jassoy J. J.; Heine J.; Winter M.; Bieker P. M.; Esser B. Thianthrene-Functionalized Polynorbornenes as High-Voltage Materials for Organic Cathode-Based Dual-Ion Batteries. Chem. Commun. 2015, 51 (83), 15261–15264. 10.1039/C5CC04932F. 26235336

[ref15] Zeng R.; Xing L.; Qiu Y.; Wang Y.; Huang W.; Li W.; Yang S. Polycarbonyl(Quinonyl) Organic Compounds as Cathode Materials for Sustainable Lithium Ion Batteries. Electrochim. Acta 2014, 146, 447–454. 10.1016/j.electacta.2014.09.082.

[ref16] Lee M.; Hong J.; Kim H.; Lim H.-D.; Cho S. B.; Kang K.; Park C. B. Organic Nanohybrids for Fast and Sustainable Energy Storage. Adv. Mater. 2014, 26 (16), 2558–2565. 10.1002/adma.201305005. 24488928

[ref17] Wu Z.; Liu Q.; Yang P.; Chen H.; Zhang Q.; Li S.; Tang Y.; Zhang S. Molecular and Morphological Engineering of Organic Electrode Materials for Electrochemical Energy Storage. Electrochem. Energy Rev. 2022, 5 (1), 26 10.1007/s41918-022-00152-8.

[ref18] Kim K. C.; Liu T.; Lee S. W.; Jang S. S. First-Principles Density Functional Theory Modeling of Li Binding: Thermodynamics and Redox Properties of Quinone Derivatives for Lithium-Ion Batteries. J. Am. Chem. Soc. 2016, 138 (7), 2374–2382. 10.1021/jacs.5b13279. 26824616

[ref19] Huang W.; Jia T.; Zhou G.; Chen S.; Hou Q.; Wang Y.; Luo S.; Shi G.; Xu B. A Triphenylamine-Based Polymer with Anthraquinone Side Chain as Cathode Material in Lithium Ion Batteries. Electrochim. Acta 2018, 283, 1284–1290. 10.1016/j.electacta.2018.07.062.

[ref20] Nokami T.; Matsuo T.; Inatomi Y.; Hojo N.; Tsukagoshi T.; Yoshizawa H.; Shimizu A.; Kuramoto H.; Komae K.; Tsuyama H.; Yoshida J. Polymer-Bound Pyrene-4,5,9,10-Tetraone for Fast-Charge and -Discharge Lithium-Ion Batteries with High Capacity. J. Am. Chem. Soc. 2012, 134 (48), 19694–19700. 10.1021/ja306663g. 23130634

[ref21] Robiquet Ueber Die Gallussäure. Ann. der Pharm. 1836, 19 (2), 204–210. 10.1002/jlac.18360190212.

[ref22] Billard J.; Luz Z.; Poupko R.; Zimmermann H. The Mesophases of Octa-Alkanoyloxy-9,10-Anthraquinone. Liq. Cryst. 1994, 16 (2), 333–342. 10.1080/02678299408029157.

[ref23] Boldt P. Ein Neues Chinonsystem: Derivate Des Amphi-Anthrachinons. Chem. Ber. 1967, 100 (4), 1270–1280. 10.1002/cber.19671000428.

[ref24] Becke A. D. Density-functional Thermochemistry. III. The Role of Exact Exchange. J. Chem. Phys. 1993, 98 (7), 5648–5652. 10.1063/1.464913.

[ref25] Perdew J. P. In Electronic Structure of Solids’ 91; Akademie Verlag: Berlin, 1991.

[ref26] Burke K.; Perdew J. P.; Wang Y. Electronic Density Functional Theory: Recent Progress and New Directions; Plenum: New York, 21998.

[ref27] Hehre W. J.; Ditchfield R.; Pople J. A. Self—Consistent Molecular Orbital Methods. XII. Further Extensions of Gaussian—Type Basis Sets for Use in Molecular Orbital Studies of Organic Molecules. J. Chem. Phys. 1972, 56 (5), 2257–2261. 10.1063/1.1677527.

[ref28] Woon D. E.; Dunning T. H. Gaussian Basis Sets for Use in Correlated Molecular Calculations. III. The Atoms Aluminum through Argon. J. Chem. Phys. 1993, 98 (2), 1358–1371. 10.1063/1.464303.

[ref29] Barone V.; Cossi M.; Tomasi J. A New Definition of Cavities for the Computation of Solvation Free Energies by the Polarizable Continuum Model. J. Chem. Phys. 1997, 107 (8), 3210–3221. 10.1063/1.474671.

[ref30] Cancès E.; Mennucci B.; Tomasi J. A New Integral Equation Formalism for the Polarizable Continuum Model: Theoretical Background and Applications to Isotropic and Anisotropic Dielectrics. J. Chem. Phys. 1997, 107 (8), 3032–3041. 10.1063/1.474659.

[ref31] Cossi M.; Barone V.; Cammi R.; Tomasi J. Ab Initio Study of Solvated Molecules: A New Implementation of the Polarizable Continuum Model. Chem. Phys. Lett. 1996, 255 (4), 327–335. 10.1016/0009-2614(96)00349-1.

[ref32] Barone V.; Cossi M.; Tomasi J. Geometry Optimization of Molecular Structures in Solution by the Polarizable Continuum Model. J. Comput. Chem. 1998, 19 (4), 404–417. 10.1002/(SICI)1096-987X(199803)19:4<404::AID-JCC3>3.0.CO;2-W.

[ref33] Frisch M. J.; Trucks G. W.; Schlegel H. B.; Scuseria G. E.; Robb M. A.; Cheeseman J. R.; Scalmani G.; Barone V.; Petersson G. A.; Nakatsuji H.; Li X.; Caricato M.; Marenich A. V.; Bloino J.; Janesko B. G.; Gomperts R.; Mennucci B.; Hratchian H. P.; Ortiz J. V.; Izmaylov A. F.; Sonnenberg J. L.; Williams-Young D.; Ding F.; Lipparini F.; Egidi F.; Goings J.; Peng B.; Petrone A.; Henderson T.; Ranasinghe D.; Zakrzewski V. G.; Gao J.; Rega N.; Zheng G.; Liang W.; Hada M.; Ehara M.; Toyota K.; Fukuda R.; Hasegawa J.; Ishida M.; Nakajima T.; Honda Y.; Kitao O.; Nakai H.; Vreven T.; Throssell K.; Montgomery J. A. Jr.; Peralta J. E.; Ogliaro F.; Bearpark M. J.; Heyd J. J.; Brothers E. N.; Kudin K. N.; Staroverov V. N.; Keith T. A.; Kobayashi R.; Normand J.; Raghavachari K.; Rendell A. P.; Burant J. C.; Iyengar S. S.; Tomasi J.; Cossi M.; Millam J. M.; Klene M.; Adamo C.; Cammi R.; Ochterski J. W.; Martin R. L.; Morokuma K.; Farkas O.; Foresman J. B.; Fox D. J. Gaussian 16, Revision C.01, 2016; Gaussian 16, Revision A.03; Gaussian, Inc.: Wallingford, CT.

[ref34] Karamać M.; Kosińska A.; Pegg R. B. Content of Gallic Acid in Selected Plant Extracts. Polish J. Food Nutr. Sci. 2006, 56 (1), 55–58.

[ref35] Akhtar T. A.; Pichersky E. Veratrole Biosynthesis in White Campion. Plant Physiol. 2013, 162 (1), 52–62. 10.1104/pp.113.214346. 23547102 PMC3641228

[ref36] Lin K.; Chen Q.; Gerhardt M. R.; Tong L.; Kim S. B.; Eisenach L.; Valle A. W.; Hardee D.; Gordon R. G.; Aziz M. J.; Marshak M. P. Alkaline Quinone Flow Battery. Science. 2015, 349 (6255), 1529–1532. 10.1126/science.aab3033. 26404834

[ref37] Han X.; Lin G.; Zhang Q.; Yang Y. Rheological Phase Reaction Synthesis and Electrochemical Performance of Rufigallol Anode for Lithium Ion Batteries. RSC Adv. 2018, 8 (34), 19272–19277. 10.1039/C8RA02610F. 35539661 PMC9080710

[ref38] Phadke S.; Cao M.; Anouti M. Approaches to Electrolyte Solvent Selection for Poly-Anthraquinone Sulfide Organic Electrode Material. ChemSusChem 2018, 11 (5), 965–974. 10.1002/cssc.201701962. 29205911

[ref39] Goujon N.; Casado N.; Patil N.; Marcilla R.; Mecerreyes D. Organic Batteries Based on Just Redox Polymers. Prog. Polym. Sci. 2021, 122, 101449 10.1016/j.progpolymsci.2021.101449.

[ref40] Rehse K.; Kawerau H.-G. Untersuchungen Über Den Mechanismus Der Reaktion von Aromaten Mit Marquis Reagens. Arch. Pharm. (Weinheim) 1974, 307 (12), 934–942. 10.1002/ardp.19743071208. 4451450

[ref41] Zieger M. M.; Pop-Georgievski O.; de los Santos Pereira A.; Verveniotis E.; Preuss C. M.; Zorn M.; Reck B.; Goldmann A. S.; Rodriguez-Emmenegger C.; Barner-Kowollik C. Ultrathin Monomolecular Films and Robust Assemblies Based on Cyclic Catechols. Langmuir 2017, 33 (3), 670–679. 10.1021/acs.langmuir.6b03419. 28001408

[ref42] Hu X.; Li Z.; Yang Z.; Zhu F.; Zhao W.; Duan G.; Li Y. Fabrication of Functional Polycatechol Nanoparticles. ACS Macro Lett. 2022, 11 (2), 251–256. 10.1021/acsmacrolett.1c00729. 35574777

[ref43] Pirnat K.; Mali G.; Gaberscek M.; Dominko R. Quinone-Formaldehyde Polymer as an Active Material in Li-Ion Batteries. J. Power Sources 2016, 315, 169–178. 10.1016/j.jpowsour.2016.03.010.

[ref44] Wu H.; Wang K.; Meng Y.; Lu K.; Wei Z. An Organic Cathode Material Based on a Polyimide/CNT Nanocomposite for Lithium Ion Batteries. J. Mater. Chem. A 2013, 1 (21), 6366–6372. 10.1039/c3ta10473g.

