# Supplementary material for: Bio-Based Polyhydroxyanthraquinones as High-Voltage Organic Electrode Materials for Batteries
Source: ACS Appl Polym Mater. 2023 Oct 10;5(11):9128–37. doi: 10.1021/acsapm.3c01616 (PMC10644323; doi:10.1021/acsapm.3c01616)
Supplement: Supplementary file 1 — ap3c01616_si_001.pdf [file ap3c01616_si_001.pdf]

Supporting Information

Bio-based Polyhydroxyanthraquinones as High-Voltage Organic Electrode Materials for Batteries

Tijs Lap [a], Nicolas Goujon [ab], Daniele Mantione [ac], ~~Fernando Ruipérez~~ Fernando Ruiperez Cillan [ad], David Mecerreyes [ac]*

[a] POLYMAT University of the Basque Country UPV/EHU, Joxe Mari Korta Center, 20018, Donostia-San Sebastiań, Spain

[b] Centre for Cooperative Research on Alternative Energies (CIC energiGUNE), Basque Research and Technology Alliance (BRTA), Alava Technology Park, Albert Einstein 48, 01510, Vitoria-Gasteiz, Spain.

[c] Ikerbasque, Basque Foundation for Science, 48013 Bilbao, Spain

[d] Physical Chemistry Department, Faculty of Pharmacy, University of the Basque Country UPV/EHU, 01006 Vitoria-Gasteiz, Spain

Contents

SI-I Experimental Section 3

SI-I-1 Materials 3

SI-I-2 Methods 3

SI-I-3 Synthesis 5

SI-I-3-1 Monomers Syntheses 5

SI-I-3-2 Polymers Syntheses 14

SI-II Results and Discussion 20

SI-II-1 Anticipated Reaction Mechanism Polymerization HHAQ 20

SI-II-2 Images Solidified Polymerization Mixture 21

SI-II-3 SEM Images 22

SI-II-3-1 Poly(HHAQ-formaldehyde)-10 wt.% MWCNT composite 22

SI-II-3-2 Poly(THAQ-formaldehyde)-10 wt.% MWCNT composite | Solvent: Conc. H_2SO_4 24

SI-II-4 Electrochemistry 26

SI-II-4-1 Electrochemical Mechanisms HHAQ 26

SI-II-4-2 Electrochemical Mechanisms OHAQ 26

SI-II-4-3 Cyclic Voltammograms THAQ in 1.0M $LiPF_6$ in EC:DEC (1:1, v:v) 27

SI-II-4-4 Cyclic Voltammograms HHAQ in 1.0M $LiPF_6$ in EC:DEC (1:1, v:v) 29

SI-II-4-5 Cyclic Voltammograms OHAQ in 1.0M $LiPF_6$ in EC:DEC (1:1, v:v) 31

SI-II-4-6 Galvanostatic cycling THAQ in lithium metal battery 33

SI-II-4-6-A Electrolyte: 0.3 M LiTFSI/[PY13][TFSI] ionic liquid 33

SI-II-4-7 Galvanostatic cycling HHAQ in lithium metal battery 34

SI-II-4-7-A Electrolyte: 0.3 M LiTFSI/[PY13][TFSI] ionic liquid 34

SI-II-4-8 Galvanostatic cycling OHAQ in lithium metal battery 35

SI-II-4-8-A Electrolyte: 0.3 M LiTFSI/[PY13][TFSI] ionic liquid 35

SI-II-4-9 Galvanostatic cycling poly(HHAQ-glyoxal) in lithium metal battery 35

SI-II-4-10 Galvanostatic cycling poly(HHAQ-glutaraldehyde) in lithium metal battery 36

SI-II-4-11 Galvanostatic cycling initial poly(HHAQ-formaldehyde) in lithium metal battery 37

SI-II-4-12 Cyclic Voltammogram of poly(HHAQ-formaldehyde) in 1.0M $LiPF_6$ in EC:DEC (1:1, v:v) 39

....40

SI-II-4-13 Decomposed post-cycling poly(HHAQ-formaldehyde) electrode 40

SI-II-4-14 dQ/dV plot of poly(HHAQ-formaldehyde)-10wt.%MWCNTs 41

SI-II-4-15 dQ/dV plot of poly(THAQ-formaldehyde)-10wt.%MWCNTs 41

References 42

SI-I Experimental Section

SI-I-1 Materials

Gallic acid (98%), sulfuric acid (95.0-98.0%, ACS reagent) and boric acid (99.6%, ACS reagent) were purchased from Thermo Fisher Scientific. Pyridine (certified AR for analysis), glacial acetic acid, methanol (certified ACS), chloroform (99.8+%, Certified AR for Analysis, Stabilised with Amylene), hydrobromic acid (48% aq.) and hydrochloric acid (37% aq.) were purchased from Fisher Chemicals. Formaldehyde solution (ACS reagent, 37 wt.% aq. , contains 10-15% Methanol as stabilizer), glutaraldehyde solution (50 wt.% aq.), glyoxal solution (40 wt.% aq.), sodium hydroxide (≥98%, pellets (anhydrous), reagent grade), Toluene, N-methylpyrrolidone (NMP 99.5%, anhydrous), Carboxymethyl Cellulose (CMC) and Polystyrene-block-poly(ethylene-ran-butylene)-block-polystyrene (SEBS, average Mw ~118,000 by GPC, contains >0.03% antioxidant as inhibitor) were purchased from Sigma Aldrich. Veratrole (99%), Mercury (II) oxide (red, 99%) and sodium dichromate dehydrate (98%) were purchased from Alfa Aesar. Absolute ethanol was purchased from Sharlab. Lithium bis(trifluoromethanesulfonyl)imide (LiTFSI) was purchased from Iolitec. Battery electrolyte (1.0M lithium hexafluorophophate in ethylene carbonate/diethyl carbonate, 1:1, v:v) and 1-Butyl-1-methylpyrrolidinium bis(trifluoromethanesulfonyl)imide ([PY13][TFSI], 99.9%) were purchased from Solvionic. Carbon coated aluminium foil (width 260 mm, thickness 20 μm), lithium metal (width 60 mm, thickness 100 μm), polyvinylidene difluoride (PVDF; Solef® 5130) and conductive carbon black super C65 (Timical) was purchased from Gelon energy Co. Limited. 0.3 M LiTFSI/[PY13][TFSI] ionic liquid solutions and SEBS stock solutions (15 wt.% in Toluene) were prepared overnight afore use. All other chemicals and solvents were used as received.

SI-I-2 Methods

NMR Spectroscopy ^{1}H NMR and ^{13}C NMR spectra were recorded with Bruker Avance DPX 300 or Bruker Avance 400 spectrometers. The NMR chemical shifts were reported as δ in parts per million (ppm) relative to the traces of non-deuterated solvent (e.g. δ = 2.50 ppm for DMSO-d_6 or δ = 7.26 for $CDCl_3$).

ATR-FTIR spectroscopy FTIR spectra were obtained using an FTIR spectrophotometer (Nicolet is20, Thermo Scientific Inc.) equipped with the ATR feature with a diamond crystal. Spectra were recorded between 4000 and 500 cm−1 with a spectrum resolution of 4 cm^{-1}. All spectra were averaged over 16 scans.

Electrode formulation The polyhydroxyanthraquinone-based (either the *alt*-polymers, their 10wt.% MWCNTs composites or THAQ, HHAQ or OHAQ) electrodes were prepared using a doctor blade coating method onto a carbon coated aluminium current collector (Gelon energy Co. Limited, 11 μm thick). For this purpose, a slurry of THAQ, HHAQ or OHAQ (40 wt.%) was prepared with conductive carbon (Super C65, Timical, 40 wt.%) and binder (PVDF; Solef® 5130, 20 wt.%) in NMP using a speed mixer at 3000 RPM for 3 minutes after each solvent addition (SpeedMixerTM, DAC 300-100-SE). In case of the polymeric materials and their 10 wt.% composites, a slurry of 40 wt.% active material was prepared with conductive carbon (Super C65, Timical, 50 wt.% or in case of CMC-binder: 55 wt.%) and binder (SEBS, 15 wt.% stock solution, 10 wt.% or CMC: 5 wt.%) in 300 μL Toluene (demineralized water for CMC) using a speed mixer (SpeedMixerTM, DAC 300-100-SE) at 3000 RPM for 3x3 minutes with 30 seconds manual mixing in between spins. Prior to solvent mixing, all dried slurry components were mixed with a ball mill machined at 500 RPM for 5 minutes (FRITSCH, Planetary Micro Mill PULVERISETTE 7). All electrodes were dried under high vacuum at 50 °C for 24h, resulting in an active material loading ~2.0 mg·cm^{-2} and an electrode thickness of 97-112 nm.

Electrochemical characterization: Lithium metal batteries, based on cathodes of THAQ, HHAQ, OHAQ, or the *alt*-polymers or their MWCNTs composites, were assembled inside an

argon glovebox using a lithium electrode (Gelon Supporting information 4 energy Co. Limited, 11 µm thick) and a cathode electrode of a diameter of 11 mm. A glass microfiber filter (WhatmanTM, GF/B, 16 mm in diameter) soaked in either 240 µL 0.3 M LiTFSI/[PY13][TFSI] or 1.0M LiPF6 in EC:DEC (1:1, v:v) was used as separator. Cyclic voltammetry (CV) was performed in a three-electrode-cell using a glassy carbon working electrode and lithium metal strips as counter and reference electrode or a lithium metal coin cell. Galvanostatic measurements were performed using a multi-channel Potentiostat (Biologic, VMP3) or a battery cycler (Neware), respectively. The lithium metal coin cells were cycled directly upon assembly.

Computational calculations The reduction potentials have been calculated using the thermodynamic cycle shown in Figure S1, where the Gibbs free energy of the reduction half-reaction ($\Delta G^{RED}_{(solv)}$) consists of the free energy change in the gas phase ($\Delta G^{RED}_{(g)}$) and the solvation free energies (in methanol) of the oxidized ($\Delta G_{(solv)}(Ox)$) and the reduced ($\Delta G_{(solv)}(Red)$) species:

$$\Delta G^{RED}_{(solv)} = \Delta G^{RED}_{(g)} + \Delta G_{(solv)}(Red) - \Delta G_{(solv)}(Ox)$$

The relation between the Gibbs energy and the electrode potential (*E*) of a half-cell is:

$$E = \left(-\frac{\Delta G}{nF} + E_{SHE} \right) - E_{Li}$$

where *F* is the Faraday's constant (96485 J·mol^{-1}·V^{-1}) and *n* is the number of electrons transferred in the half reaction, with the subtraction of the reduction potential of the reference electrode. In this work, we have used the standard hydrogen electrode (E_{SHE} = -4.28 V) and the Li/Li$^+$ redox couple ($E_{Li/Li+}$ = -3.04 V). [1]

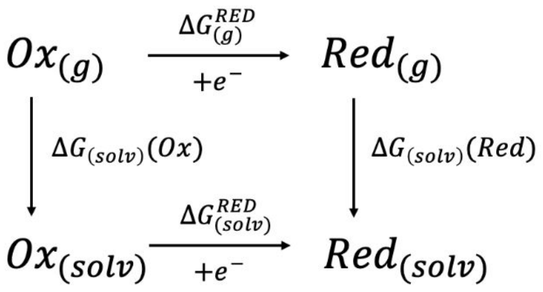

Figure S1. Thermodynamic cycle used in the calculation of the redox potentials.

Calculation theoretical capacity The theoretical capacity of the PHAQs and their polymer and polymer composite counterparts have been calculated following:

$$Q_m(C\,g^{-1}) = n \cdot \frac{F}{M}(mAh\,g^{-1})$$

Where Q_m is the theoretical capacity, n is the number of redox (electroactive) chemical species, F is the faradic constant and M is the molar mass. Based on initial experiments we assumed a 4 electron process: 2 electrons for the anthraquinone moiety and 2 electrons for the polyphenolic parts, were just one of the two phenolic parts per monomer unit would participate in the redox process.

SI-I-3 Synthesis

SI-I-3-1 Monomers Syntheses

Synthesis of 1,2,3,5,6,7-hexahydroxyanthraquinone (Rufigallol)

1,2,3,5,6,7-hexahydroxyanthraquinone was synthesized according to literature.[2] To a 100 mL round bottom flask equipped with a magnetic stirrer, 12 g (39.45 mmol) gallic acid and 24 mL conc. H_2SO_4 were added. The resulting solution was heated to 130 °C overnight in an oil bath. Subsequently, the mixture was left to cool down to room temperature and added to 500 mL demineralized water. The red-brown precipitate was vacuum filtered on a glass fritted filter and washes with demineralized water 3-4 times (50 mL) and dried in a vacuum oven at 100 °C overnight. *Yield: 92%. ^{1}H NMR (300 MHz, DMSO-d_6) δ 12.92 (s, 2H), 10.71 (s, 2H), 9.92 (s, 2H), 7.24 (s, 2H). ^{13}C NMR (75 MHz, DMSO-d_6) δ 185.87, 152.04, 151.28, 139.04, 124.40, 110.14, 108.51. ATR-FTIR (cm^{-1}): 3504, 3457, 3255, 1591, 1327, 1217, 1207, 1117, 1076, 999, 864, 771, 696, 638.*

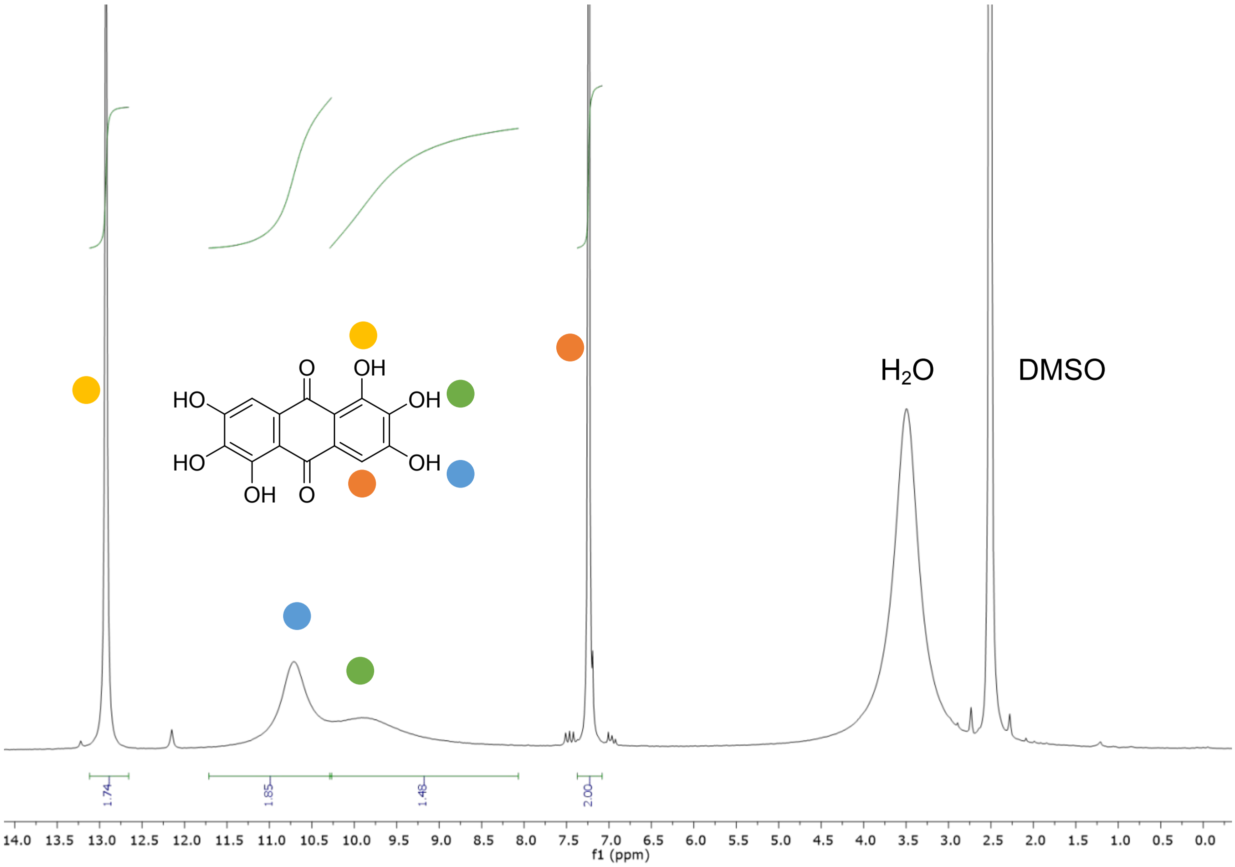

Figure S2. ^{1}H NMR spectrum of 1,2,3,5,6,7-Hexahydroxyanthraquinone in DMSO-d_6.

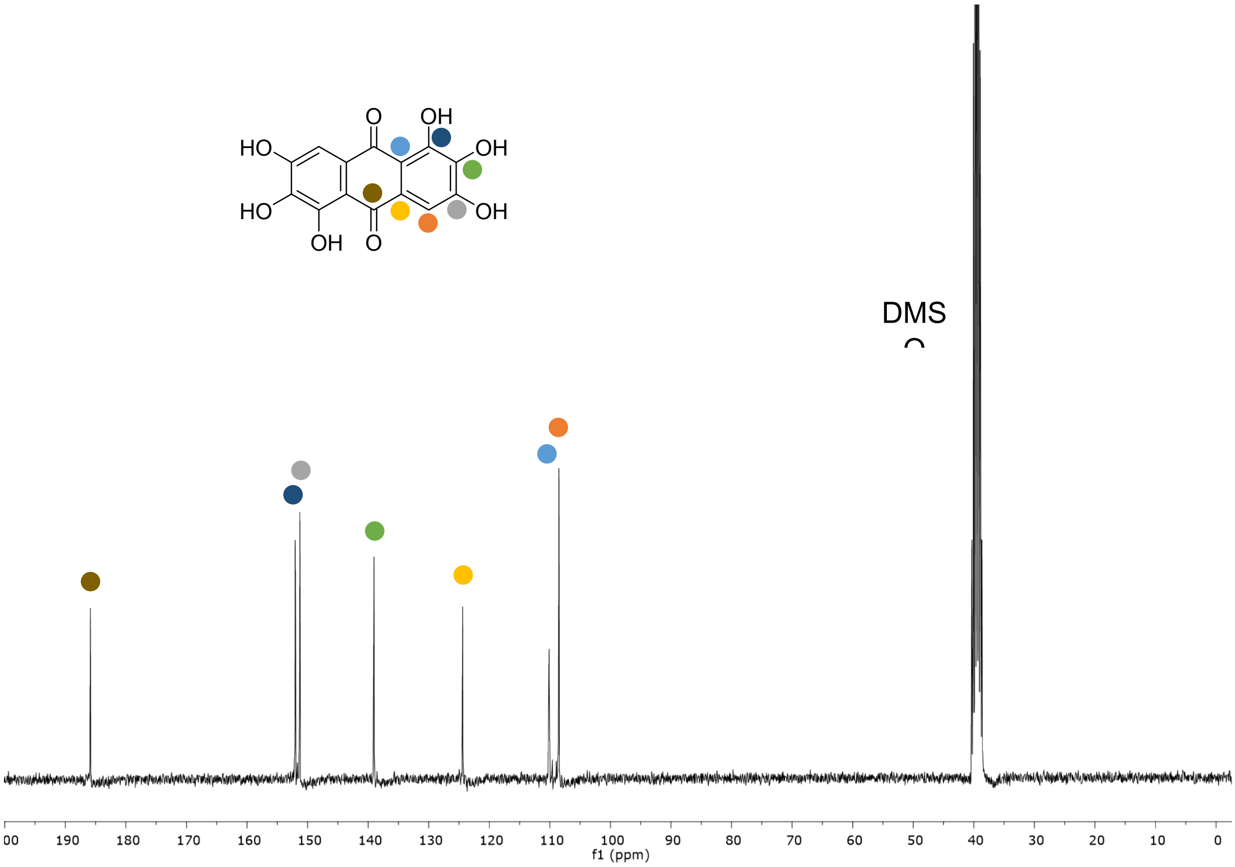

Figure S3. ^{13}C NMR spectrum of 1,2,3,5,6,7-Hexahydroxyanthraquinone in DMSO-d_6.

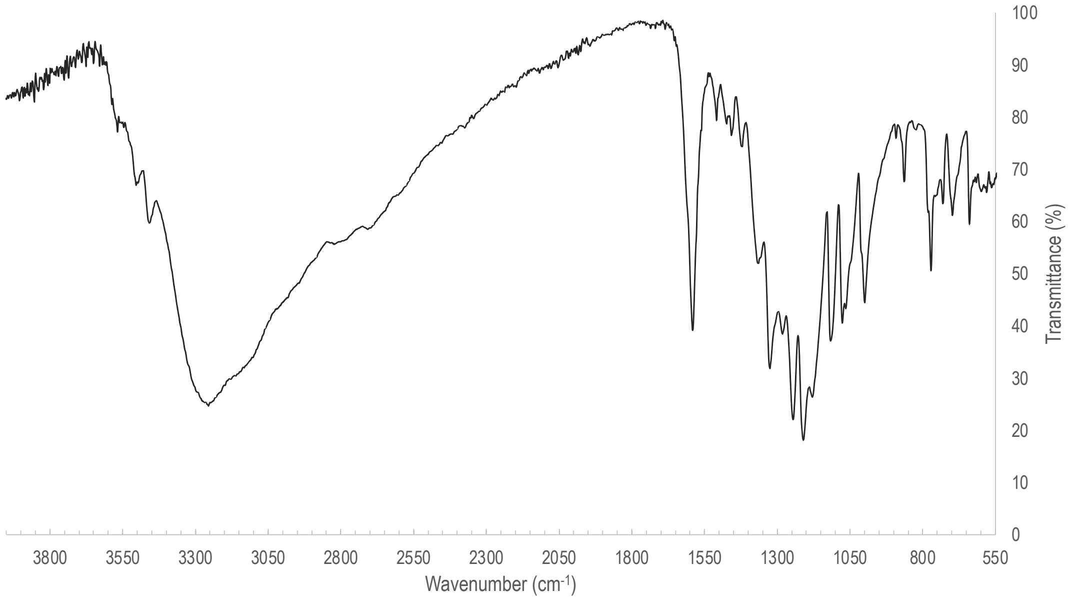

Figure S4. ATR-FTIR spectrum of 1,2,3,5,6,7-Hexahydroxyanthraquinone.

Synthesis of 1,2,3,4,5,6,7,8-octahydroxyanthraquinone

1,2,3,4,5,6,7,8-octahydroxyanthraquinone was synthesized according to literature.[2] To a 250 mL round bottom flask equipped with a magnetic stirrer, 2.6 g (8.52 mmol) 1,2,3,5,6,7-hexahydroxyanthraquinone, 43 mL conc. H_2SO_4 were added. While stiring, 3.45g (6.55 eq) boric acid and 60.5 mg (0.033 eq.) MgO were added over 10 minutes. The resulting mixture was lowered in a pre-heated oil bath of 250 °C for 45 minutes. The mixture was initially allowed to cool down to room temperature afore a final cooling step in an ice bath. Subsequently, the solution was poored over 172.8 g of ice, followed by boiling for 15 minutes. The cooled down solution was stored in the fridge overnight. The formed crystals were filtered, washed with demineralized H_2O and dried. Next, the product was dissolved in 100 mL boiling pyridine and the insoluble impurities were removed by filtration. The filtrate was mixed with 100 mL hot methanol followed by 43.2 mL hot demineralized water and allowed to cool down. Finally, the product was obtained via vacuum filtration and dried overnight at 150 °C under vacuum. *Yield: 34%. ^{13}C NMR (75 MHz, DMSO-d_6) δ 186.67, 149.30, 141.18, 105.36. ATR-FTIR (cm^{-1}): 3525-2775, 1573, 1436, 1234, 1197, 1078, 985, 800, 769.*

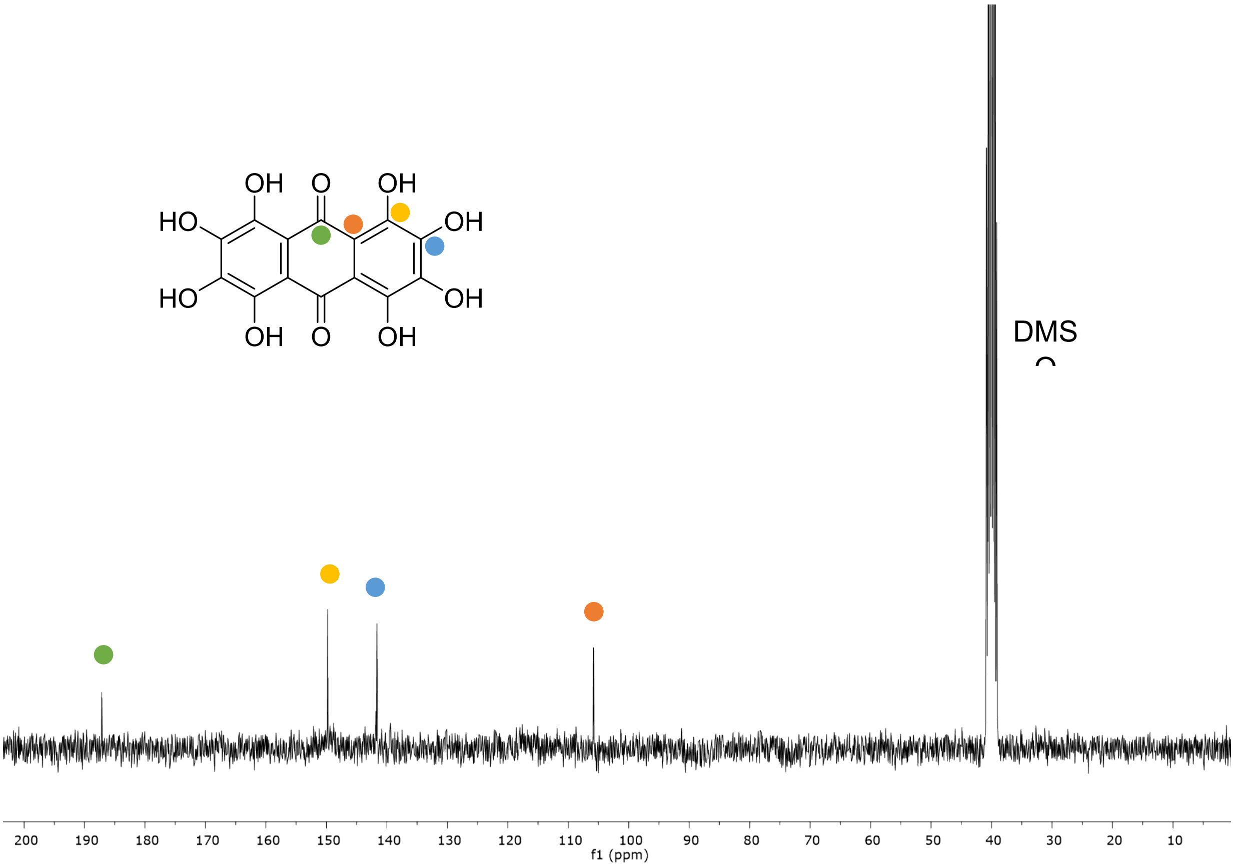

Figure S5. ^{13}C NMR spectrum of 1,2,3,4,5,6,7,8-Octahydroxyanthraquinone in DMSO-d_6.

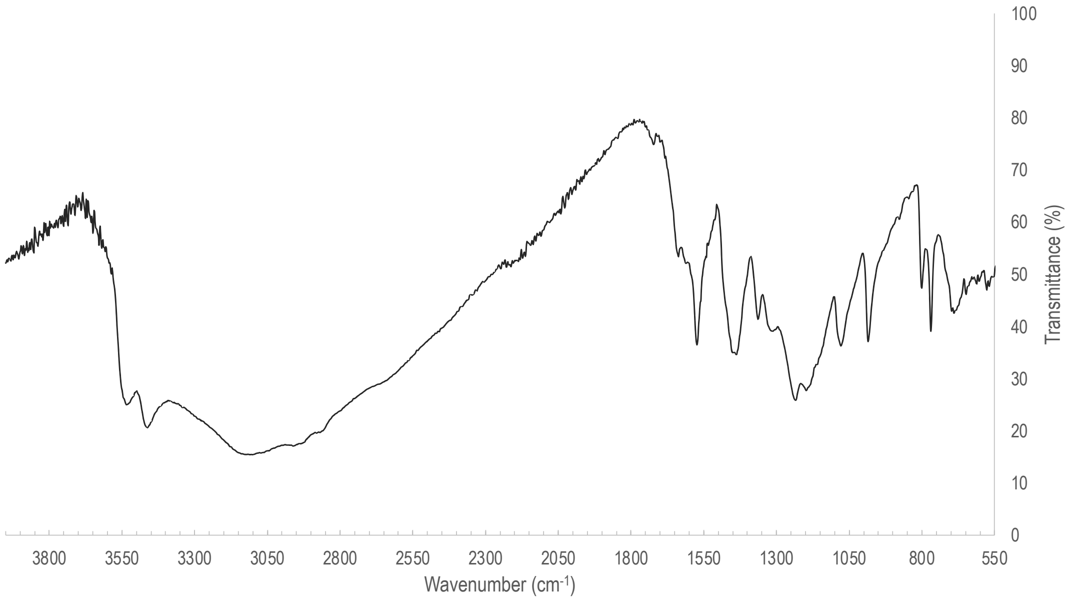

Figure S6. ATR-FTIR spectrum of 1,2,3,4,5,6,7,8-Octahydroxyanthraquinone.

Synthesis of 2,3,6,7-tetrahydroxyanthraquinone

2,3,6,7-tetrahydroxyanthraquinone was synthesized according to literature.[3]

Step 1: Dimerization Veratrole by double acetaldehyde linkage

To a 1000 mL round bottom flask equipped with a magnetic stirrer, 30 g (217.13 mmol) and 60 mL 72% H_2SO_4 were added and the resulting solution was cooled to 0 ^oC. Subsequently, 28.1 mL (2.3 eq.) acetaldehyde was added dropwise. The reaction mixture was allowed to reach room temperature slowly, while being stirred for 24 hours. Subsequently, 800 mL 96% ethanol was added and the precipitated solid was isolated by vacuum filtration. The product was dissolved in 1500 mL $CHCl_3$ and obtained after recrystallization overnight in the fridge. *Yield: 24%. ^1H NMR (300 MHz, Chloroform-d) δ 7.43 (s, 4H), 4.10 (s, 12H), 2.97 (s, 6H).*

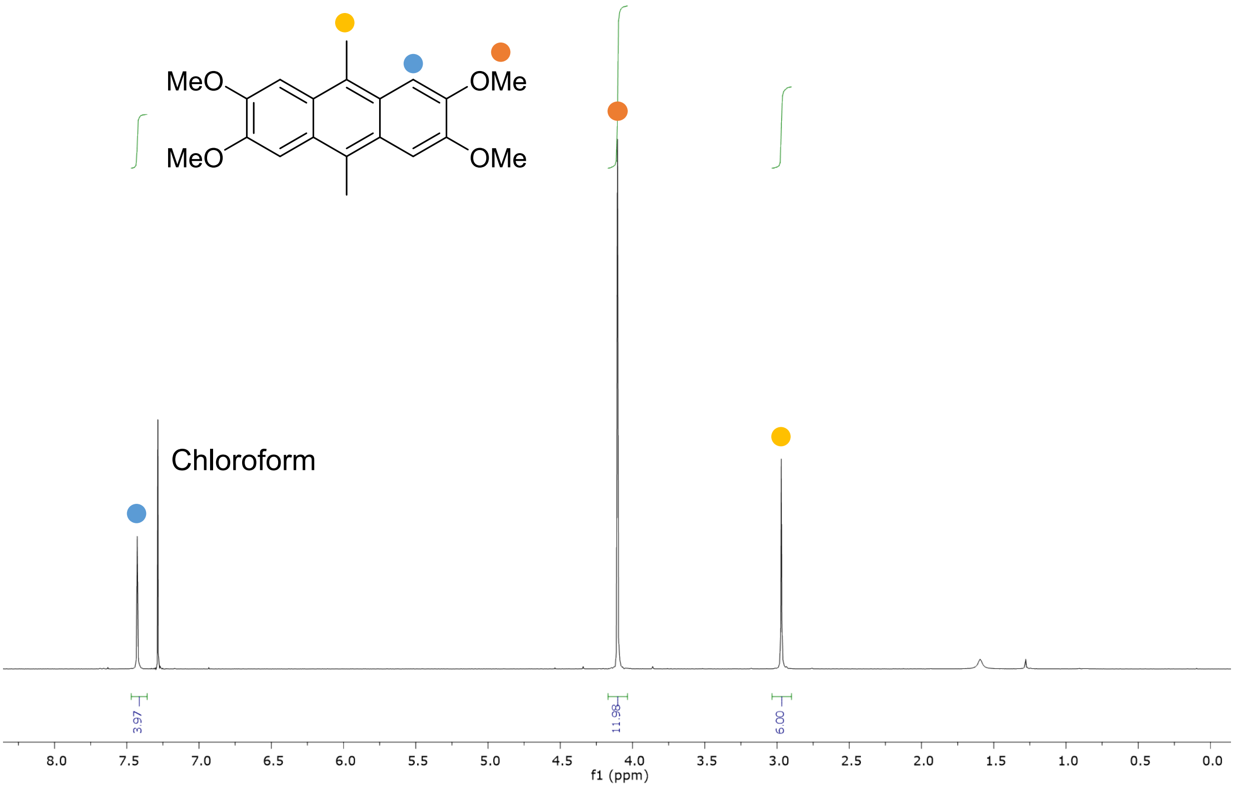

Figure S7. ^{1}H NMR spectrum of 2,3,6,7-Tetramethoxy-9,10-dimethylanthracen in $CDCl_3$.

Step 2: Oxidation of 2,3,6,7-tetramethoxy-9,10-dimethyl-9,10-dihydroanthracene

To a 1000 mL round bottom flask equipped with a magnetic stirrer, 7.9 g (24.2 mmol) 2,3,6,7-tetramethoxy-9,10-dimethyl-9,10-dihydroanthracene, 39.5 g $Na_2Cr_2O_7$ (5.47 eq) and 395 mL glacial acetic acid were added. Subsequently, the reaction mixture was refluxed for 1 hour, cooled down and stored in the fridge for 1 hour. The product was obtained by vacuum filtration, washed with demineralized water and dried overnight at 100 °C under vacuum. *Yield: 91%. 1H NMR (300 MHz, Chloroform-d) δ 7.71 (s, 4H), 4.09 (s, 12H).*

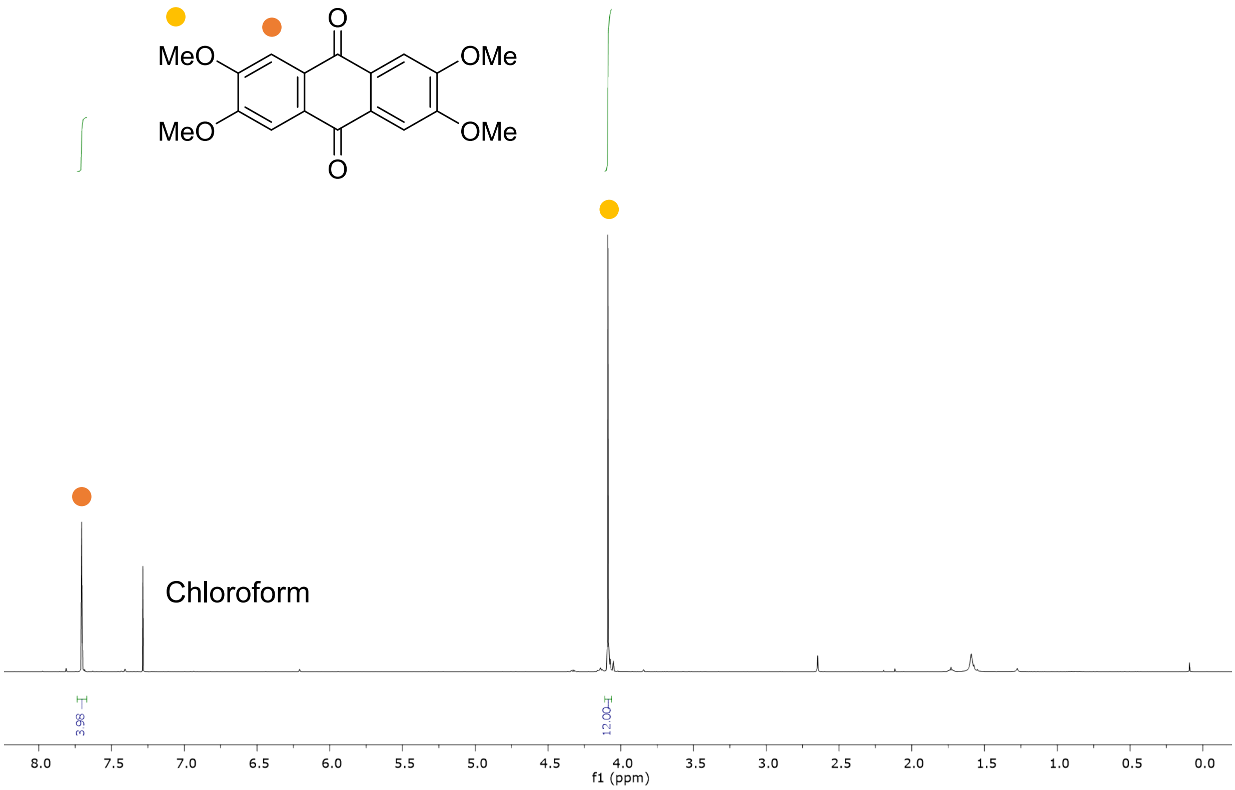

Figure S8. ^{1}H NMR spectrum of 2,3,6,7-tetramethoxyanthraquinone in $CDCl_3$.

Step 3: Methoxy-deprotection of 2,3,6,7-tetramethoxyanthraquinone

To a 100 mL round bottom flask equipped with a magnetic stirrer, 3.5 g (10.66 mmol) 2,3,6,7-tetramethoxyanthraquinone and 32 mL HBr (48% aq.) were added. The resulting mixture was refluxed for 3 days. After 24h an additional 9.6 mL HBr (48% aq.) was added. After 3 days, the reaction mixture was cooled down and the crude product was obtained upon vacuum filtration. The product was purified by recrystallization from pyridine/2N HCl (250 mL: 100 mL 37% HCl). *Yield: 72%. ^{1}H NMR (300 MHz, DMSO-d_6) δ 10.44 (s, 4H), 7.46 (s, 4H). ^{13}C NMR (75 MHz, DMSO-d_6) δ 181.09, 150.80, 127.05, 112.89. ATR-FTIR (cm^{-1}): 3400, 3136, 1653, 1558, 1508, 1450, 1348, 1304, 1227, 1188, 1151, 1053, 912, 777, 746, 640, 594.*

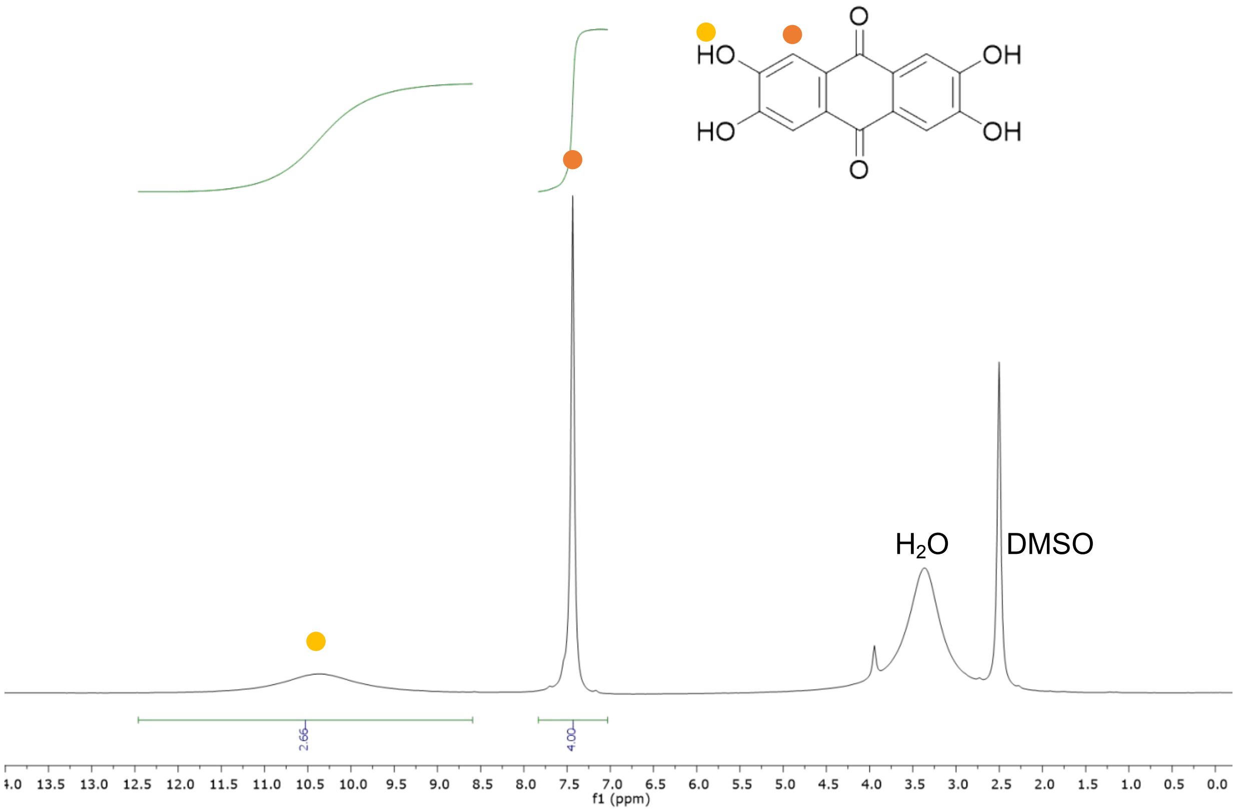

Figure S9. ^{1}H NMR spectrum of 2,3,6,7-tetrahydroxyanthraquinone in DMSO-d_6.

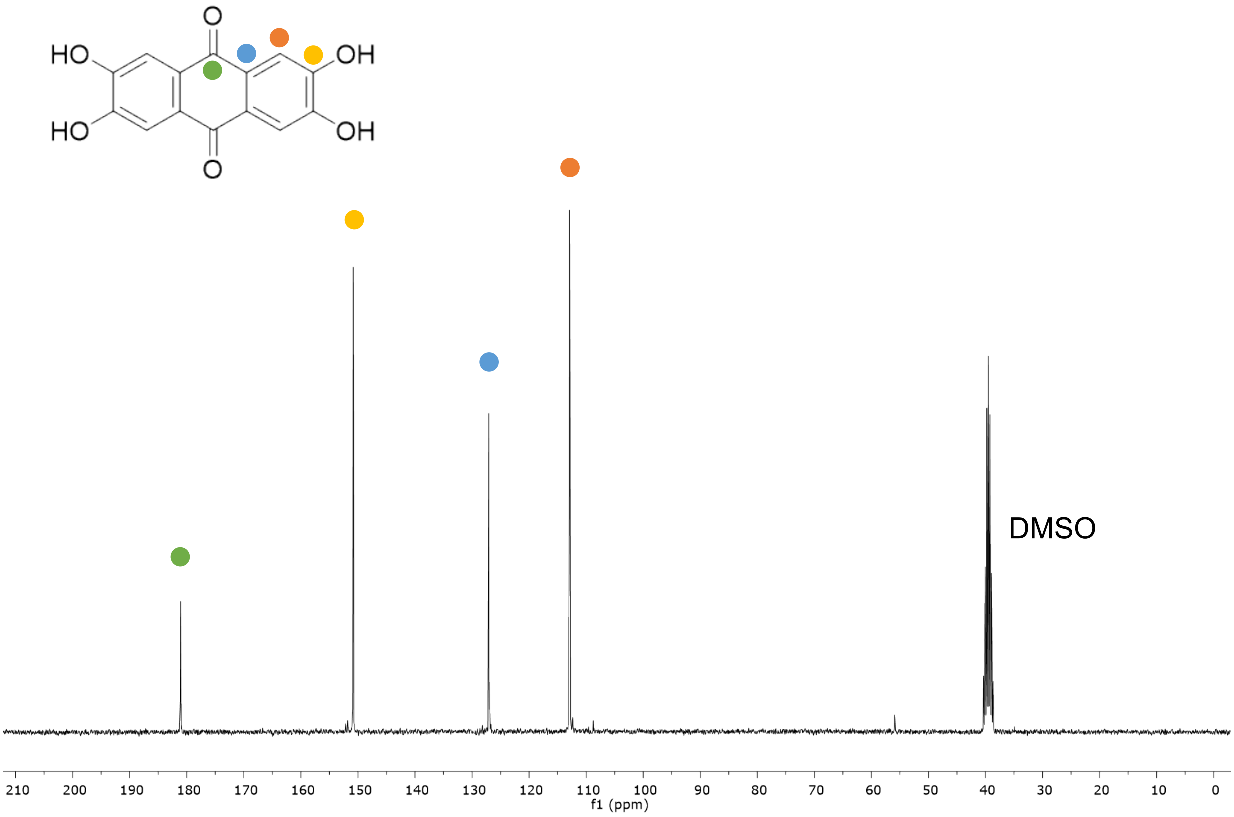

Figure S10. ^{13}C NMR spectrum of 2,3,6,7-tetrahydroxyanthraquinone in DMSO-d_6.

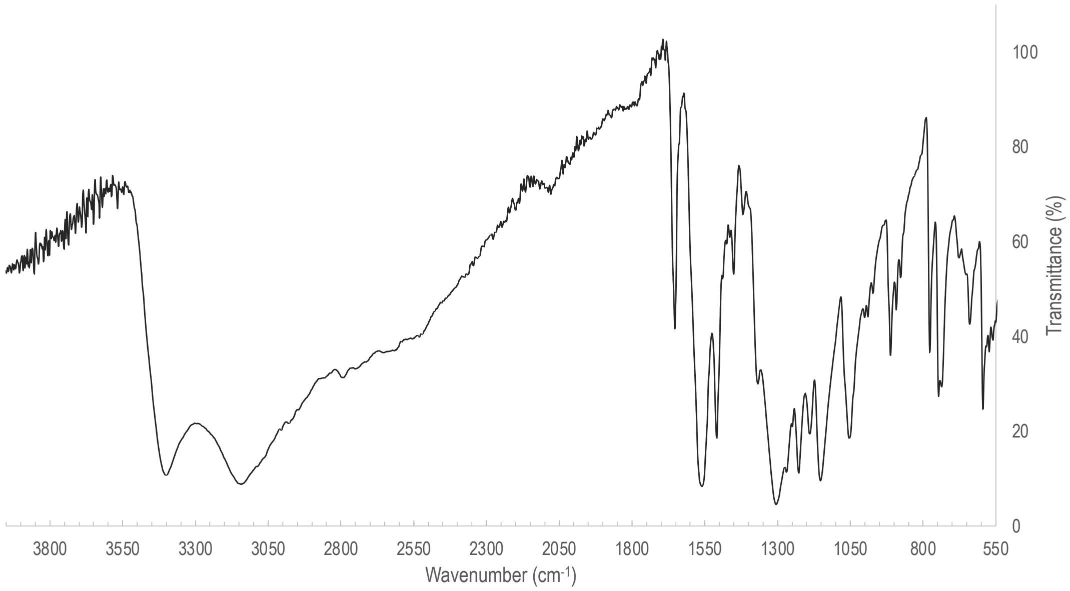

Figure S11. ATR-FTIR spectrum of 2,3,6,7-tetrahydroyanthraquinone.

SI-I-3-2 Polymers Syntheses

Table S1. Utilized ratios of conc. H_2SO_4 and Glacial acetic acid for synthesis of Poly(HHAQ-aldehyde) and its 10wt% MWCNTs composites. Where ***X*** and ***Y*** are in mL.

	Ratio 1	Ratio 2	Ratio 3
X (conc. H_2SO_4)	1.2	2.0	2.8
Y (Glacial acetic acid)	1.6	0.8	-

Table S2. The systematically altered parameters for the optimization of the synthesis of poly(HHAQ-formaldehyde) from HHAQ and formaldehyde.''

Altered Parameters	Temperature (°C)	Crosslinker	MWCNTs Composites (wt.%)	$\frac{V_{Conc.\ H_2SO_4}}{V_{Glacial\ Acetic\ Acid}}$
	90, 120, 160 (autoclave)	THAQ, Glutaraldehyde	0 or 10	1 : 1.33<br>1 : 0.4<br>1 : 0

Synthesis of Poly(HHAQ-formaldehyde)

In a 10 mL round bottom flask equipped with a magnetic stirrer, 0.2 g (0.657 mmol) 1,2,3,5,6,7-hexahydroxyanthraquinone was dissolved in ***X*** mL conc. H_2SO_4 and ***Y*** mL glacial acetic acid (for ***X*** and ***Y***, see Table S1). The resulting mixture was heated to 90 °C while stirring and 0.3856 mL (5.26 mmol; 8.0 eq.) formaldehyde (37 wt.% aq.) was added subsequently in a dropwise fashion. When required, an additional 0.3856 mL formaldehyde (37 wt.% aq.) was added after 17 hours in a dropwise fashion. The solidified reaction mixture was added to 200 mL demineralized water followed by addition of NaOH till pH = 6-7. The polymer was obtained via filtration under reduced pressure, subsequently washed with hot THF (1-3x 100 mL) till no colored monomer solution was obtained anymore, filtered and dried overnight under vacuum at 60 °C. *Yield: 84%. ATR-FTIR (cm^{-1}): 3255, 1691, 1589, 1450, 1243, 1078.*

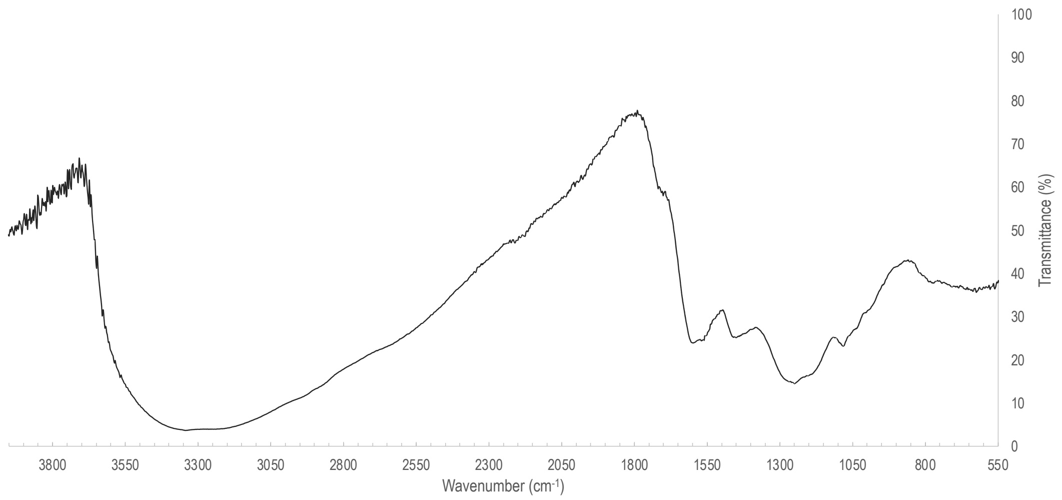

Figure S12. ATR-FTIR spectrum of Poly(HHAQ-formaldehyde).

Synthesis of Poly(HHAQ-formaldehyde)-10wt% MWCNTs composite

To a 25 mL round bottom flask, 0.02 g (10 wt.%) MWCNTs, a stirring bar, ***X*** mL conc. H_2SO_4 and ***Y*** mL glacial acetic acid were added (for ***X*** and ***Y***, see Table S1). The MWCNTs were dispersed in the solution by bath sonication for 2 hours. After 1 hour of sonicating, another $\frac{x}{2}$ mL conc. H_2SO_4 and $\frac{y}{2}$ mL glacial acetic acid were added. After the sonication, 0.2 g (0.657 mmol) 1,2,3,5,6,7-hexahydroxyanthraquinone was introduced and the resulting dispersion was heated to 90 ^oC while stirring and 0.3856 mL (5.26 mmol; 8.0 eq.) formaldehyde (37 wt.% aq.) was added subsequently in a dropwise fashion. When required, an additional 0.3856 mL formaldehyde (37 wt.% aq.) was added after 17 hours in a dropwise fashion. The solidified reaction mixture was added to 200 mL demineralized water followed by addition of NaOH till pH = 6-7. The polymer was obtained via filtration under reduced pressure, subsequently washed with hot THF (1-3x 100 mL) till no colored monomer solution was obtained anymore, filtered and dried overnight under vacuum at 60 ^0C. *Yield: 83%. ATR-FTIR (cm^{-1}): 3080, 2926, 2850, 1711, 1587, 1428, 1240, 1193, 1048.*

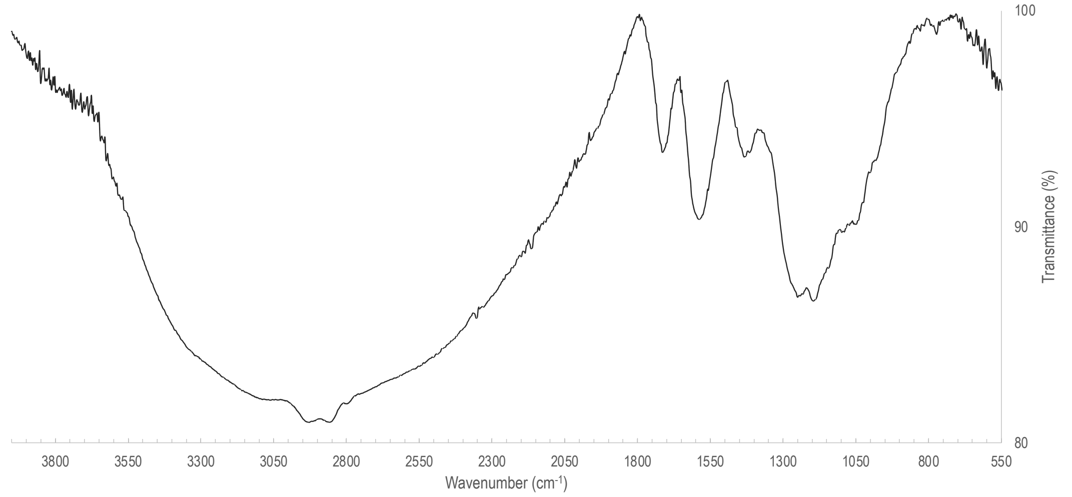

Figure S13. ATR-FTIR spectrum of Poly(HHAQ-formaldehyde)-10wt% MWCNTs composite.

Synthesis of Poly(HHAQ-glutaraldehyde)

In a 10 mL round bottom flask equipped with a magnetic stirrer, 0.2 g (1.64 mmol) 1,2,3,5,6,7-hexahydroxyanthraquinone was dissolved in ***X*** mL conc. H_2SO_4 and ***Y*** mL glacial acetic acid (for ***X*** and ***Y***, see Table S1). The resulting mixture was heated to 90 ^{o}C while stirring and 0.1608 mL (1.64 mmol;1.0 eq.) glutaraldehyde (50 wt.% aq.) was added subsequently in a dropwise fashion. After 17 hours, an additional 0.1608 mL (1.64 mmol; 1.0 eq.) glutaraldehyde (50 wt.% aq.) was added subsequently in a dropwise fashion. The solidified reaction mixture was added to 200 mL demineralized water followed by addition of NaOH till pH = 6-7. The polymer was obtained via filtration under reduced pressure, subsequently washed with hot THF (1-3x 100 mL) till no colored monomer solution was obtained anymore, filtered and dried overnight under vacuum at 60 ^{0}C. *Yield: 59%. ATR-FTIR (cm^{-1}): 3544, 3311, 2929, 2863, 1718, 1590, 1319, 1243, 1213, 1172, 1108, 1074, 997, 771, 696, 638.*

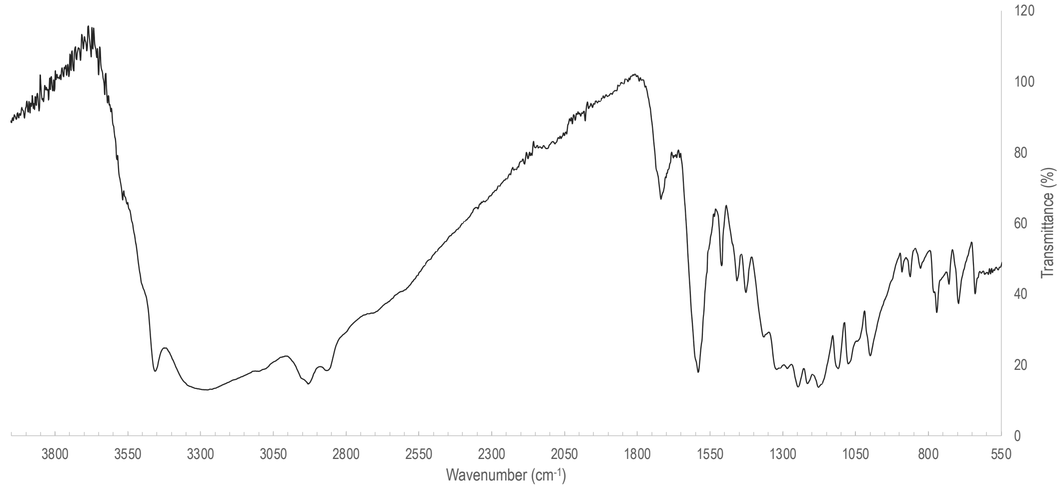

Figure S14. ATR-FTIR spectrum of Poly(HHAQ-glutaraldehyde).

Synthesis of Poly(HHAQ-glyoxal)

In a 10 mL round bottom flask equipped with a magnetic stirrer, 0.2 g (0.657 mmol) 1,2,3,5,6,7-hexahydroxyanthraquinone was dissolved in ***X*** mL conc. H_2SO_4 and ***Y*** mL glacial acetic acid (for ***X*** and ***Y***, see Table S1). The resulting mixture was heated to 90 ^oC while stirring and 0.2996 mL (6.256 mmol; 4.0 eq.) glyoxal (40 wt.% aq.) was added subsequently in a dropwise fashion. An additional 0.2996 mL (6.256 mmol; 4.0 eq.) glyoxal (40 wt.% aq.) was added after 2 hours in a dropwise fashion. The solidified reaction mixture was added to 200 mL demineralized water followed by addition of NaOH till pH = 6-7. The polymer was obtained via filtration under reduced pressure, subsequently washed with hot THF (1-3x 100 mL) till no colored monomer solution was obtained anymore, filtered and dried overnight under vacuum at 60 ^0C. *Yield: 61%. ATR-FTIR (cm^{-1}): 3255, 2875, 1695, 1576, 1253, 1068, 762.*

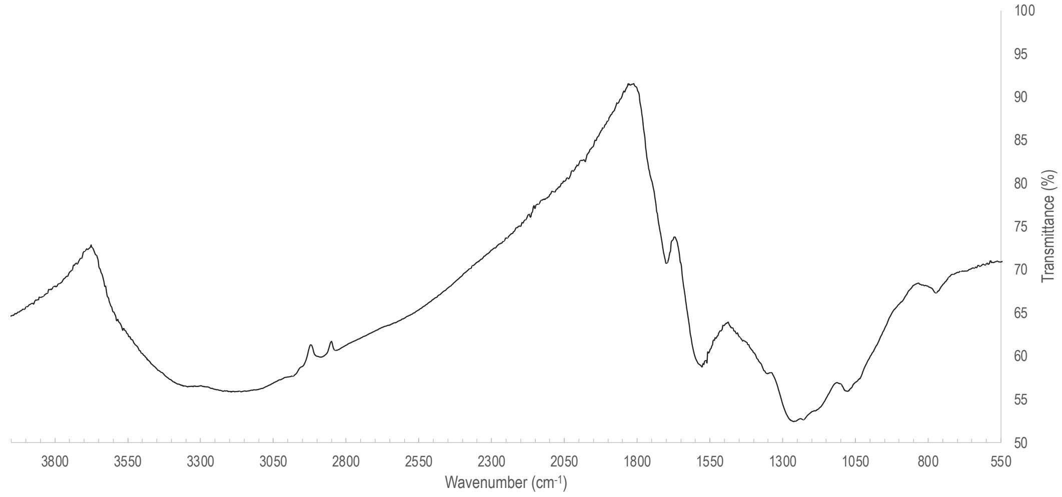

Figure S15. ATR-FTIR spectrum of Poly(HHAQ-*alt*-glyoxal).

Synthesis of Poly(THAQ-formaldehyde)

In a 10 mL round bottom flask equipped with a magnetic stirrer, 0.2 g (0.735 mmol) 2,3,6,7-tetrahydroxyanthraquinone was dissolved in 2.8 mL conc. H_2SO_4. The resulting mixture was heated to 90 ^{o}C while stirring and 0.4308 mL (5.88 mmol; 8.0 eq.) formaldehyde (37 wt.% aq.) was added subsequently in a dropwise fashion. After 4-6h, the solidified reaction mixture was added to 200 mL demineralized water followed by addition of NaOH till pH = 6-7. The polymer was obtained via filtration under reduced pressure, subsequently washed with hot THF (3x 100 mL) and dried overnight under vacuum at 60 ^{0}C. *Yield: 81%. ATR-FTIR (cm^{-1}): 3113, 2937, 2866, 1698, 1651, 1568, 1437, 1305, 1167, 1146, 1045, 912.*

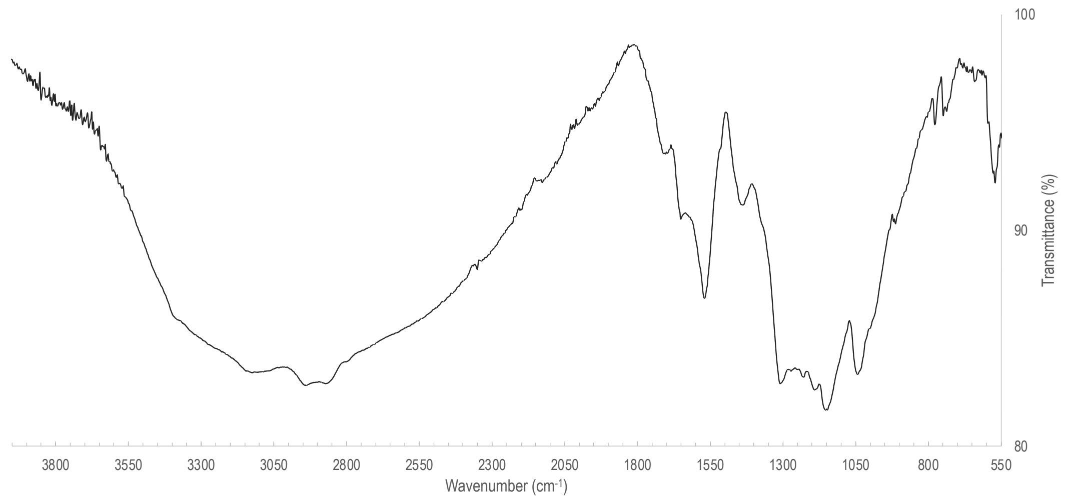

Figure S16. ATR-FTIR spectrum of Poly(THAQ-formaldehyde).

Synthesis of Poly(THAQ-formaldehyde)-10 wt% MWCNTs composite

To a 25 mL round bottom flask, 0.02 g (10 wt.%) MWCNTs, a stirring bar and 2.8 mL conc. H_2SO_4 were added. The MWCNTs were dispersed by bath sonication for 2 hours. After 1 hour of sonicating, another 1.4 mL conc. H_2SO_4 was added. After the sonication, 0.2 g (0.735 mmol) 2,3,6,7-tetrahydroxyanthraquinone was introduced and the resulting dispersion was heated to 90 ^{o}C while stirring and 0.4308 mL (5.88 mmol; 8.0 eq.) formaldehyde (37 wt.% aq.) was added subsequently in a dropwise fashion. After 17h, the solidified reaction mixture was added to 200 mL demineralized water followed by addition of NaOH till pH = 6-7. The polymer was obtained via filtration under reduced pressure, subsequently washed with hot THF (4x 100 mL) and dried overnight under vacuum at 60 ^{0}C. *Yield: 81%. ATR-FTIR (cm^{-1}): 3057, 2935, 2853, 1718, 1560, 1437, 1299, 1190, 1136, 1032.*

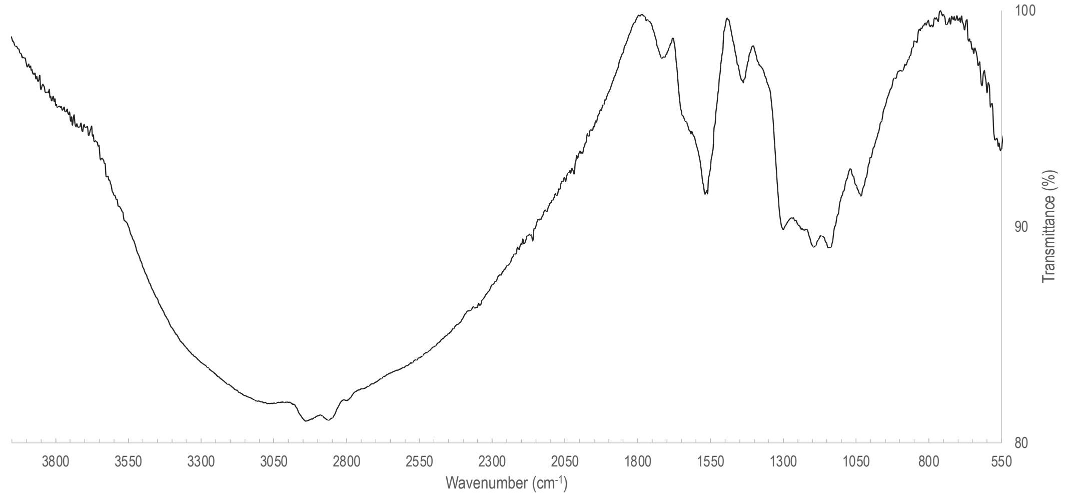

Figure S17. ATR-FTIR spectrum of Poly(THAQ-formaldehyde)-10 wt% MWCNTs composite.

SI-II Results and Discussion

SI-II-1 Anticipated Reaction Mechanism Polymerization HHAQ

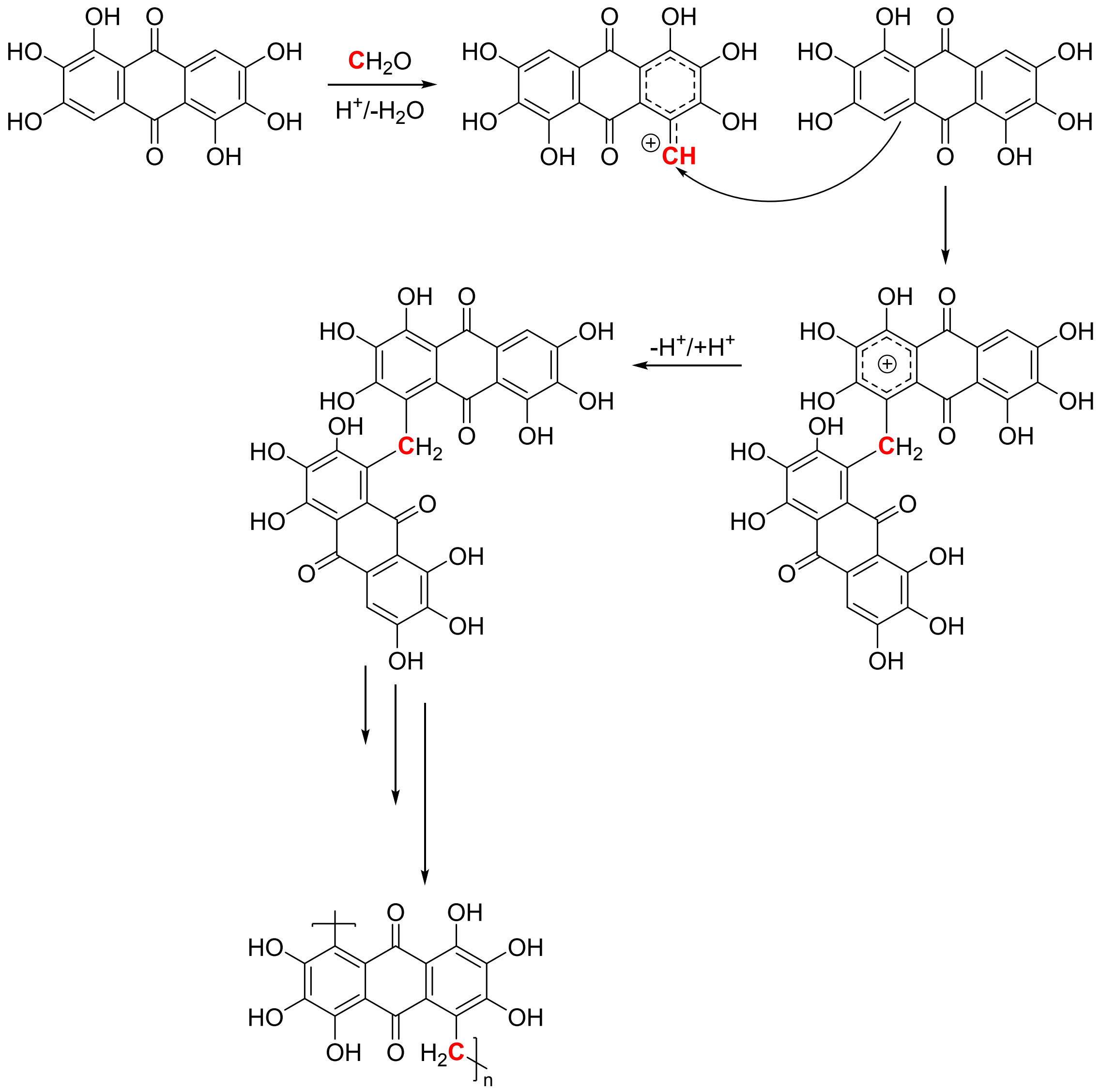

Scheme S1. Anticipated Reaction Mechanism of the condensation polymerization of HHAQ. Inspired by mechanistical studies found in literature.[4]

SI-II-2 Images Solidified Polymerization Mixture

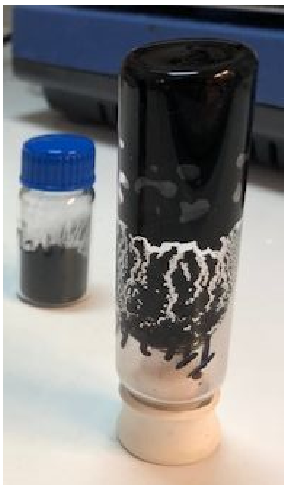

Figure S18. Solidified reaction mixture of HHAQ polymerization with formaldehyde.

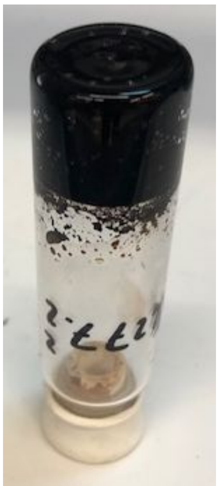

Figure S19. Solidified reaction mixture of THAQ polymerization with formaldehyde.

SI-II-3 SEM Images

SI-II-3-1 Poly(HHAQ-formaldehyde)-10 wt.% MWCNT composite

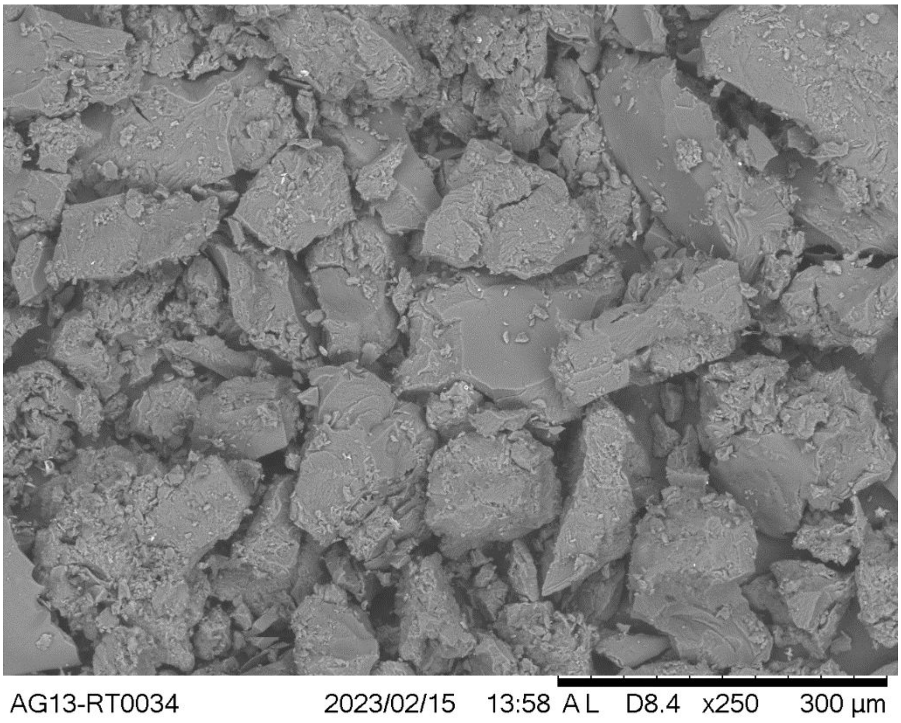

Figure S20. SEM image of Poly(HHAQ-formaldehyde)-10 wt.% MWCNT composite synthesized in conc. H_2SO_4 at 250x magnification.

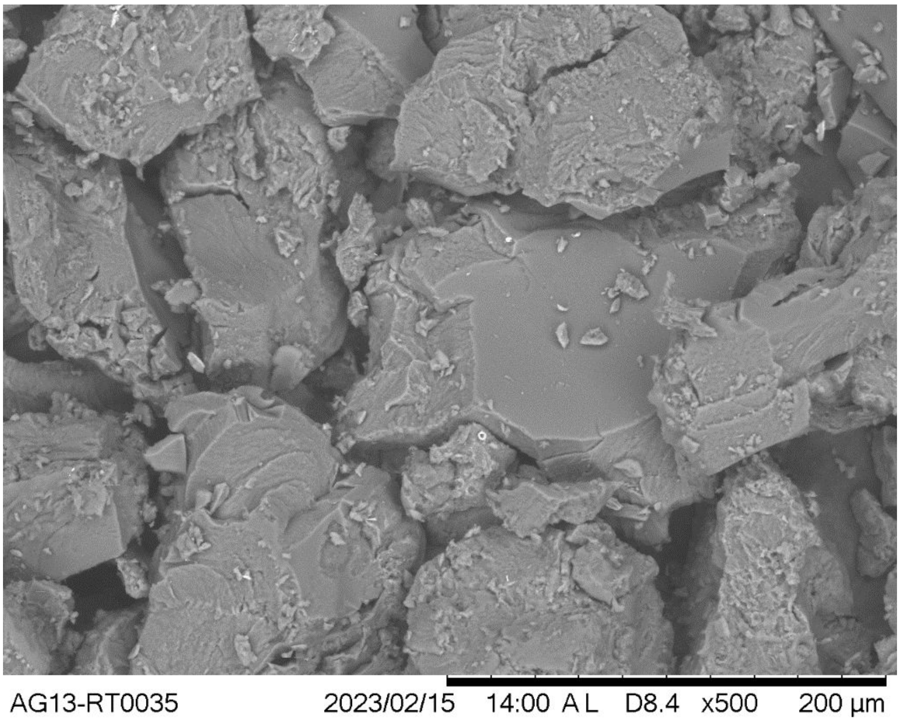

Figure S21. SEM image of Poly(HHAQ-formaldehyde)-10 wt.% MWCNT composite synthesized in conc. H_2SO_4 at 500x magnification.

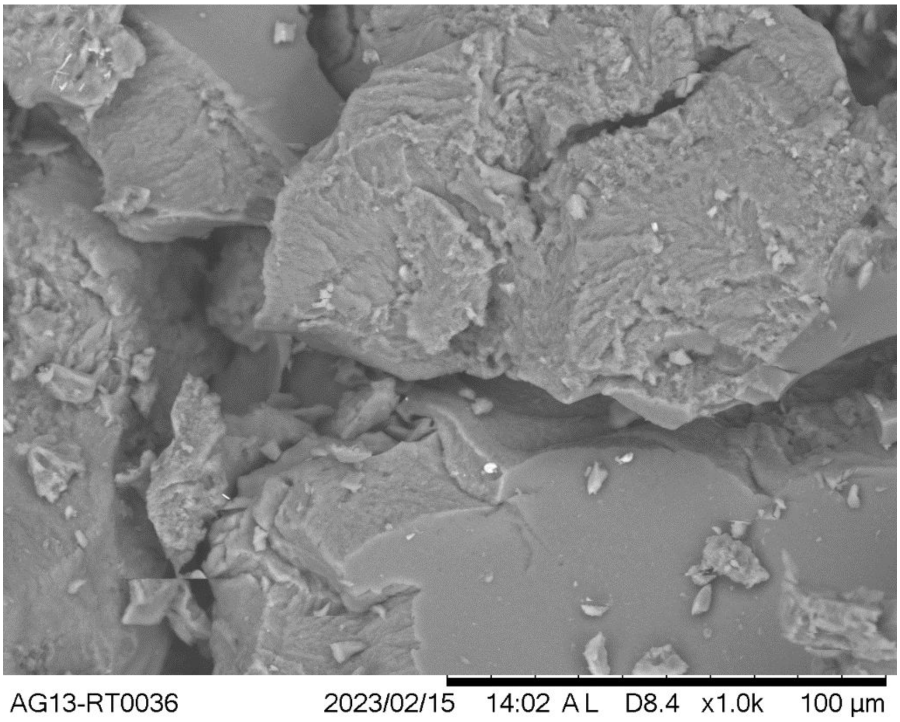

Figure S23. SEM image of Poly(HHAQ-formaldehyde)-10 wt.% MWCNT composite synthesized in conc. H_2SO_4 at 1000x magnification.

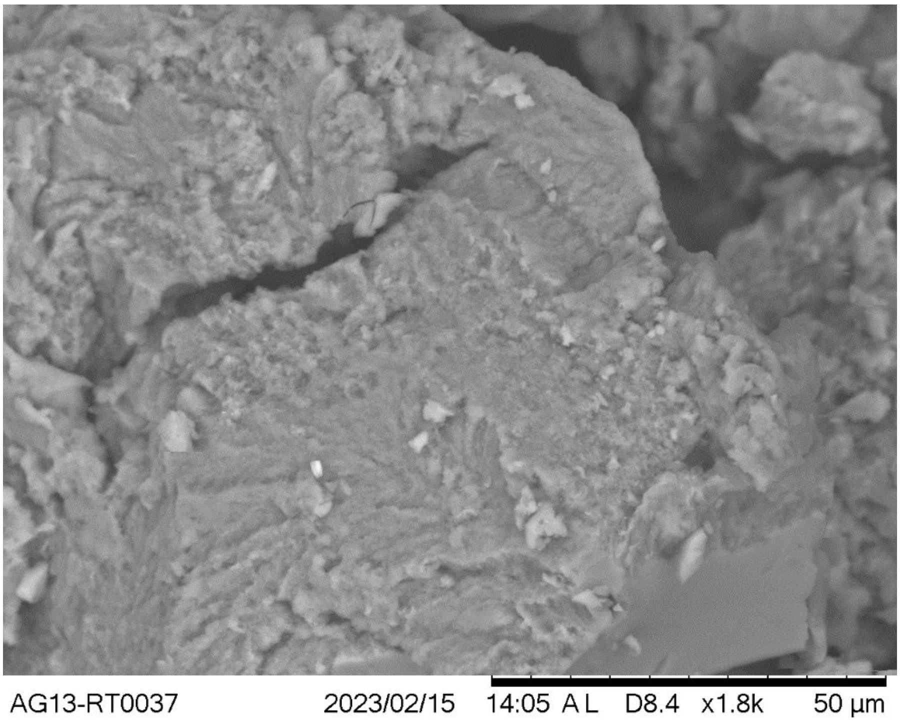

Figure S22. SEM image of Poly(HHAQ-formaldehyde)-10 wt.% MWCNT composite synthesized in conc. H_2SO_4 at 1800x magnification.

SI-II-3-2 Poly(THAQ-formaldehyde)-10 wt.% MWCNT composite | Solvent: Conc. H_2SO_4

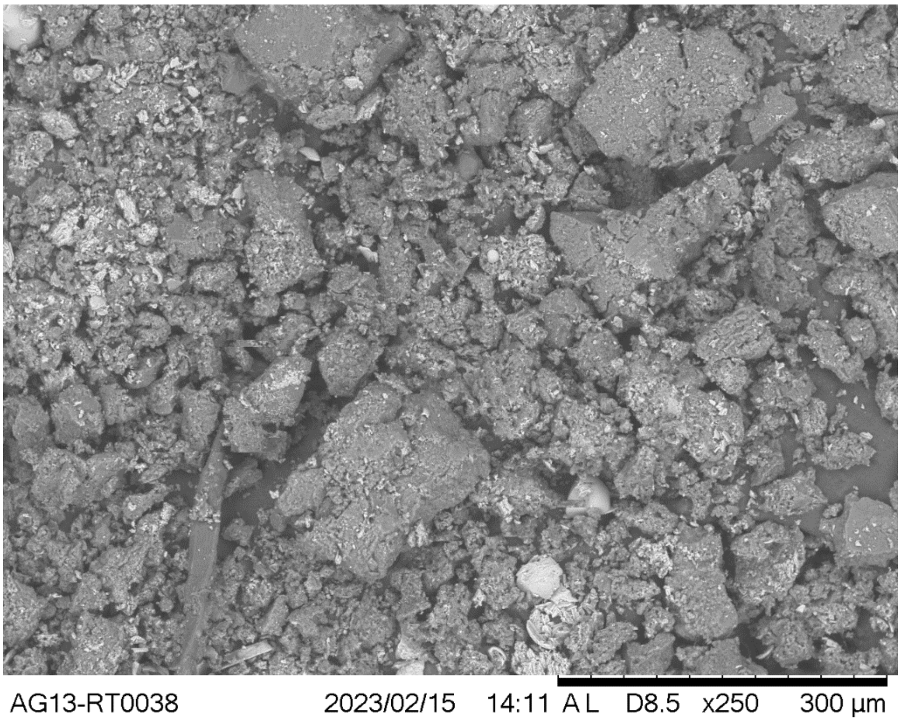

Figure S24. SEM image of Poly(THAQ-formaldehyde)-10 wt.% MWCNT composite synthesized in conc. H_2SO_4 at 250x magnification.

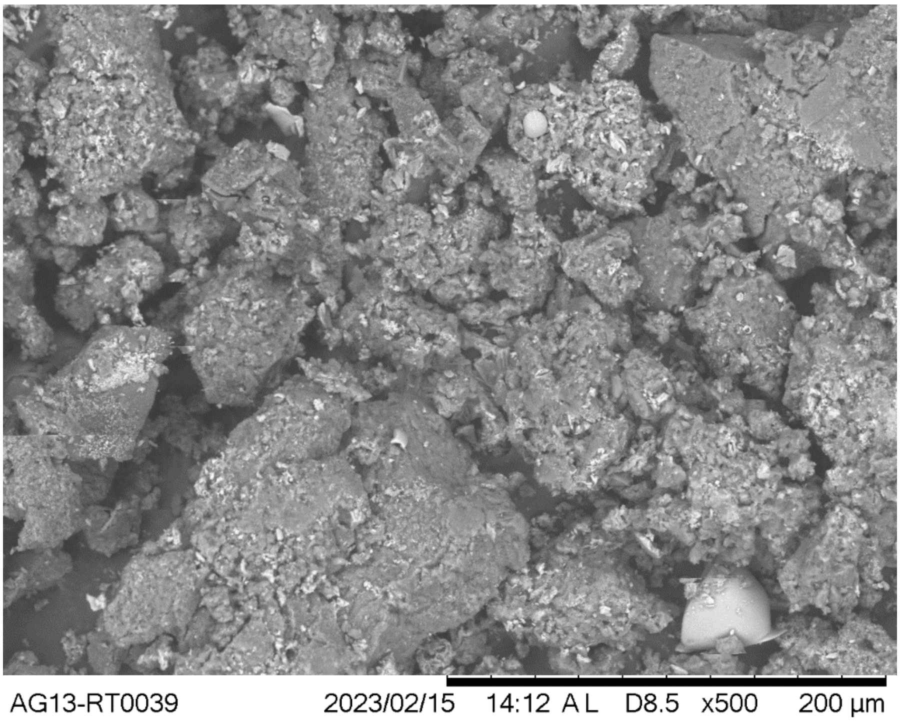

Figure S25. SEM image of Poly(THAQ-formaldehyde)-10 wt.% MWCNT composite synthesized in conc. H_2SO_4 at 500x magnification.

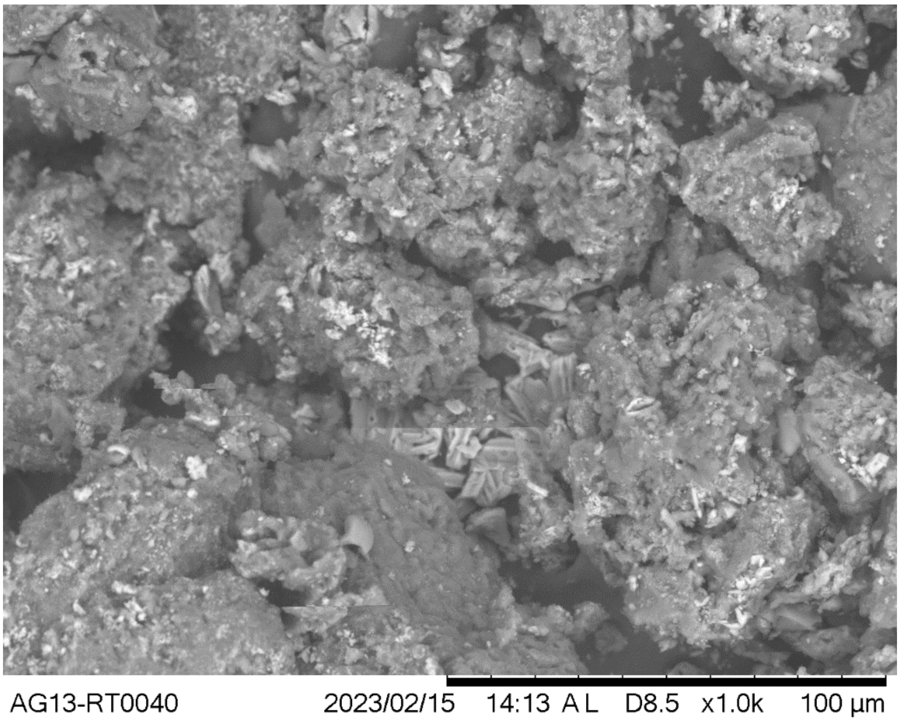

Figure S26. SEM image of Poly(THAQ-formaldehyde)-10 wt.% MWCNT composite synthesized in conc. H_2SO_4 at 1000x magnification.

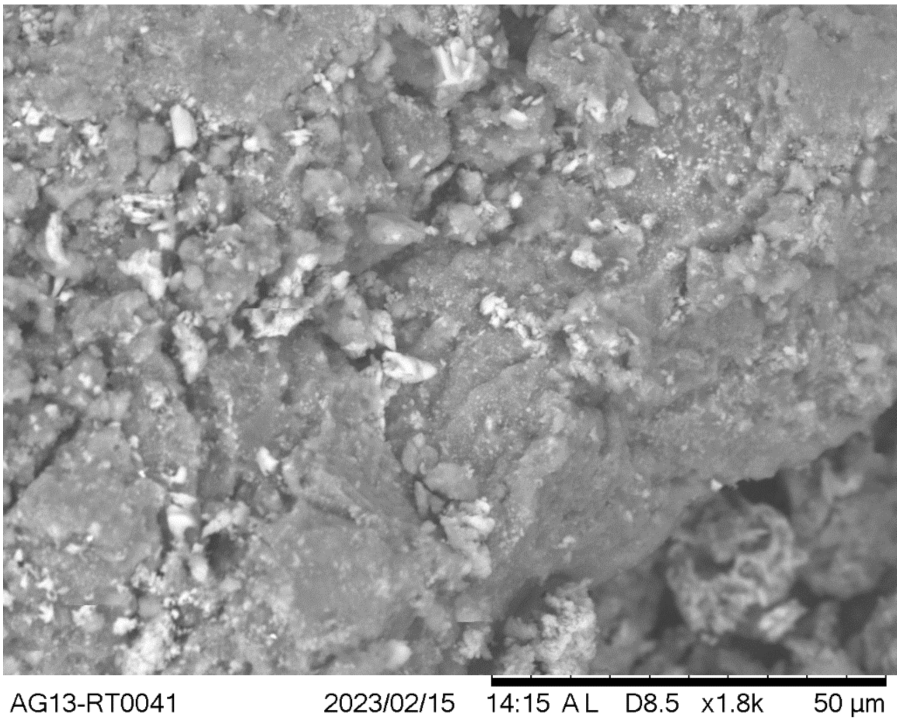

Figure S27. SEM image of Poly(THAQ-formaldehyde)-10 wt.% MWCNT composite synthesized in conc. H_2SO_4 at 1800x magnification.

SI-II-4 Electrochemistry

SI-II-4-1 Electrochemical Mechanisms HHAQ

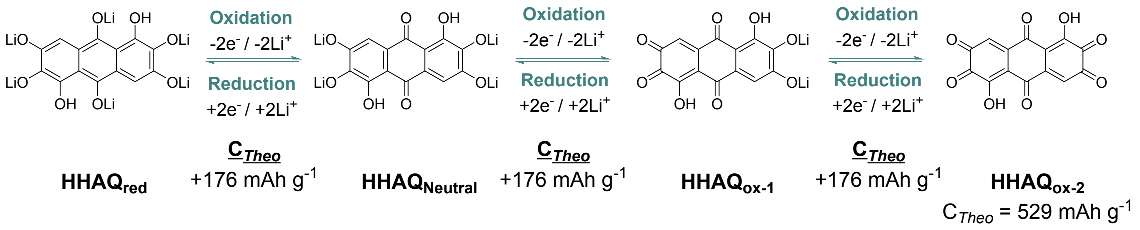

Scheme S2. Anticipated electrochemical mechanism of HHAQ including the oxidized ($HHAQ_{ox-1}$ and $HHAQ_{ox-2}$) and reduced ($HHAQ_{red}$) states and their related theoretical capacities (C_{Theo}).

SI-II-4-2 Electrochemical Mechanisms OHAQ

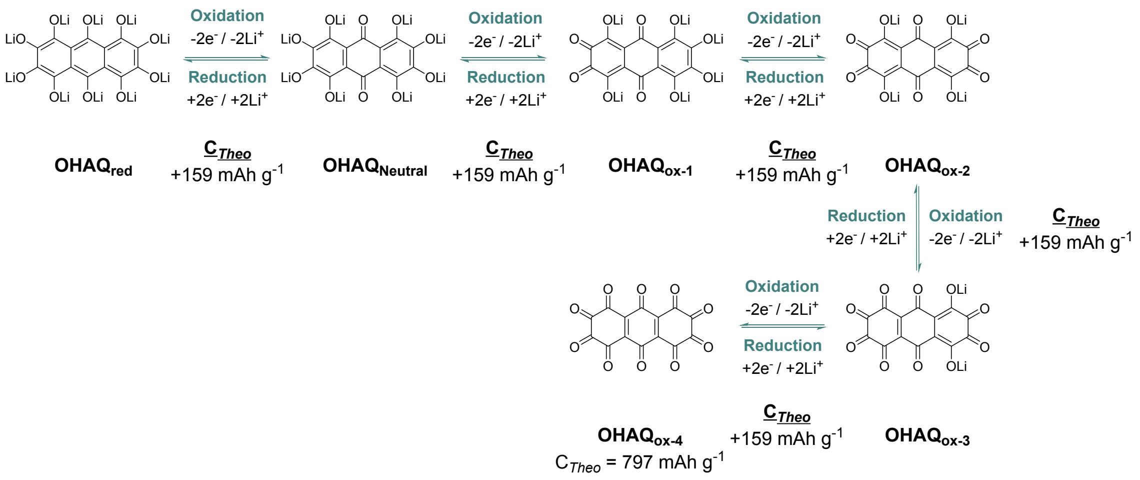

Scheme S3. Anticipated electrochemical mechanism of OHAQ including the oxidized ($OHAQ_{ox-1}$ till $HHAQ_{ox-4}$) and reduced ($OHAQ_{red}$) states and their related theoretical capacities (C_{Theo}).

SI-II-4-3 Cyclic Voltammograms THAQ in 1.0M $LiPF_6$ in EC:DEC (1:1, v:v)

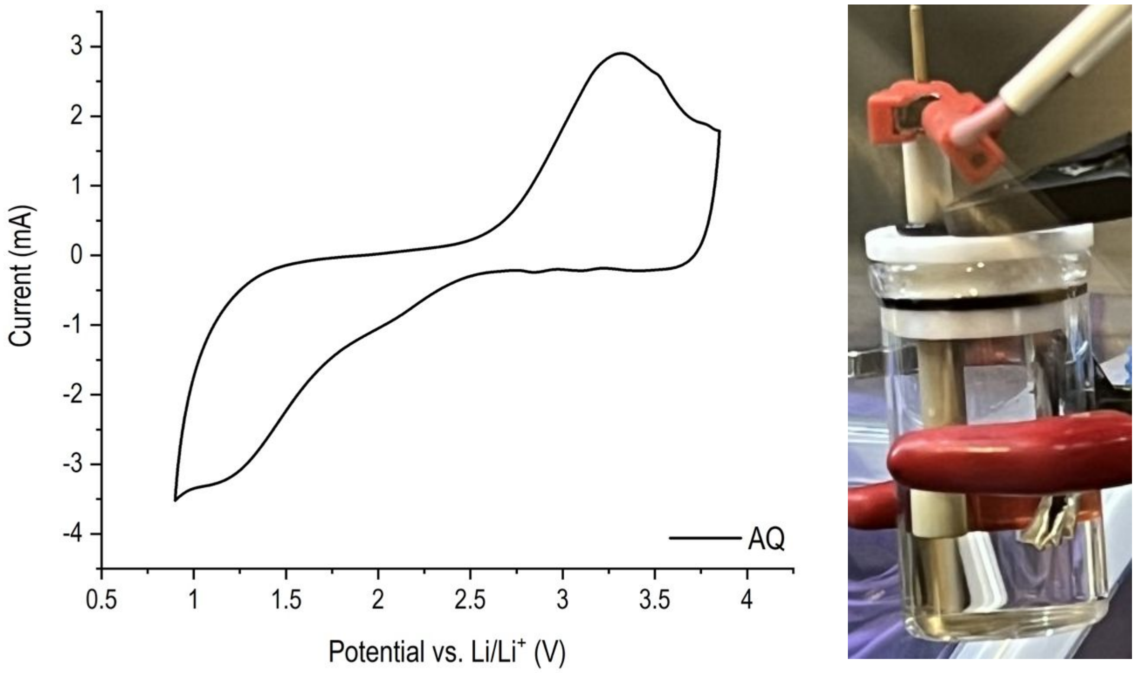

Figure S28. Cyclic Voltammogram (scan rate = 5 mV/s) of the anthraquinone subgroup of THAQ in a three-electrode setup using Li metal as counter and reference electrode and a [THAQ:C_{65}:SEBS] = [40:50:10] electrode formulation on Glassy Carbon as working electrode with 1.0M $LiPF_6$ in EC:DEC (1:1, v:v) as electrolyte.

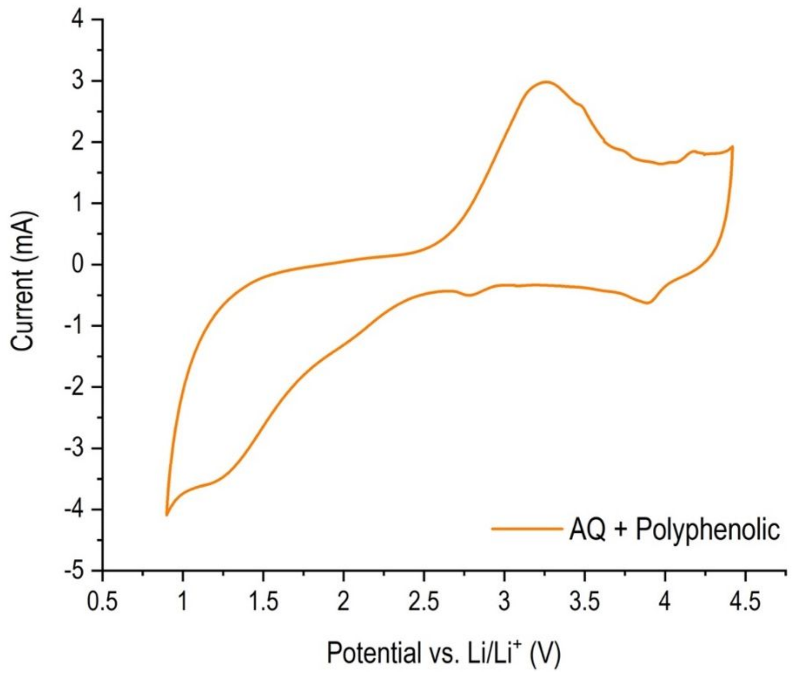

Figure S29. Cyclic Voltammogram (scan rate = 5 mV/s) of the polyhenolic and anthraquinone subgroup of THAQ in a three-electrode setup using Li metal as counter and reference electrode and a [THAQ:C_{65}:SEBS] = [40:50:10] electrode formulation on Glassy Carbon as working electrode with 1.0M $LiPF_6$ in EC:DEC (1:1, v:v) as electrolyte.

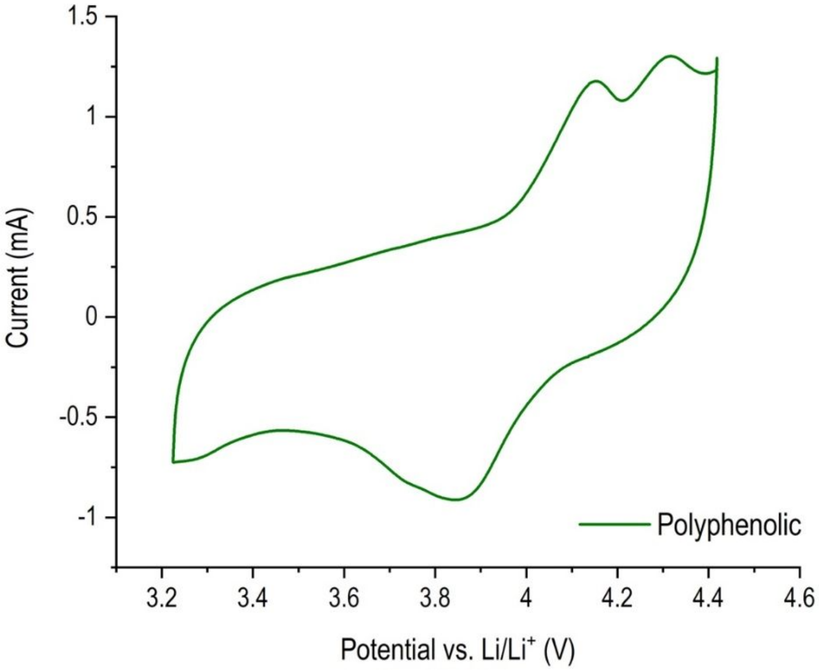

Figure S30. Cyclic Voltammogram (scan rate = 5 mV/s) of the polyphenolic subgroup of THAQ in a three-electrode setup using Li metal as counter and reference electrode and a [THAQ:C_{65}:SEBS] = [40:50:10] electrode formulation on Glassy Carbon as working electrode with 1.0M $LiPF_6$ in EC:DEC (1:1, v:v) as electrolyte.

SI-II-4-4 Cyclic Voltammograms HHAQ in 1.0M $LiPF_6$ in EC:DEC (1:1, v:v)

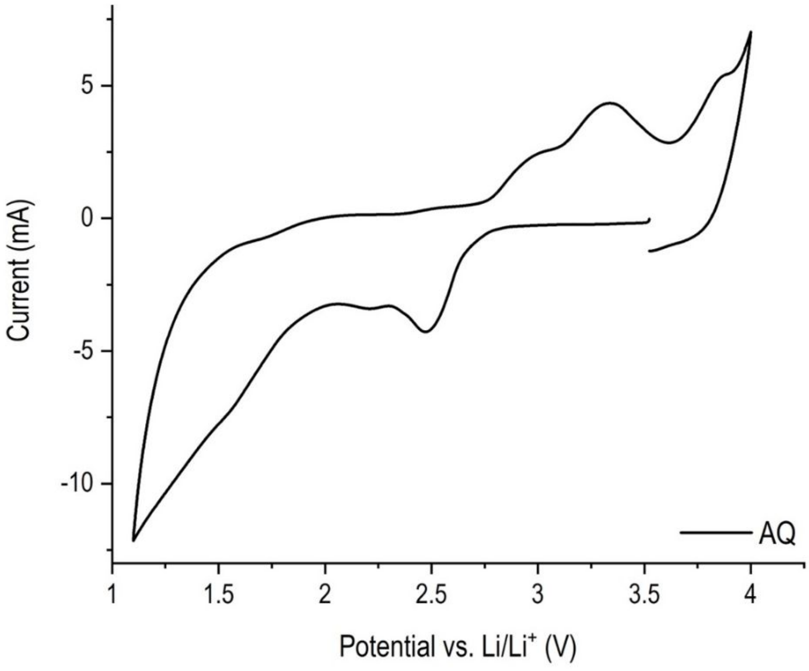

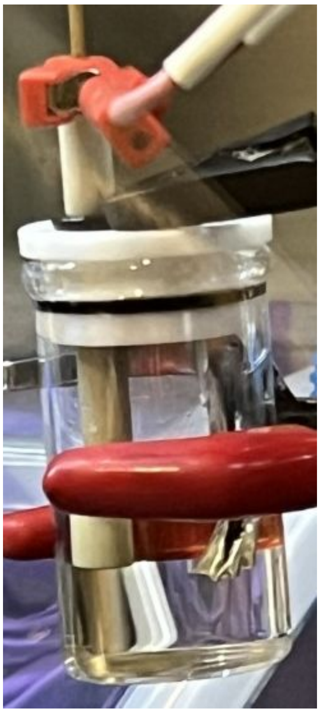

Figure S31. Cyclic Voltammogram (scan rate = 5 mV/s) of the anthraquinone subgroup of HHAQ in a three-electrode setup using Li metal as counter and reference electrode and a [HHAQ:C_{65}:SEBS] = [40:50:10] electrode formulation on Glassy Carbon as working electrode with 1.0M $LiPF_6$ in EC:DEC (1:1, v:v) as electrolyte.

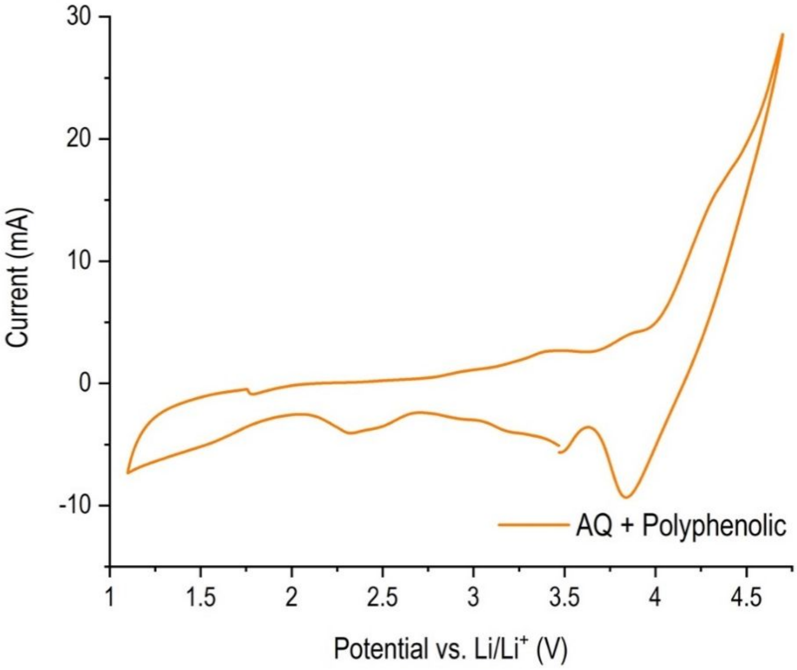

Figure S32. Cyclic Voltammogram (scan rate = 5 mV/s) of the anthraquinone and polyphenolic subgroup of HHAQ in a three-electrode setup using Li metal as counter and reference electrode and a [HHAQ:C_{65}:SEBS] = [40:50:10] electrode formulation on Glassy Carbon as working electrode with 1.0M $LiPF_6$ in EC:DEC (1:1, v:v) as electrolyte.

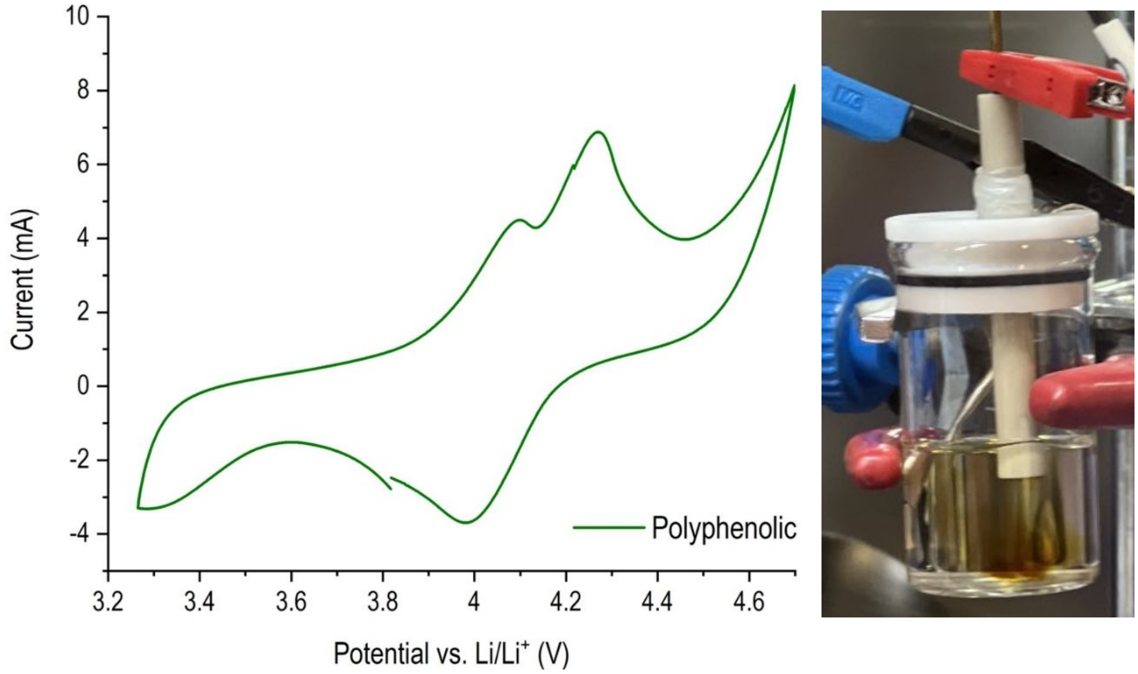

Figure S33. Cyclic Voltammogram (scan rate = 5 mV/s) of the polyphenolic subgroup of HHAQ in a three-electrode setup using Li metal as counter and reference electrode and a [HHAQ:C_{65}:SEBS] = [40:50:10] electrode formulation on Glassy Carbon as working electrode with 1.0M $LiPF_6$ in EC:DEC (1:1, v:v) as electrolyte.

SI-II-4-5 Cyclic Voltammograms OHAQ in 1.0M $LiPF_6$ in EC:DEC (1:1, v:v)

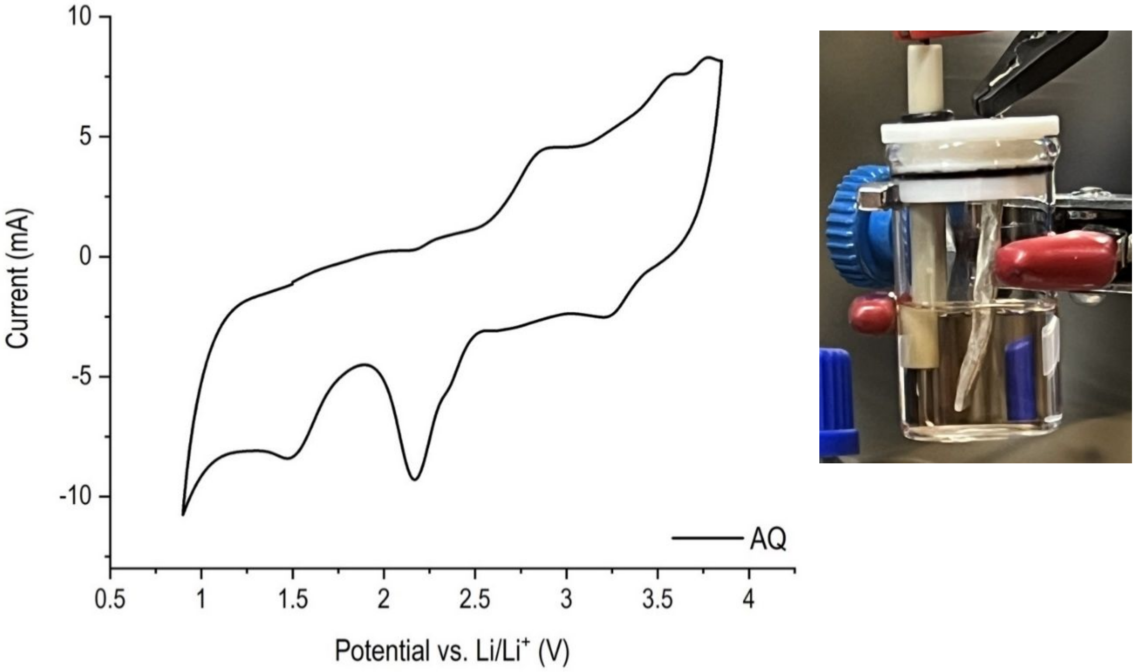

Figure S34. Cyclic Voltammogram (scan rate = 5 mV/s) of the anthraquinone subgroup of OHAQ in a three-electrode setup using Li metal as counter and reference electrode and a [OHAQ:C_{65}:SEBS] = [40:50:10] electrode formulation on Glassy Carbon as working electrode with 1.0M $LiPF_6$ in EC:DEC (1:1, v:v) as electrolyte.

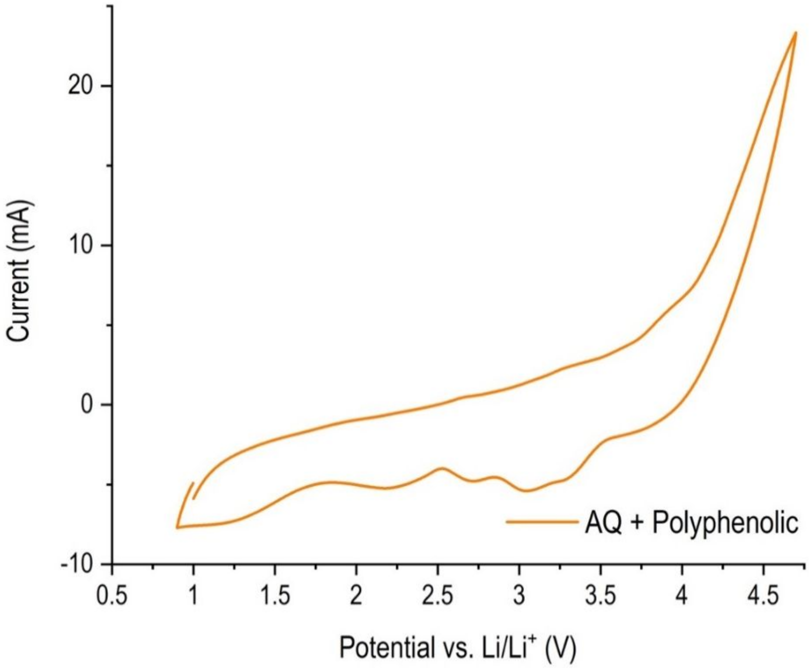

Figure S35. Cyclic Voltammogram (scan rate = 5 mV/s) of the anthraquinone and polyphenolic subgroup of OHAQ in a three-electrode setup using Li metal as counter and reference electrode and a [OHAQ:C_{65}:SEBS] = [40:50:10] electrode formulation on Glassy Carbon as working electrode with 1.0M $LiPF_6$ in EC:DEC (1:1, v:v) as electrolyte.

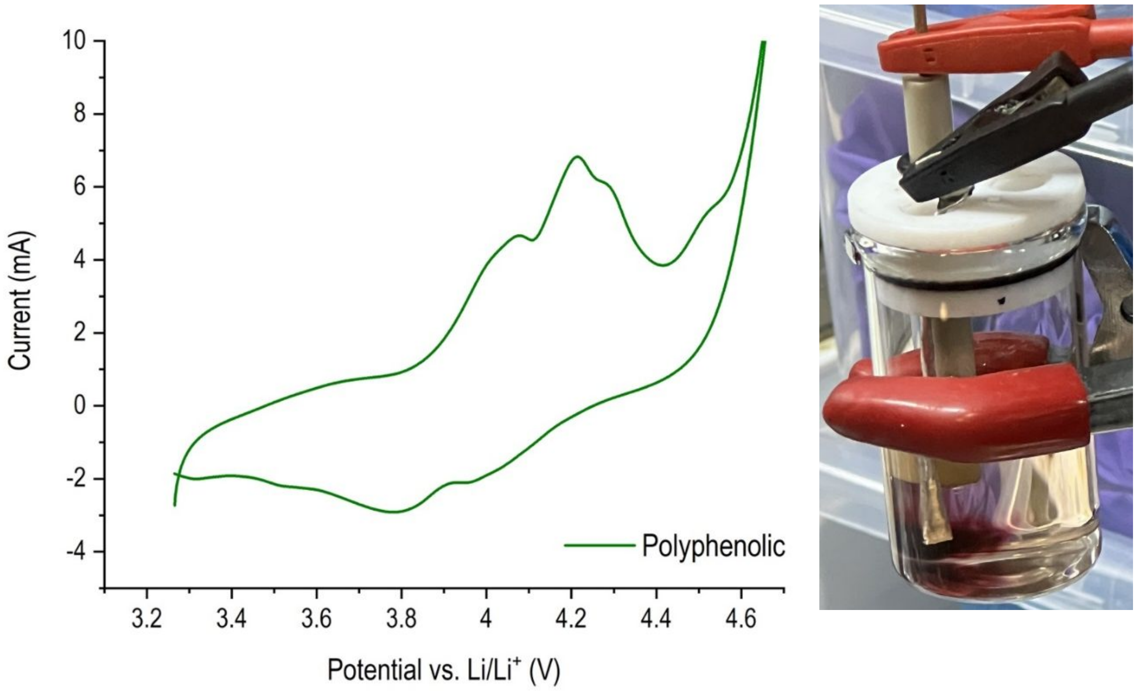

Figure S36. Cyclic Voltammogram (scan rate = 5 mV/s) of the polyphenolic subgroup of OHAQ in a three-electrode setup using Li metal as counter and reference electrode and a [OHAQ:C_{65}:SEBS] = [40:50:10] electrode formulation on Glassy Carbon as working electrode with 1.0M $LiPF_6$ in EC:DEC (1:1, v:v) as electrolyte.

SI-II-4-6 Galvanostatic cycling THAQ in lithium metal battery

SI-II-4-6-A Electrolyte: 0.3 M LiTFSI/[PY13][TFSI] ionic liquid

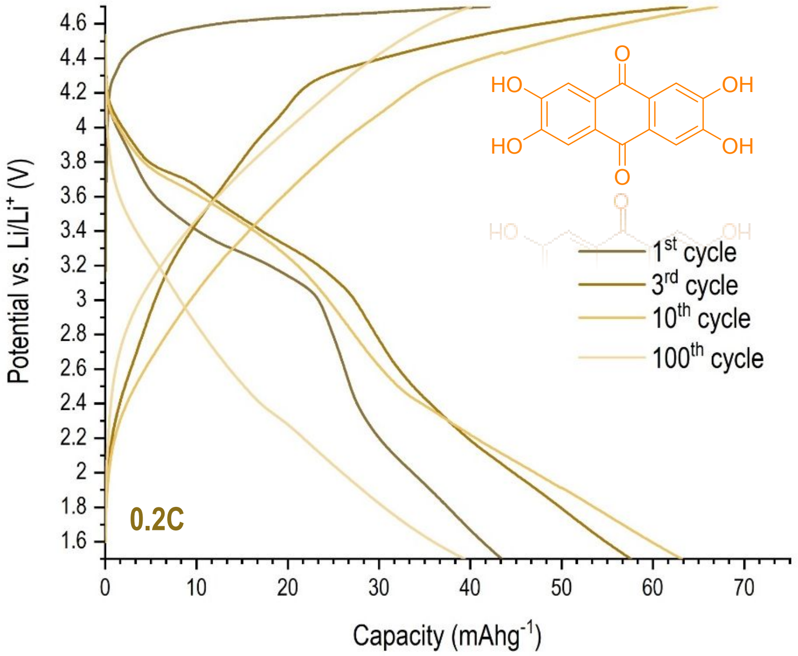

Figure S37. First 100 galvanostatic charge-discharge cycles (C-rate = 0.2C) of THAQ in lithium metal battery, using [Act. Mat.:C_{65}:SEBS] = [40:50:10] as electrode formulation and 0.3M LiTFSI/[PY13][TFSI] ionic liquid as electrolyte.

SI-II-4-7 Galvanostatic cycling HHAQ in lithium metal battery

SI-II-4-7-A Electrolyte: 0.3 M LiTFSI/[PY13][TFSI] ionic liquid

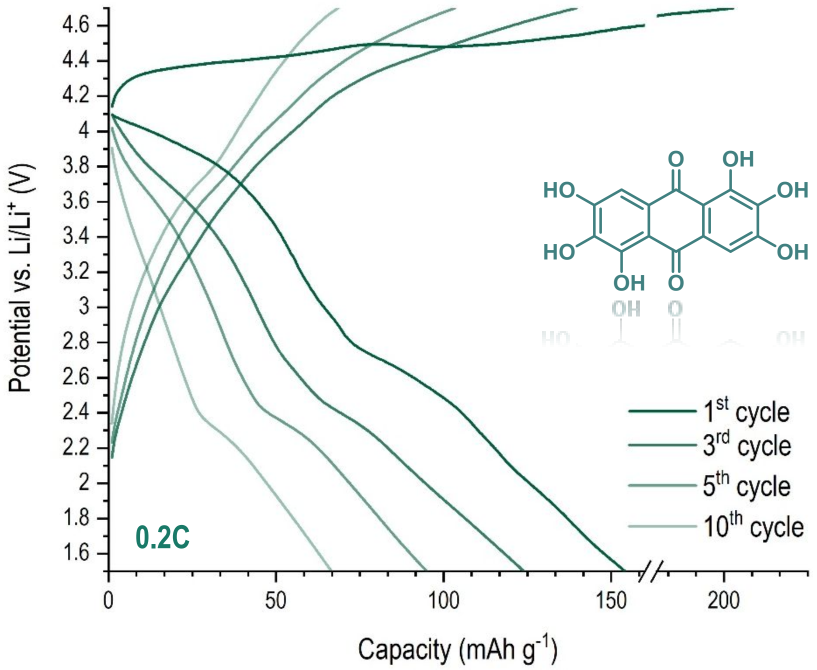

Figure S38. First 10 galvanostatic charge-discharge cycles (C-rate = 0.2C) of HHAQ in lithium metal battery, using [Act. Mat.:C_{65}:SEBS] = [40:50:10] as electrode formulation and 0.3M LiTFSI/[PY13][TFSI] ionic liquid as electrolyte.

SI-II-4-8 Galvanostatic cycling OHAQ in lithium metal battery

SI-II-4-8-A Electrolyte: 0.3 M LiTFSI/[PY13][TFSI] ionic liquid

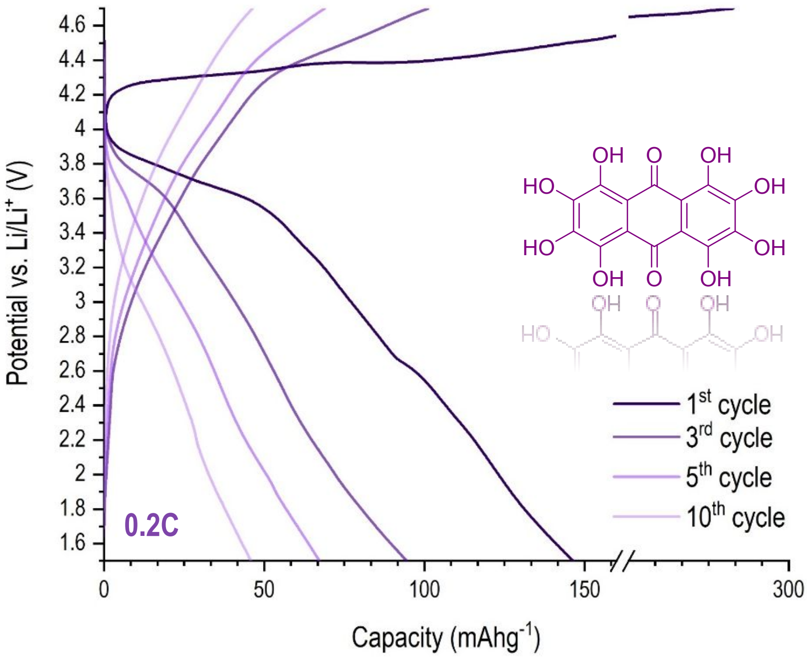

Figure S39. First 10 galvanostatic charge-discharge cycles (C-rate = 0.2C) of OHAQ in lithium metal battery, using [Act. Mat.:C_{65}:SEBS] = [40:50:10] as electrode formulation and 0.3M LiTFSI/[PY13][TFSI] ionic liquid as electrolyte.

SI-II-4-9 Galvanostatic cycling poly(HHAQ-glyoxal) in lithium metal battery

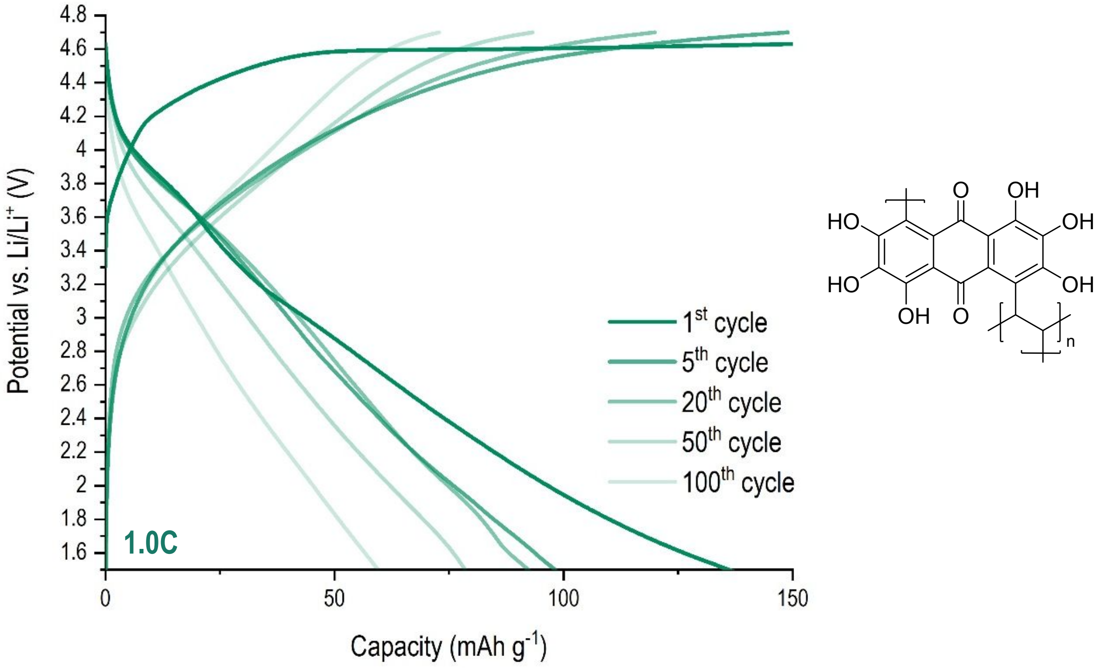

Figure S40. The 1st, 5th, 20th, 50th and 100th galvanostatic charge-discharge cycle (C-rate = 1.0C) of poly(HHAQ-glyoxal) in a lithium metal battery, using [Act. Mat.:C_{65}:SEBS] = [40:50:10] electrode formulations and 1.0M $LiPF_6$ in EC:DEC (1:1, v:v) as electrolyte.

SI-II-4-10 Galvanostatic cycling poly(HHAQ-glutaraldehyde) in lithium metal battery

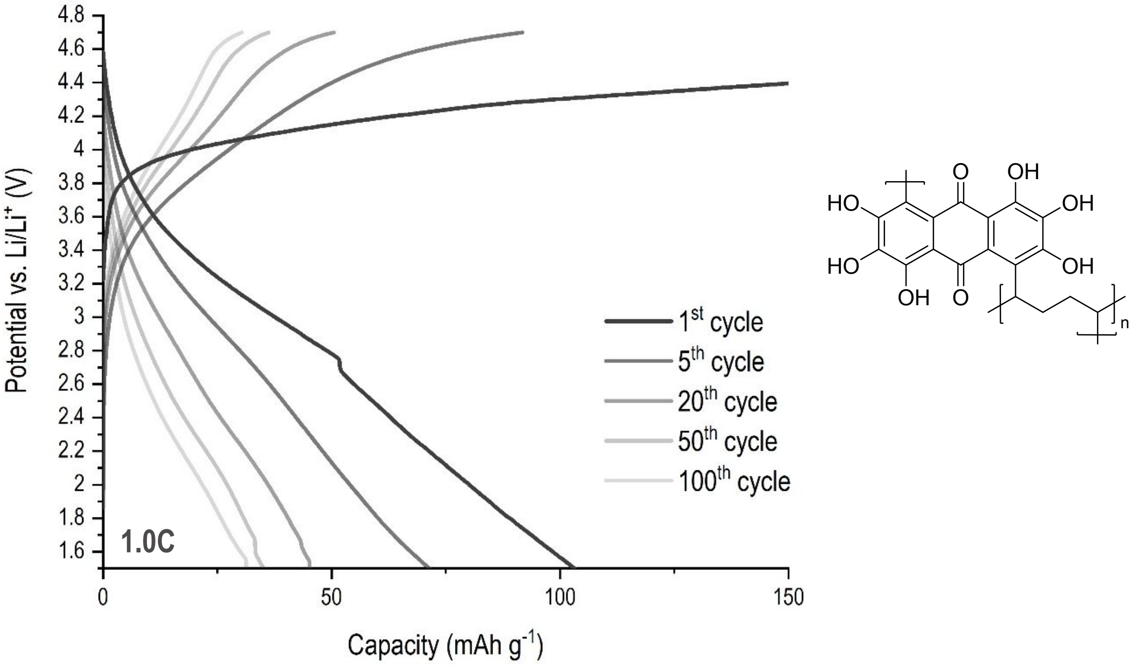

Figure S41. The 1st, 5th, 20th, 50th and 100th galvanostatic charge-discharge cycle (C-rate = 1.0C) of poly(HHAQ-glutaraldehyde) in a lithium metal battery, using [Act. Mat.:C_{65}:SEBS] = [40:50:10] electrode formulations and 1.0M $LiPF_6$ in EC:DEC (1:1, v:v) as electrolyte.

SI-II-4-11 Galvanostatic cycling initial poly(HHAQ-formaldehyde) in lithium metal battery

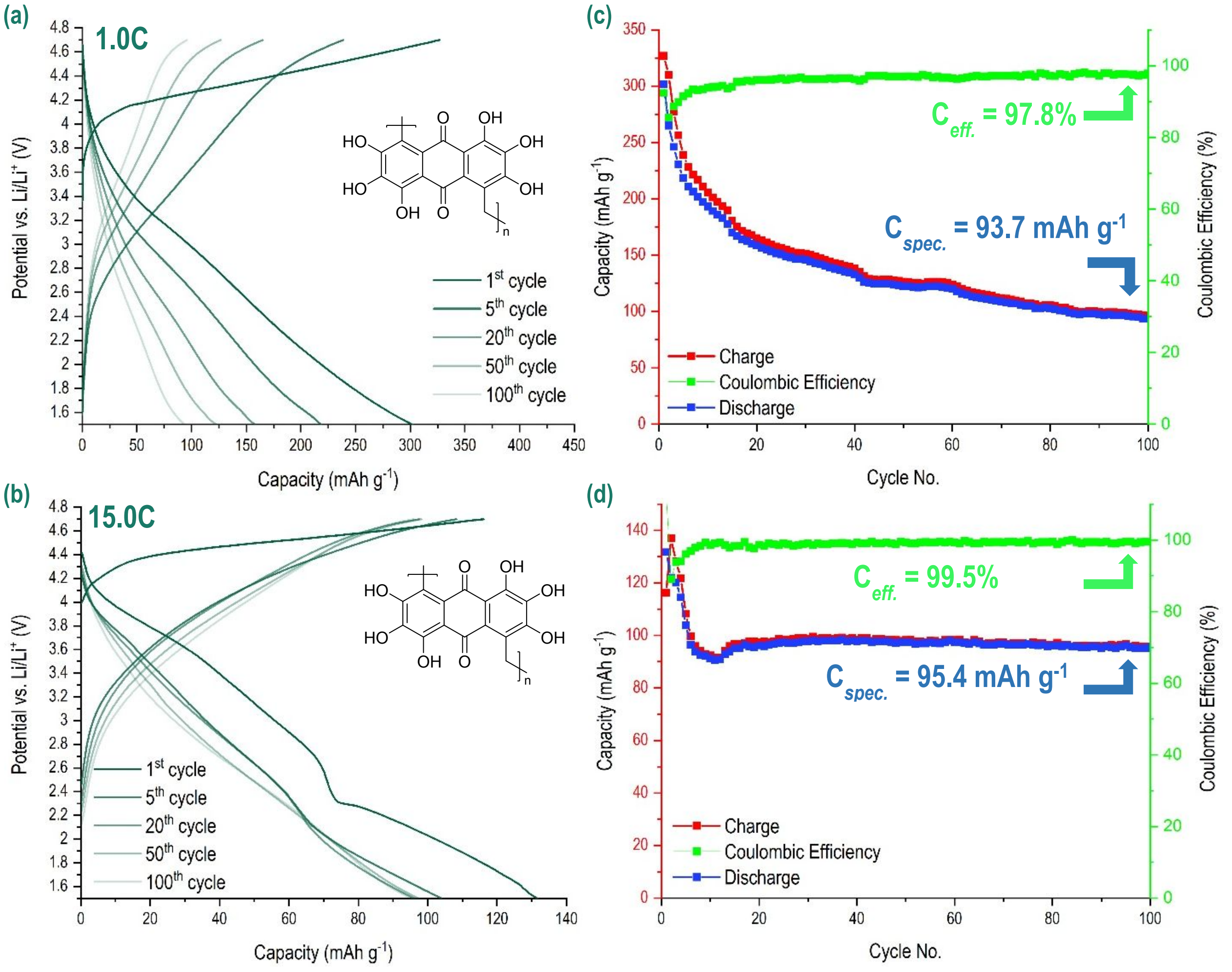

Figure S42. The 1st, 5th, 20th, 50th and 100th galvanostatic charge-discharge cycles of initial poly(HHAQ-formaldehyde)-10wt.%MWCNTs composite in lithium metal battery, using [Act. Mat.:C_{65}:CMC] = [40:55:5] electrode formulations and 1.0M $LiPF_6$ in EC:DEC (1:1, v:v) as electrolyte, at (**a**) 1.0C and **(b)** 15.0C. **(c)** and **(d)** depict the charge and discharge capacity as well as the coulombic efficiency (C_{eff}) over 100 cycles for the initial poly(HHAQ-formaldehyde)-10wt.%MWCNTs composite at 1.0C and 15.0C, respectively.

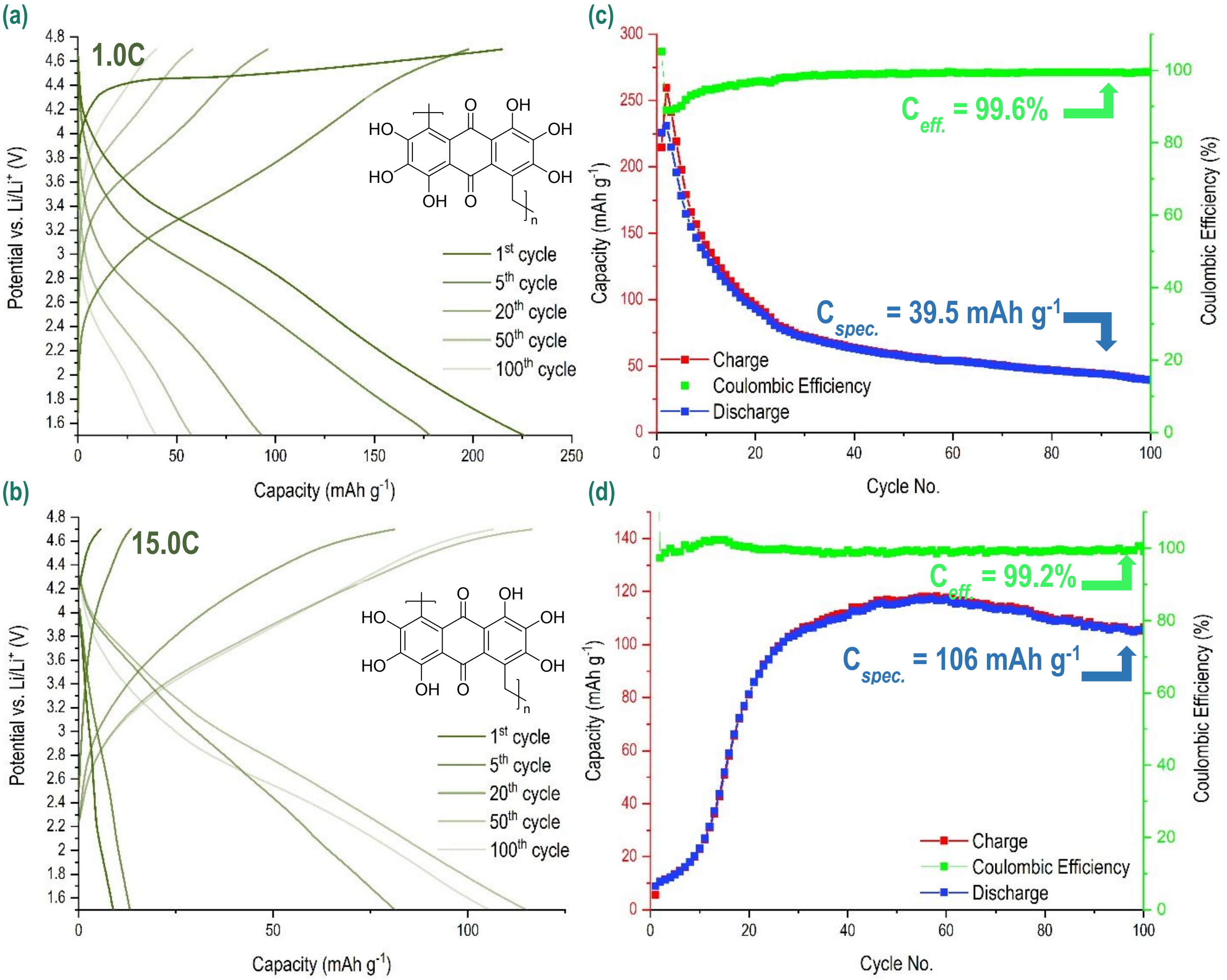

Figure S43. The 1st, 5th, 20th, 50th and 100th galvanostatic charge-discharge cycles of Initial poly(HHAQ-formaldehyde) in lithium metal battery, using [Act. Mat.:C_{65}:CMC] = [40:55:5] electrode formulations and 1.0M $LiPF_6$ in EC:DEC (1:1, v:v) as electrolyte, at (**a**) 1.0C and **(b)** 15.0C. **(c)** and **(d)** depict the charge and discharge capacity as well as the coulombic efficiency (C_{eff}) over 100 cycles for the initial poly(HHAQ-formaldehyde) at 1.0C and 15.0C, respectively.

SI-II-4-12 Cyclic Voltammogram of poly(HHAQ-formaldehyde) in 1.0M $LiPF_6$ in EC:DEC (1:1, v:v)

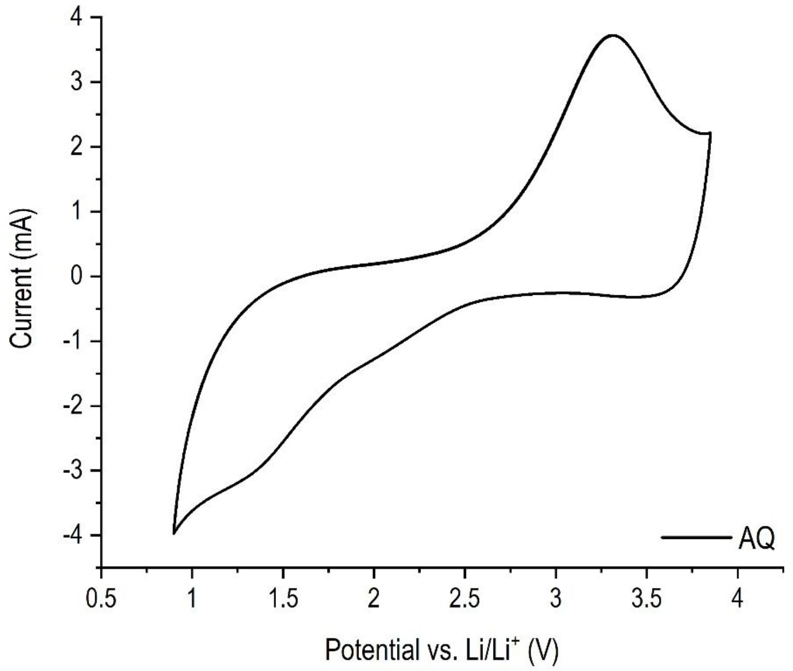

Figure S45. Cyclic Voltammogram (scan rate = 5 mV/s) of the anthraquinone subgroup of poly(HHAQ-formaldehyde) in a three-electrode setup using Li metal as counter and reference electrode and a [Act. Mat.:C_{65}:SEBS] = [40:50:10] electrode formulation on Glassy Carbon as working electrode with 1.0M $LiPF_6$ in EC:DEC (1:1, v:v) as electrolyte.

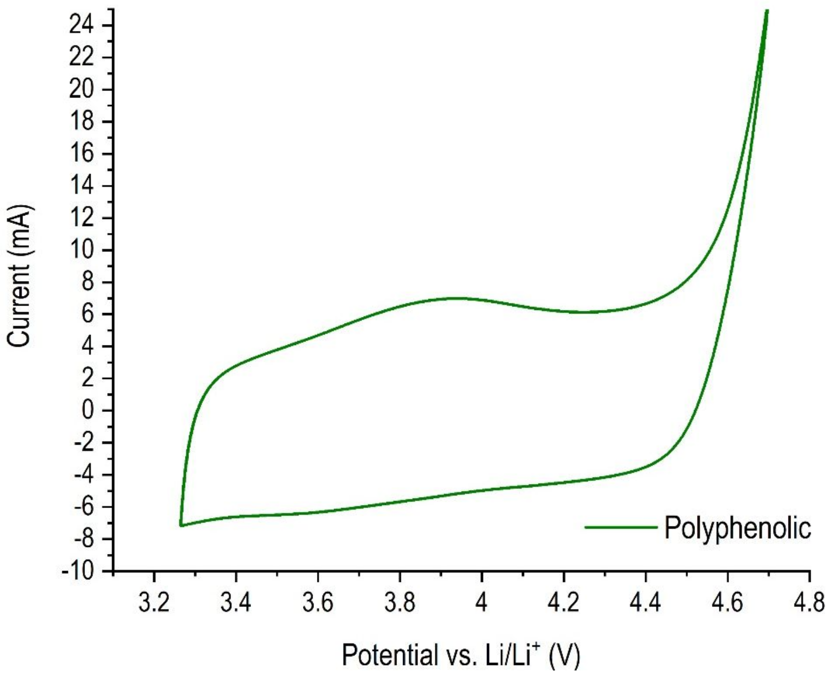

Figure S44. Cyclic Voltammogram (scan rate = 5 mV/s) of the polyphenolic subgroup of poly(HHAQ-formaldehyde) in a three-electrode setup using Li metal as counter and reference electrode and a [Act. Mat.:C_{65}:SEBS] = [40:50:10] electrode formulation on Glassy Carbon as working electrode with 1.0M $LiPF_6$ in EC:DEC (1:1, v:v) as electrolyte.

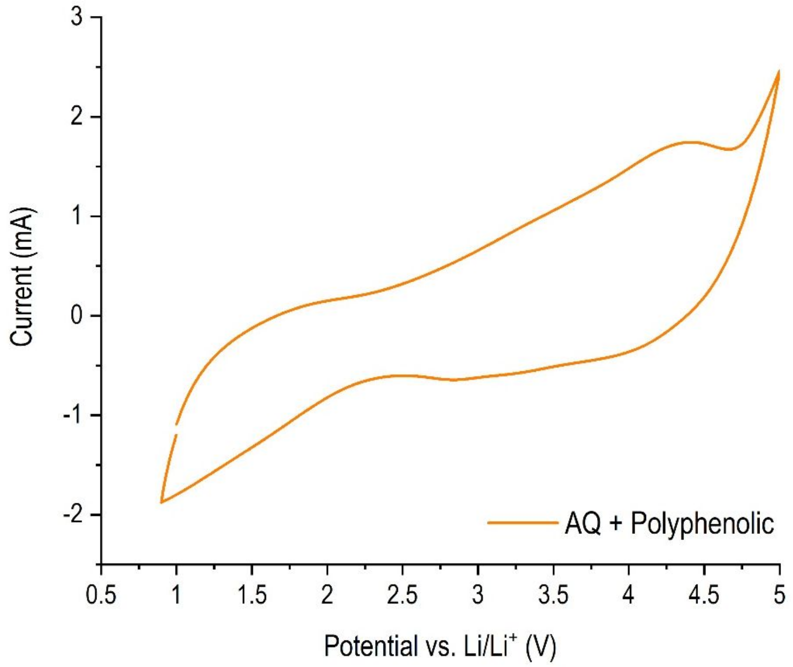

Figure S46. Cyclic Voltammogram (scan rate = 5 mV/s) of the anthraquinone and polyphenolic subgroup of poly(HHAQ-formaldehyde) in a three-electrode setup using Li metal as counter and reference electrode and a [Act. Mat.:C_{65}:SEBS] = [40:50:10] electrode formulation on Glassy Carbon as working electrode with 1.0M $LiPF_6$ in EC:DEC (1:1, v:v) as electrolyte.

SI-II-4-13 Decomposed post-cycling poly(HHAQ-formaldehyde) electrode

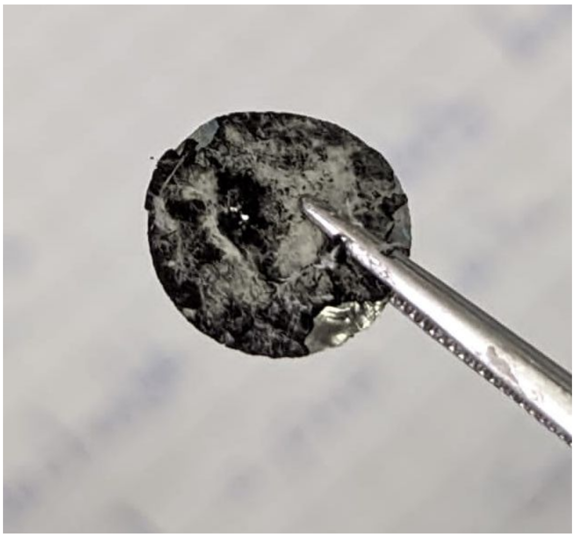

Figure S47. Post-cycling poly(HHAQ-formaldehyde) decomposed electrode obtained for lithium metal battery after cycling 100 cycles using 1.0M $LiPF_6$ EC:DEC (1:1, v:v) as electrolyte.

SI-II-4-14 dQ/dV plot of poly(HHAQ-formaldehyde)-10wt.%MWCNTs

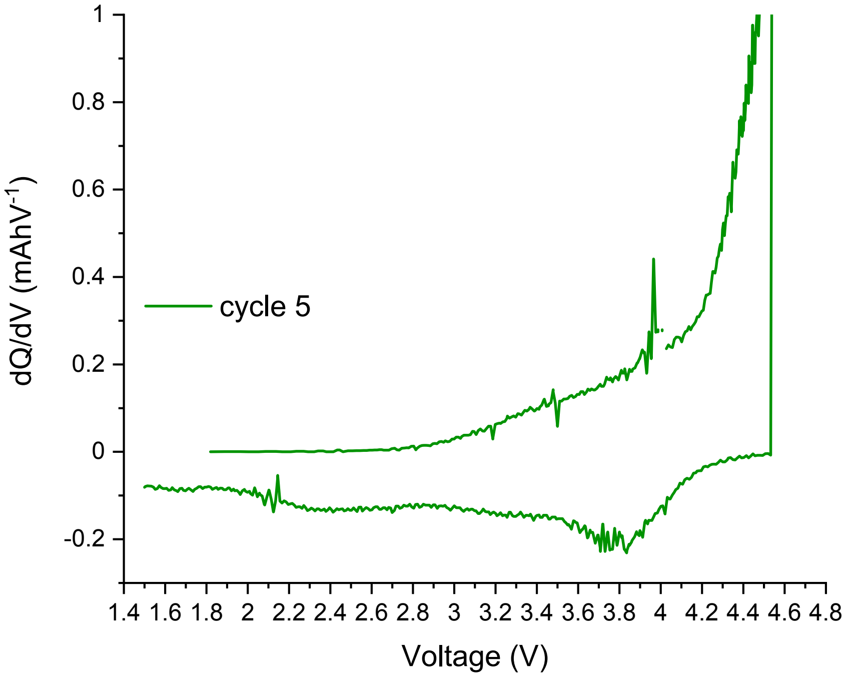

Figure S48. The dQ/dV plot of the 5th galvanostatic charge-discharge cycles poly(HHAQ-formaldehyde)-10wt.%MWCNTs composite in lithium metal battery, using [Act. Mat.:C_{65}:SEBS] = [40:50:10] electrode formulations and 1.0M $LiPF_6$ in EC:DEC (1:1, v:v) as electrolyte, at 1.0C.

SI-II-4-15 dQ/dV plot of poly(THAQ-formaldehyde)-10wt.%MWCNTs

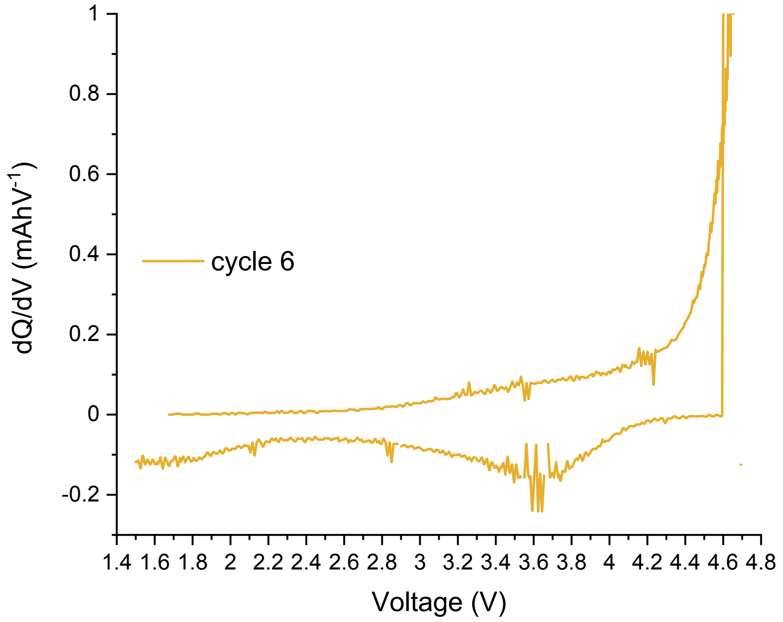

Figure S49. The dQ/dV plot of the 6th galvanostatic charge-discharge cycles poly(THAQ-formaldehyde)-10wt.%MWCNTs composite in lithium metal battery, using [Act. Mat.:C65:SEBS] = [40:50:10] electrode formulations and 1.0M LiPF6 in EC:DEC (1:1, v:v) as electrolyte, at 1.0C/

References

[1] Isse, A. A.; Gennaro, A. Absolute Potential of the Standard Hydrogen Electrode and the Problem of Interconversion of Potentials in Different Solvents. *J. Phys. Chem. B.* **2010,** *114* (23), 7894-7899. DOI: 10.1021/jp100402x

[2] Robiquet. Ueber Die Gallussäure. *Ann. der Pharm.* **1836**, *19* (2), 204–210. https://doi.org/https://doi.org/10.1002/jlac.18360190212.

[3] Billard, J.; Luz, Z.; Poupko, R.; Zimmermann, H. The Mesophases of Octa-Alkanoyloxy-9,10-Anthraquinone. *Liq. Cryst.* **1994**, *16* (2), 333–342. https://doi.org/10.1080/02678299408029157.

[4] Boldt, P. Ein Neues Chinonsystem: Derivate Des Amphi-Anthrachinons. *Chem. Ber.* **1967**, *100* (4), 1270–1280. https://doi.org/https://doi.org/10.1002/cber.19671000428.

[5] Rehse, K.; Kawerau, H.-G. Untersuchungen Über Den Mechanismus Der Reaktion von Aromaten Mit Marquis Reagens. *Arch. Pharm. (Weinheim).* **1974**, *307* (12), 934–942. https://doi.org/https://doi.org/10.1002/ardp.19743071208.
